# Substitutions of Saturated Fatty Acids From Different Meats With Dairy and Incident Relationship With Cardiovascular Diseases: The UK Biobank Prospective Study

**DOI:** 10.1161/JAHA.125.042289

**Published:** 2025-12-03

**Authors:** Yakima D. Vogtschmidt, Sabita S. Soedamah‐Muthu, David I. Givens, Julie A. Lovegrove

**Affiliations:** ^1^ Hugh Sinclair Unit of Human Nutrition, Department of Food and Nutritional Sciences University of Reading Reading UK; ^2^ Institute for Cardiovascular and Metabolic Research University of Reading Reading UK; ^3^ Institute for Food, Nutrition and Health University of Reading Reading UK; ^4^ Center of Research on Psychological Disorders and Somatic Diseases (CoRPS), Department of Medical and Clinical Psychology Tilburg University Tilburg the Netherlands

**Keywords:** cardiovascular diseases, dairy, meat, saturated fatty acids, substitution, Diet and Nutrition, Cardiovascular Disease, Epidemiology, Lifestyle

## Abstract

**Background:**

Evidence on the associations with cardiovascular diseases (CVD) for the substitution of saturated fatty acids (SFA) from meats with SFA from dairy and meats remains uncertain, especially for unprocessed and processed poultry. This study aims to model theoretical substitution of SFA from meats with SFA from dairy and meats with incident CVD.

**Methods:**

A total of 120 496 UK Biobank participants (57% female, mean age: 55.9 ± 7.8 years) without prevalent CVD and with ≥2 24‐hour dietary assessments were included. Multivariable adjusted Cox regressions were used to estimate CVD risk (9890 cases) for the substitution of equivalized SFA from meat with dairy (2.5% of energy), over a median follow‐up of 10.5 years.

**Results:**

Substitution equivalized SFA from total meat with total dairy was associated with lower CVD risk (hazard ratio [HR], 0.91 [95% CI, 0.86–0.96]). Strong inverse associations were found for the substitution of SFA from processed poultry with milk (HR, 0.67 [95% CI, 0.45–0.99), yogurt (HR, 0.58 [95% CI, 0.38–0.87), and cheese (HR, 0.64 [95% CI, 0.43–0.94) and for the substitution of SFA from processed total or poultry with unprocessed total (HR, 0.87 [95% CI, 0.76–0.98) or poultry meats (HR, 0.61 [95% CI, 0.40–0.93), respectively. Analyses involving whole food groups, adjusted for total SFA intake, showed similar results.

**Conclusions:**

Associations between major SFA sources and CVD may not be solely driven by their SFA content but also by other nutrients within these foods and their food matrices. Although our data do not prove causality, it could contribute to the evidence base to inform more specific dietary guidance, particularly in relation to processed meat consumption.

**Nonstandard Abbreviations and Acronyms**
En%: proportion (%) of total energy intake
EPIC: European Prospective Investigation into Cancer and Nutrition
MUFA: monounsaturated fatty acids
PUFA: polyunsaturated fatty acids
SFA: saturated fatty acids

**Clinical Perspective**

**What Is New?**

- In a large population‐based cohort study of British men and women, there was a significantly lower risk of cardiovascular diseases with modeled substitution of saturated fatty acids from total meat, processed meat (total, red and poultry meat), and unprocessed red meat with saturated fatty acids from dairy foods and for the substitution of saturated fatty acids from processed meats or processed poultry with unprocessed meats or unprocessed poultry, respectively.

**What Are the Clinical Implications?**

- The healthiness of foods does not only depend on their saturated fatty acids content, but also on other nutrients within these foods and their food matrix effects, which supports analyses of whole foods, rather than the nutrients they contain, and development of food‐based dietary recommendations.
- Our findings support current recommendation to limit processed red and poultry meat intake, with possible benefits of dairy foods, but also suggest the importance of considering processed and unprocessed types of meats separately in the future analyses and in the current recommendations.

Cardiovascular diseases (CVD) are the leading cause of global mortality, accounting for an estimated 19.8 million (34%) deaths in 2022.^1^ In the United Kingdom, CVD caused approximately a quarter (27%) of all deaths and affected over 7.6 million people in 2025,^2^ with coronary heart diseases (CHD) and cerebrovascular diseases (CVA) being the most frequent CVD types.^2^ Dietary guidelines for cardiovascular health, issued by the UK Scientific Advisory Committee on Nutrition, suggest reducing the consumption of saturated fatty acids (SFA) to no more than 10% of total energy intake (en%) daily and to reduce the intake of red and processed meats to 70 g/day (for those who consume >90 g/day of red and processed meats).^3^, ^4^, ^5^ However, the advice on the types of meats within the recommended limit is still not clear.^6^

Although the recommendation to reduce red and processed meat intake and to increase plant‐based foods has been increasingly promoted,^7^, ^8^, ^9^ with supportive evidence,^10^ the impact of dairy foods remain unclear and controversial. Dairy foods are the main sources of SFA intake in the UK diet^11^, yet consistently show neutral or inverse associations with CVD risk.^12^, ^13^, ^14^, ^15^ Conversely, meat and meat products, also being major contributors of SFA,^11^ showed high risk associations with CVD, particularly unprocessed red and processed meat.^16^, ^17^, ^18^ In an isoenergetic context, a higher intake of a certain food is always accompanied with a lower intake of another food. In nutrition epidemiological studies, the “joint estimate” for the risk of diseases of such association can be estimated using a food substitution model, which is regarded as particularly useful to inform dietary guidance.^19^ Substitution of meat‐derived SFA with dairy‐derived SFA has consistently been shown to be associated with lower risk of CVD, but total meat and total dairy were analyzed in the substitution models, not individual types of meat and dairy foods, which may associate differently with CVD risk.^20^, ^21^, ^22^, ^23^

With data from the EPIC (European Prospective Investigation into Cancer and Nutrition) ‐Norfolk Study^23^, we previously modeled the isoenergetic substitution of SFA from red meat, processed meat, and poultry with milk, yogurt, and cheese in relation to incident CVD, CHD, and CVA among 21 841 UK men and women.^23^ Over a median follow‐up of 21 years, substitution of 2.5en% of SFA from red meat with cheese was found to be associated with lower CVD incidence (hazard ratio [HR], 0.86 [95% CI, 0.76–0.97]). These findings were confirmed when substituting SFA from processed meats with cheese, showing similar associations, with a lower risk of CVD (HR, 0.77 [95% CI, 0.68–0.88]), CHD (HR, 0.77 [95% CI, 0.66–0.90]), and CVA (HR, 0.81 [95% CI, 0.67–0.99]). Beneficial associations were also found for substitution of SFA from processed meat with milk with incident CVD (HR, 0.84 [95% CI, 0.75–0.94]) and CHD (HR, 0.83 [95% CI, 0.73–0.95]). However, for the substitution of SFA from poultry with milk (HR, 2.06 [95% CI, 1.09–3.89]), yogurt (HR, 1.96 [95% CI, 1.04–3.70]), and cheese (HR, 2.55, [95% CI, 1.27–5.13]), a higher incidence of CVA was observed. These findings not only suggest the possible benefits of substituting red and processed meats with dairy foods for CVD risk, but also the importance of considering types of meat in the analyses, based on animal origin (red versus white) and level of processing (unprocessed versus processed).^23^ However, this study was limited by factors, including the use of dietary data from 1990s,^23^ which may not reflect current UK dietary patterns^23^ and the lack of more extensive categorization of meats (total meat, red meat by processing level), especially of white meats (unprocessed versus processed), for which little evidence is available.^24^

Our aim is to model the substitution of types of meats, red meats, and poultry with types of dairy, equivalized for SFA content, and incident CVD, CHD, and CVA among participants from the UK Biobank, providing a strong evidence base for the meat substitution benefits, using more recent dietary data. We examined the associations of substituting processed meats with unprocessed meats and red meat with poultry, equivalized for SFA, with CVD outcomes, as previous research suggests differential associations between processed and unprocessed meats,^25^, ^26^ and between red and white/poultry meat.^24^, ^27^, ^28^ We hypothesized beneficial or neutral CVD associations for the substitution of red and processed meats with dairy products; for the substitution of processed with unprocessed meats and for the substitution of red with white/poultry meat, equivalized for SFA.

## Methods

### Data Availability Statement

Due to the material transfer agreement with the UK Biobank, full study data cannot be shared openly. Data from the UK Biobank can be accessed by applying through the UK Biobank Access Management System (www.ukbiobank.ac.uk/register‐apply). This research has been conducted using the UK Biobank Resource, under Application Number 101928.

### Study Design and Participants

We used data from the UK Biobank Study, a large prospective population‐based cohort study that recruited >500 000 men and women, aged 40 to 69 years, from 22 assessments centers in England, Scotland, and Wales, between 2006 and 2010 (baseline).^29^ At baseline, participants completed a self‐administered touchscreen questionnaire and underwent a nurse‐guided verbal interview, in which they provided information on sociodemographic characteristics (eg, ethnicity, education), lifestyle behaviors (eg, smoking, physical activity), family history of major diseases, and health status (eg, medical history, medication use). In addition, participants underwent physical measurements (eg, anthropometrics), and data were collected with trained staff, using standardized procedures. The UK Biobank obtained approval from the UK Northwest Multi‐Centre Research Ethics Committee (11/NW/0157). All participants gave informed consent to participate and to provide health outcome data at follow‐up, which were obtained through data linkage to hospital inpatient admission and mortality records. More detailed information on the study protocol can be found elsewhere^30^ and access to the data has been granted by the UK Biobank Access Subcommittee.

### Dietary Assessment

Dietary intake was assessed using self‐administered, web‐based dietary questionnaire (Oxford WebQ). The questionnaire was completed at the assessment center, at the end of the recruitment phase (2009–2010), and online on 4 separate occasions, between February 2011 and June 2012. Further information on this tool and methods used to estimate the SFA intake for each individual food source can be found in Methods S1. We included participants who completed at least 2 24‐hour dietary assessments in our analyses and estimated the SFA intake for the following meat and dairy categories: total meat, unprocessed meat, processed meat, total red meat, unprocessed red meat, processed red meat, total poultry, unprocessed poultry and processed poultry, total dairy, milk, yogurt, and cheese. Definitions of each category can be found in Table S1.

### CVD Ascertainment

We ascertained the incidence of CHD and CVA and composite CVD, defined as first occurrence of any CHD or CVA event,^23^ to ensure higher event rates and conduct well‐powered statistical analyses. CHD was defined according to the *International Classification of Diseases, Tenth Revision* (*ICD‐10*) codes: I20‐I25 and cerebrovascular diseases included *ICD‐10* codes I60‐I69 (Table S2). Incident and prevalent cases included nonfatal events as primary and secondary diagnosis from hospital inpatient registries and fatal events as primary and contributory causes of death from death registries of each CVD outcome. For our longitudinal analyses of incident CVD, we excluded participants with prevalent CVD before the date of last completed 24‐hour dietary assessment (baseline) (Figure S1).

Data on the date and cause of hospital admissions were obtained through linkage to the Health Episode Statistics for England, Patient Episode Database for Wales and Scottish Morbidity Records for Scotland. Information on the date and cause of death was provided by the National Health Service England for England and Wales and the National Health Service Central register, part of the National Records of Scotland, for Scotland. At the time of these analyses, hospital admissions data were available to October 31, 2022 for England; August 31, 2022 for Scotland and May 31, 2022 for Wales. Mortality data were available to November 30, 2023 for England and Wales and December 31, 2023 for Scotland. For our analyses, we applied the earliest censoring date for each country for all outcomes to capture incident cases across the same follow‐up period.

### Covariates

Potential confounders were identified based on existing literature,^20^, ^21^, ^22^, ^23^ biological plausibility, and statistical efficacy. The following variables were extracted and compiled from the baseline assessment center visit between 2006 and 2010: sociodemographic factors (age, sex, ethnic background, education, Townsend deprivation index, country of assessment center), lifestyle factors (smoking, physical activity, dietary supplement use, alcohol intake), female factors (hormone replacement therapy, menopausal status), family history and cardiometabolic risk factors (family history of CVD, body mass index [BMI], waist circumference, baseline hypertension, baseline hypercholesterolemia, baseline diabetes). Data were self‐reported on touchscreen questionnaires or verbal interviews, except for BMI or waist circumference, which were measured by trained staff using standardized protocols. BMI was calculated as kg/m^2^. Participant’s weight was measured using Tanita BC 418 MA body composition analyzer (Tanita, Tokyo, Japan) and standard scales, without shoes or outdoor clothing. Weight values from the body composition analyzer were used for the analyses. If missing, values from the standard scales were used. Height, in a barefoot standing position, was measured using Seca 202 device, to the closest centimeter (cm). Waist circumference was measured using the Wessex nonstretchable sprung tape measurement, to the closest centimeter (cm). Townsend deprivation index is a composite indicator of participants’ socioeconomic status, based on their home ownership, car ownership, employment status, and household crowdedness.^31^ Each participant was assigned a score, which corresponded to the output area from the preceding national census, in which their postcode is located. Data on dietary covariates, including fiber intake, fruit and vegetables intake and intake of protein, carbohydrate, monounsaturated fatty acids (MUFA), polyunsaturated fatty acids (PUFA), SFA from other foods (neither the substituted or substitution food), trans fatty acids, dietary cholesterol, and total energy intake were obtained from at least 2 24‐hour dietary assessments and collected between 2009 and 2012. As the proportion of missing values of the covariates was low (<6%), we created a missing value category for the variable, where needed, to preserve sample size and decrease the risk of selection bias. More detailed information on the covariates can be found in Table S3.

### Statistical Analysis

Cohort characteristics were evaluated as means ±SD for continuous variables and as column percentages (%) (counts) for categorical variables in the overall cohort and across different categories of percentage of energy from SFA from different meat and dairy categories. We calculated pairwise Spearman correlation coefficients for the relationship between and within the estimates of SFA from the different meat and dairy types and estimates of the corresponding food groups and created a heat plot (correlation matrix) to visualize the relationships (Figure S2).

We used Cox proportional hazard regression models to compute the HR and the 95% CI, using years of study as underlying timescale. SFA from total and different types of meat and dairy were adjusted for total energy intake by means of the nutrient density method (expressed as proportion [%] of en%) (Text S1) and modeled continuously in increments of 2.5 en%. We chose this incremental amount based on a previous study,^22^ The Cox model was converted into an isoenergetic nutrient substitution model,^32^ in which the estimated differences in risk of CVD between individuals were investigated, for a 2.5 en% lower intake of SFA from different meats and a concomitant 2.5en% higher intake of SFA from dairy or meats.

The following model was specified for the substitution of, for instance, SFA from total meat with total dairy:

$$\begin{array}{c} \log\left(h\left(t;x\right)\right)=\log\left(h0\left(t\right)\right)\\ \;+\beta_{1}\;\mathrm{SFA}\;\text{from total dairy}\;\left(2.5\;\mathrm{en}\%\right)\\ \;+\beta_{2}\;\mathrm{SFA}\;\text{from total meat}\;\left(2.5\;\mathrm{en}\%\right)\\ \;+\beta_{3}\prime \;\text{Other macronutrients}\;\left(\mathrm{en}\%\right)\\ \;+\beta_{4}\prime \;\text{Covariates} \end{array}$$

exp (*β*_1_ − *β*_2_) provided the HR representing the hypothetical effect of increasing 2.5 en% of SFA from total dairy, at the expense of 2.5 en% of SFA from total meat, on the incidence of CVD outcomes, while keeping other covariates and the intake of other macronutrients constant. The 95% CI was estimated using the variance and covariance matrices.^19^

Person‐years of follow‐up were calculated from the date of the last completed 24‐hour dietary assessment until the earliest date of; incident CVD, death (not due to the outcomes of interest) or end of censoring. Multivariable‐adjusted models included adjustments for age, sex, ethnic background, education, Townsend deprivation index, country of assessment center, smoking, physical activity, alcohol intake, dietary supplement use, hormone replacement therapy use (women only), menopausal status (women only), fruit and vegetables, fiber, family history of CVD, BMI, waist circumference, baseline hypertension, baseline hypercholesterolemia, baseline diabetes, protein, carbohydrate, MUFA, PUFA, SFA from other foods (not the substituted and substitution food), trans fatty acids, and dietary cholesterol. We did not adjust for total energy intake because protein, carbohydrate, MUFA, PUFA, and SFA from other foods were highly correlated with total energy intake (r_s_ ≥ 0.67) and therefore introduce collinearity. As part of the sensitivity analyses, we constructed another substitution model that included the same confounders as in the model but with total energy intake included and without inclusion of protein, carbohydrate, MUFA, PUFA, and SFA from other foods, trans fatty acids, and dietary cholesterol. The reason for this is, because in real‐life settings, substitution of a meat with a dairy product cannot be performed, while keeping the intake of other macronutrients (eg, protein) and micronutrients (eg, iron, calcium) constant. The proportionality of the hazards for the exposures with all outcomes were assessed using Schoenfeld residuals test; assumptions were not violated. We also assessed the associations of substituting 2.5 en% of SFA from a specific type of meat with another, to assess potential differences in the associations between meat types.

To detect potential effect modifiers, multiplicative interaction terms between SFA variables with sex, age, BMI, waist circumference, baseline hypertension, baseline hypercholesterolemia, and baseline diabetes were included in the substitution model. Further stratified analyses were conducted, to assess potential heterogeneity in the associations and differences across subgroups in the study population. We also conducted 7 sensitivity analyses to assess the robustness of our findings: (1) to assess if the associations were driven by the SFA or the food group, we conducted analyses on food group level, using the same model as in the main analyses, adjusted for total SFA intake; (2) to examine potential reverse causality, we excluded incident cases in the first 2 years of follow‐up; (3) for the same reason, we also excluded participants with baseline hypercholesterolemia and diabetes, as presence of these conditions could change participants’ lifestyle and dietary behavior; (4) to assess if the validity of the dietary data had been compromised, we repeated our analyses, after excluding participants who reported to be ill or fasting during the 24‐hour dietary assessments; (5) as completion of multiple 24‐hour dietary assessments better represents habitual dietary intake, we also restricted our analyses to those who completed three or more 24‐hour dietary assessments; (6) we repeated our analyses using age as underlying time variable, to assess if using this time metric would result in changes in the estimates; and (7) as some ethnic minority groups (eg, South Asian, Black African, and African Caribbean) have significantly higher risk of CVD, compared with the general UK population,^33^ we repeated our analyses after excluding these groups (n = 4168, 3.5% of the total population). All analyses were conducted using Stata, version 18. *P* values (2 sided) of <0.05 were considered as statistically significant.

## Results

### Participant Characteristics

Our study cohort consisted of 120 496 participants after excluding participants who withdrew consent (n = 149), who completed <2 24‐hour dietary assessments (n = 375 412), who had a prevalent CVD before the last completed 24‐hour dietary assessment (baseline) (n = 5716), who died before baseline (n = 1), who had missing information on date of completing the 24‐hour dietary assessment (n = 2), and who had implausible energy intake values (n = 540) (Figure S1). In the study population, 37.8% (n = 45 562) completed 2, 33.5% (n = 40 403) completed 3, 24.1% (n = 29 017) completed 4, and 4.6% (n = 5514) completed 5 24‐hour dietary assessments. 57% of the total population were female. The mean age was 55.9 ± 7.8 years and 40.5% and 18.9% of the participants had overweight and obesity, respectively (Table 1). On average, 11.7 ± 2.9 en% were derived from SFA (Table 2), of which 3.4 ± 2 en% were derived from dairy products and 1.6 ± 1.3 en% were derived from meat and meat products (Table 2). SFA from total dairy was weakly inversely correlated with SFA from total meat (r_s_ = −0.10) (Figure S2). SFA from total dairy was moderately positively correlated with its corresponding food group (r_s_ = 0.39), whereas SFA from total meat was highly positively correlated with its corresponding food group (r_s_ = 0.78) (Figure S2).

**Table 1:** Baseline Cohort Characteristics in the Study Population, the UK Biobank Study (n = 120 496)*

	Total
No.	120 496
Sociodemographic factors	
Age, y	55.9 ± 7.8
Female sex	57 (68689)
White European participants	96.5 (116328)
College or university degree/vocation	57 (68708)
Least deprived	21.8 (26322)
Country of assessment center	
England	91.5 (110298)
Scotland	5.3 (6430)
Wales	3.1 (3768)
Lifestyle/dietary factors	
Current smoker	6.9 (8259)
Physical activity (excess metabolic equivalents of task)	
Low <10	27.8 (33477)
Moderate 10–50	53.1 (63957)
High ≥50	17.1 (20660)
Dietary supplement use	52.5 (63210)
Alcohol intake (g/d)	
Nondrinker	5.6 (6792)
<1	10.7 (12888)
1–<10	26.9 (32399)
10–<20	24.9 (29957)
≥20	26.3 (31717)
Female factors	
Hormone replacement therapy use†	19.9 (23941)
Menopausal†	33.4 (40305)
Family history/cardiometabolic risk factors	
Family history of cardiovascular disease	56.2 (67709)
Body mass index categories (kg/m^2^)	
Underweight <18.5	0.6 (709)
Healthy weight 18.5–25	39.7 (47869)
Overweight 25–30	40.5 (48849)
Obesity 30<	18.9 (22814)
Abdominal obesity	27.9 (33591)
Baseline hypertension	24.1 (29063)
Baseline hypercholesterolemia	12.9 (15583)
Baseline diabetes	2.3 (2760)

* Data are presented as means ± SD for continuous variables or as % (n) for categorical variables, with % representing column percentages.

^†^ Women only.

**Table 2:** Baseline Intake of Energy, Nutrients and Food Groups in the Study Population, the UK Biobank Study (n = 120 496)*

	Total
No.	120 496
Energy and nutrient intake	
Total energy intake (kcal/d)	2057 ± 498
Total protein (en%)	15.9 ± 3
Total carbohydrate (en%)	49.2 ± 7.4
Total fat (en%)	31.8 ± 5.8
Total monounsaturated fatty acids (en%)	11.5 ± 2.4
Total polyunsaturated fatty acids (en%)	5.7 ± 1.6
Total SFA (en%)	11.7 ± 2.9
SFA from food source (en%)	
Total dairy	3.4 ± 2
Milk	0.9 ± 0.7
Yogurt	0.2 ± 0.4
Cheese	1.4 ± 1.4
Total meat	1.6 ± 1.3
Unprocessed meat	0.6 ± 0.6
Processed meat	0.5 ± 0.7
Red meat	0.8 ± 0.8
Unprocessed red meat	0.8 ± 1.0
Processed red meat	0.4 ± 0.7
Poultry	0.3 ± 0.4
Unprocessed poultry	0.2 ± 0.3
Processed poultry	0.0 ± 0.1
Cereal and cereal products	2.7 ± 1.6
Fat spreads	1.2 ± 1.4
Vegetable and vegetable dishes	0.7 ± 0.7
Sugar, sweet spreads, preserves	0.6 ± 0.9
Eggs and egg products	0.4 ± 0.6
Soups, savory sauces, samosa, pakora, sushi	0.4 ± 0.4
Fish and fish products	0.3 ± 0.4
Savory snacks	0.2 ± 0.3
Nuts and seeds	0.2 ± 0.4
Total trans unsaturated fatty acid (en%)	0.5 ± 0.2
Fiber (g/d)	17.9 ± 5.8
Sodium (mg/d)	1953 ± 658
Food group intake (g/day)	
Fruit and vegetables	383.8 ± 225.7
Total dairy	316.3 ± 168.3
Milk	230 ± 148.2
Yogurt	43.8 ± 53.2
Cheese	17.6 ± 18.1
Total meat	91.2 ± 61.2
Unprocessed meat	67.8 ± 52.9
Processed meat	21.3 ± 27.9
Red meat	55.3 ± 48.9
Unprocessed red meat	37.1 ± 41.3
Processed red meat	18.1 ± 24.6
Poultry	33.8 ± 41.3
Unprocessed poultry	30.6 ± 39.5
Processed poultry	3.2 ± 12.9

en% indicates proportion of total energy intake; and SFA, saturated fatty acids.

* Data are presented as means ± SD.

Participants with the highest SFA intake from total dairy were older, more likely to be female and White European, and more likely to have a college or university degree/vocation, compared with those with the lowest intakes (Table S4). There was a lower proportion of current smokers, a higher proportion of dietary supplement users, and a lower consumption of fruit and vegetables in the highest compared with the lowest category of dairy SFA intake. Participants with the highest compared with lowest SFA consumption from dairy, had a more favorable cardiometabolic risk profile, with lower proportions of participants with overweight and obesity, abdominal obesity, baseline hypertension, baseline hypercholesterolemia, and baseline diabetes (Table S4). Generally, similar distributions were observed with SFA consumption from milk, yogurt, and cheese (Tables S5–S7). By contrast, the characteristics across consumption SFA from total meat showed an association in the opposite direction. Participants with the highest compared with lowest total meat SFA intake were less likely to be female, White European, and to have a college or university degree/vocation (Table S8). These participants generally showed less favorable lifestyle behaviors, with higher proportion of current smokers, lower proportion of participants with moderate or high physical activity levels, lower proportion of dietary supplement users, higher consumption of alcohol, and lower consumption of fruit and vegetables and fiber, compared with those in lowest category of meat SFA intake. Moreover, participants in the highest category were more likely to have overweight and obesity, more likely to have abdominal obesity, and more likely to have baseline hypertension, hypercholesterolemia, and diabetes, compared with the lowest intake category (Table S8). We observed similar distribution in characteristics with SFA intake from unprocessed, processed, red meat, unprocessed red meat, processed red meat, poultry, unprocessed poultry, and processed poultry (Tables S9‐S16).

### Substitution of Total Meat (Total, Unprocessed, Processed) With Types of Dairy, Equivalized for SFA, and Incident Cardiovascular Diseases

**Figure 1:**
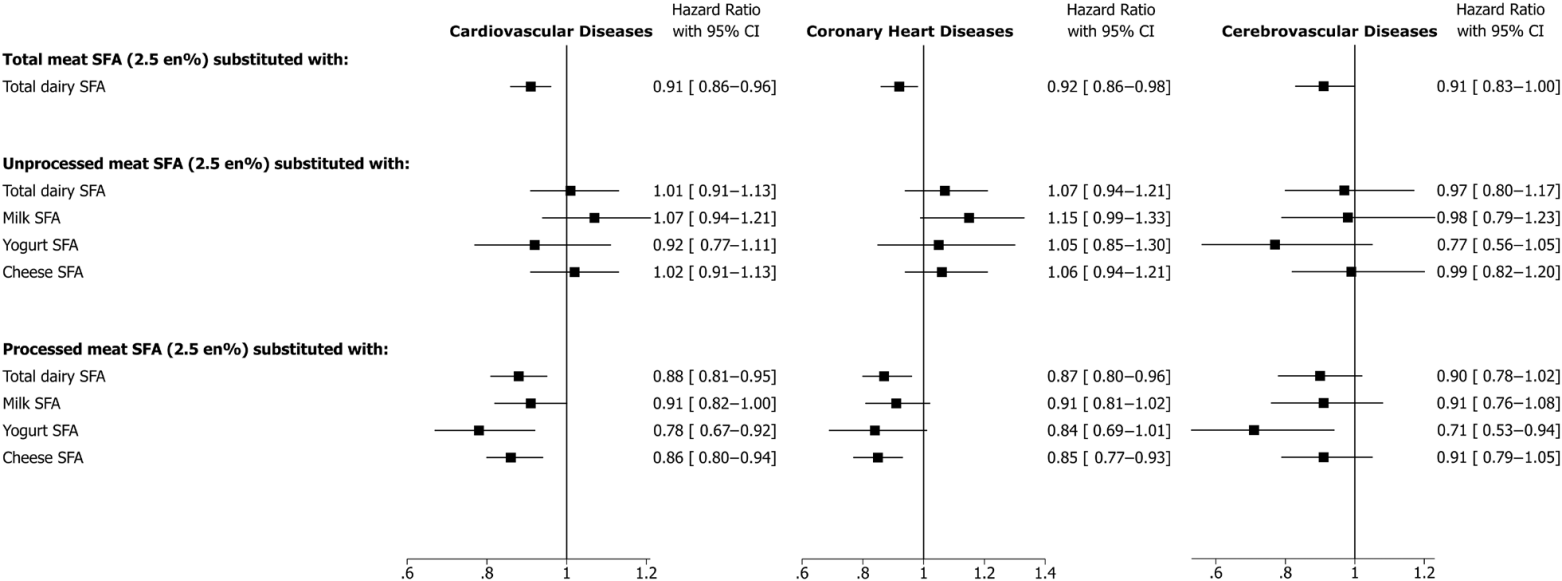
Hazard ratio and 95% CIs for cardiovascular diseases, coronary heart diseases, and cerebrovascular diseases associated with substitution of SFA from total, unprocessed and processed meats with total dairy, milk, yogurt, and cheese. Cox proportional models were adjusted for age, sex, ethnic background, education, Townsend deprivation index, country of assessment center, smoking, physical activity, alcohol intake, dietary supplement use, hormone replacement therapy use (women only), menopausal status (women only), fruit and vegetables, fiber, dietary cholesterol, trans fatty acids, protein, carbohydrate, monounsaturated fatty acids, polyunsaturated fatty acids, saturated fatty acids from other foods (except for the substitution foods), family history of cardiovascular disease body mass index, waist circumference, baseline hypertension, baseline hypercholesterolemia, baseline diabetes. No. of incident cases/person‐years of follow‐up for cardiovascular diseases: 9890/1 214 957; for coronary heart diseases: 7216/1 223 821; for cerebrovascular diseases: 3315/1 268 585. En% indicates proportion (%) of total energy intake; and SFA, saturated fatty acids.

During a median follow‐up of 10.5 years, a total of 9890 (8.2%) incident cases of CVD (person‐years: 1 214 957); 7216 (5.9%) cases of CHD (person‐years: 1 223 821) and 3315 (2.8%) cases of CVA (person‐years: 1 268585) were identified. In the multivariable‐adjusted analyses, substitution of 2.5 en% of SFA from total meat with total dairy was associated with lower incidence of CVD (HR, 0.91 [95% CI, 0.86–0.96]) and CHD (HR, 0.92 [95% CI, 0.86–0.98]), and in a similar direction for CVA (HR, 0.91 [95% CI, 0.83–1.00]) (Figure 1).

For CVD, beneficial statistically significant associations were observed for the substitution of SFA from processed meat with total dairy (HR, 0.88 [95% CI, 0.81–0.95]), yogurt (HR, 0.78 [95% CI, 0.67–0.92]) or cheese (HR, 0.86 [95% CI, 0.80–0.94]), and in a similar direction for milk (HR, 0.91 [95% CI, 0.82–1.00]) (Figure 1). Similar associations were seen for CHD and CVA (Figure 1). We found no associations with any CVD outcome for the substitution of SFA from unprocessed meat (red and white) with any type of dairy (Figure 1).

### Substitution of Red Meat (Total, Unprocessed, Processed) With Types of Dairy, Equivalized for SFA, and Incident Cardiovascular Diseases

**Figure 2:**
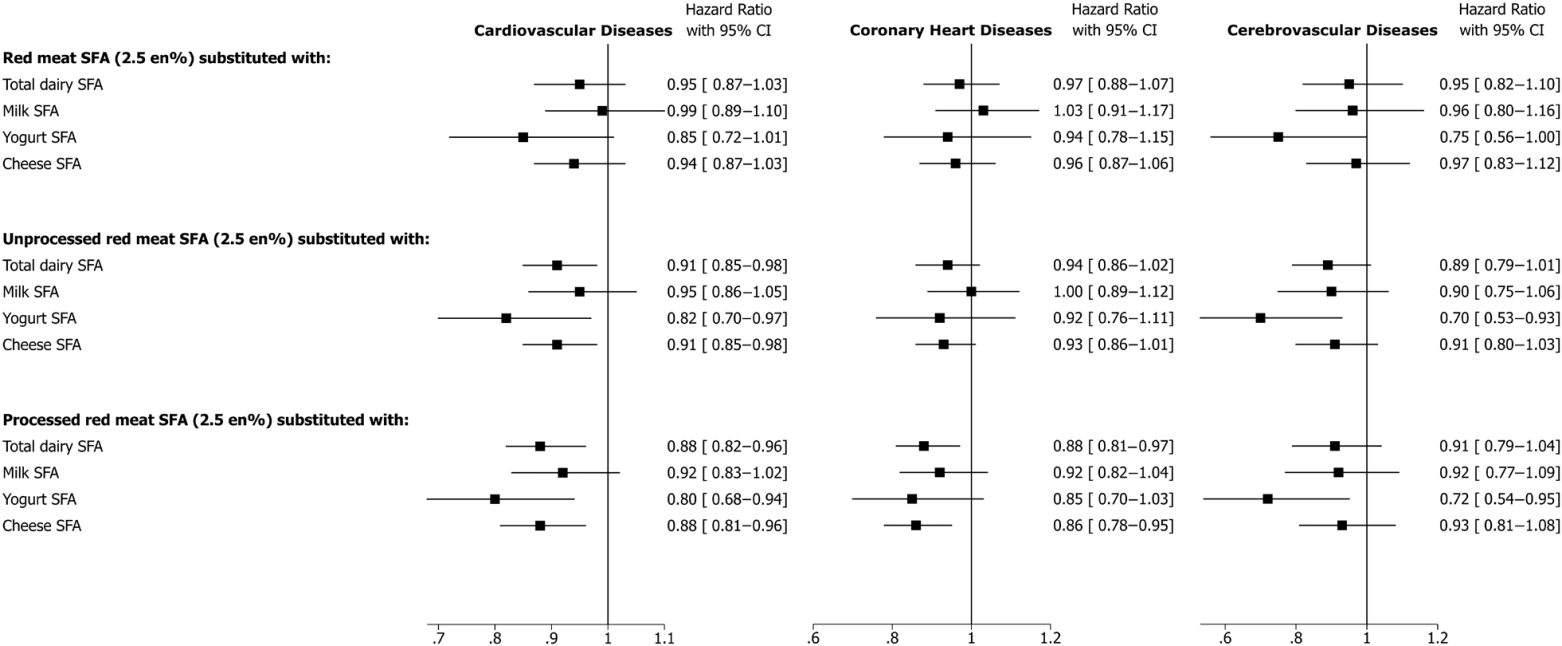
Hazard ratio and 95% CIs for cardiovascular diseases, coronary heart diseases, and cerebrovascular diseases associated with substitution of SFA from total, unprocessed and processed red meats with total dairy, milk, yogurt, and cheese. Cox proportional models were adjusted for age, sex, ethnic background, education, Townsend deprivation index, country of assessment center, smoking, physical activity, alcohol intake, dietary supplement use, hormone replacement therapy use (women only), menopausal status (women only), fruit and vegetables, fiber, dietary cholesterol, trans fatty acids, protein, carbohydrate, monounsaturated fatty acids, polyunsaturated fatty acids, saturated fatty acids from other foods (except for the substitution foods), family history of cardiovascular disease, body mass index, waist circumference, baseline hypertension, baseline hypercholesterolemia, baseline diabetes. No. of incident cases/person‐years of follow‐up for cardiovascular diseases: 9890/1 214 957; for coronary heart diseases: 7216/1 223 821; for cerebrovascular diseases: 3315/1 268 585. En% indicates proportion (%) of total energy intake; and SFA, saturated fatty acids.

We found no significant associations with CVD, CHD, and CVA for the substitution of SFA from total red meat with any type of dairy in the adjusted models (Figure 2). However, in a detailed analyses by types of red meats, substitution of SFA from unprocessed red meat with total dairy (HR, 0.91 [95% CI, 0.85–0.98]), yogurt (HR, 0.82 [95% CI, 0.70–0.97]), or cheese (HR, 0.91 [95% CI, 0.85–0.98]) was associated with a significantly lower incidence of CVD (Figure 2). Similarly, beneficial CVD associations were seen when SFA from processed red meats were substituted with total dairy (HR, 0.88 [95% CI, 0.82–0.96]) or yogurt (HR, 0.80 [95% CI, 0.68–0.94]) or cheese (HR, 0.88 [95% CI, 0.81–0.96]). The associations were similar, in terms of direction for CHD and CVA, although not all reached statistical significance (Figure 2). We observed a significantly lower CHD incidence with substitution of SFA from processed red meat with total dairy (HR, 0.88 [95% CI, 0.81–0.97]) or cheese (HR, 0.86 [95% CI, 0.78–0.95]) and in a similar direction for yogurt (HR, 0.85 [95% CI, 0.70–1.03]) (Figure 2). For CVA, the data suggested inverse associations with substitution of SFA from unprocessed red meat (HR, 0.70 [95% CI, 0.53–0.93]) or processed red meat (HR, 0.72 [95% CI, 0.54–0.95]) with yogurt (Figure 2).

### Substitution of Poultry (Total, Unprocessed, Processed) With Dairy, Equivalized for SFA, and Incident Cardiovascular Diseases

**Figure 3:**
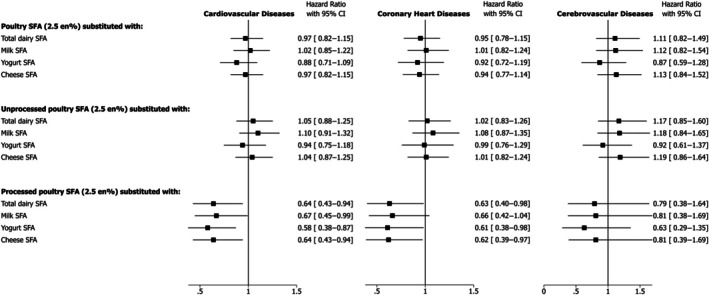
Hazard ratio and 95% CIs for cardiovascular diseases, coronary heart diseases, and cerebrovascular diseases associated with substitution of SFA from total, unprocessed and processed poultry with total dairy, milk, yogurt, and cheese. Cox proportional models were adjusted for age, sex, ethnic background, education, Townsend deprivation index, country of assessment center, smoking, physical activity, alcohol intake, dietary supplement use, hormone replacement therapy use (women only), menopausal status (women only), fruit and vegetables, fiber, dietary cholesterol, trans fatty acids, protein, carbohydrate, monounsaturated fatty acids, polyunsaturated fatty acids, saturated fatty acids from other foods (except for the substitution foods), family history of cardiovascular disease, body mass index, waist circumference, baseline hypertension, baseline hypercholesterolemia, baseline diabetes. No. of incident cases/person‐years of follow‐up for cardiovascular diseases: 9890/1 214 957; for coronary heart diseases: 7216/1 223 821; for cerebrovascular diseases: 3315/1 268 585. En% indicates proportion (%) of total energy intake; and SFA, saturated fatty acids.

There was a significantly lower incidence of CVD with substitution of SFA from processed poultry with total dairy (HR, 0.64 [95% CI, 0.43–0.94]), milk (HR, 0.67 [95% CI, 0.45–0.99]), yogurt (HR, 0.58 [95% CI, 0.38–0.87]), and cheese (HR, 0.64 [95% CI, 0.43–0.94]). Similarly, associations in a downward trend were observed for CHD and CVA (Figure 3). However, for the substitution of SFA from unprocessed poultry with any dairy type, no significant associations with all CVD outcomes were found (Figure 3).

### Substitution of Different Types of Meat, Equivalized for SFA, and Incident Cardiovascular Diseases

**Figure 4:**
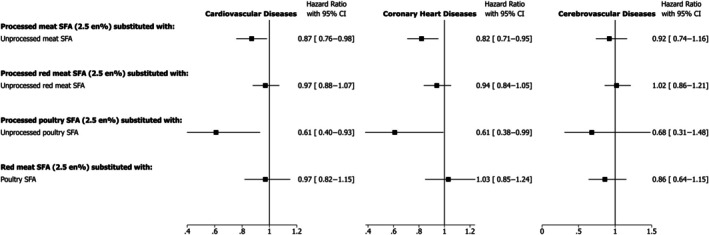
Hazard ratio and 95% CIs for cardiovascular diseases, coronary heart diseases, and cerebrovascular diseases associated with substitution of SFA from one type of meat with another. Cox proportional models were adjusted for age, sex, ethnic background, education, Townsend deprivation index, country of assessment center, smoking, physical activity, alcohol intake, dietary supplement use, hormone replacement therapy use (women only), menopausal status (women only), fruit and vegetables, fiber, dietary cholesterol, trans fatty acids, protein, carbohydrate, monounsaturated fatty acids, polyunsaturated fatty acids, saturated fatty acids from other foods (except for the substitution foods), family history of cardiovascular disease, body mass index, waist circumference, baseline hypertension, baseline hypercholesterolemia, baseline diabetes. No. of incident cases/person‐years of follow‐up for cardiovascular diseases: 9890/1 214 957; for coronary heart diseases: 7216/1 2238 21; for cerebrovascular diseases: 3315/1 268 585. En% indicates proportion (%) of total energy intake; and SFA, saturated fatty acids.

Substitution models involving different types of meat showed that substituting SFA from processed meats with unprocessed meats was inversely associated with the incidence of CVD (HR, 0.87 [95% CI, 0.76–0.98]) and CHD (HR, 0.82 [95% CI, 0.71–0.95]) (Figure 4). Specifically, substitution of SFA from processed poultry with unprocessed poultry was associated with lower incidence of CVD (HR, 0.61 [95% CI, 0.40–0.93]) and CHD (HR, 0.61 [95% CI, 0.38–0.99]). No other significant associations were found (Figure 4).

### Sensitivity Analyses

Results were similar when we used another substitution model that included total energy intake, instead of separate nutrients such as protein, carbohydrate, MUFA, PUFA, and SFA from other foods, trans fatty acids, and dietary cholesterol (Tables S17 and S18). Results from food substitution models (using actual food groups for the substitution models instead of SFA from foods), adjusting for total SFA, showed that substituting 1 serving of total meat (100 g), with 1 serving of total dairy (100 g), was associated with significant lower incidence of CVD and CHD, and similarly nonsignificantly associated with cerebrovascular diseases, confirming our results (Table S19). The substitution of 1 serving of processed meats (red and white) (50 g), red meat (100 g), unprocessed red meat (100 g), processed red meat (50 g), processed poultry (50 g) with 1 serving of total dairy (100 g), milk (200 g), yogurt (125 g), or cheese (30 g) were each associated with significant lower CVD incidence. Similar trends in associations were found for CHD and CVA, although the estimates were not all statistically significant (Table S19). The effect estimates became attenuated but remained inverse, for the substitution of 1 serving of processed meat (50 g) with 1 serving of unprocessed meat (100 g) and 1 serving of processed poultry (50 g) with 1 serving of unprocessed poultry (100 g) (Table S20). Results were confirmed by all other sensitivity analyses (Tables S21 and S22), except for substitution of SFA from processed poultry with total and subtypes of dairy with incident CVD and CHD and for the substitution of SFA from processed meat with unprocessed meat and processed poultry with unprocessed poultry. The estimates became nonsignificant when analyses were restricted to participants who completed 3 24‐hour dietary assessments or more, which could be due to reduced power to detect associations within this subsample (Tables S21 and S22).

### Potential Effect Modifiers

Further analyses to check for effect‐modification confirmed our previous results. Based on the evaluation of the *P* value for interaction and comparison of the direction and magnitude of the associations in each stratum, we found no clear evidence of an interaction of each exposure variable with each potential effect modifier in relation to incident CVD outcome (Tables S23 and S24).

## Discussion

In our study population of 120 496 middle‐aged UK men and women, isoenergetic substitution of SFA from total meat with total dairy was associated with 9% and 8% lower risk of CVD and CHD, respectively, independent of sociodemographic, lifestyle behaviors, dietary, family history of CVD, and cardiometabolic risk factors. More detailed SFA substitution analyses showed inverse associations with CVD for the substitution of processed meats (red or poultry) and unprocessed red meat, but not unprocessed poultry, with dairy products. In particular, we found relatively strong inverse associations for the substitution of SFA from processed poultry with total and subtypes of dairy, which has not been investigated and demonstrated previously. Associations comparing substitutions within types of meats showed a significant lower incidence of CVD with substitution of SFA from processed meat with unprocessed meat and with substitution of SFA from processed poultry with unprocessed poultry. Relatively strong effect estimates were observed, considering the low mean and narrow range of SFA intake from unprocessed and processed poultry in this cohort. Results were similar when substitutions of whole food groups were performed, using models as in the main analyses, with additional adjustment for total SFA. These findings suggest that the associations between SFA‐rich foods and health may not be only determined by their SFA content, but also by other factors including nutrients within these foods and the food matrix, which should be considered in future studies and dietary guidelines.

Substitution of equivalent SFA from total meat with total dairy were found to be associated with lower incidence of CVD and CHD, confirming previous results.^20^, ^21^, ^22^, ^23^ Our finding that substitutions of equivalent SFA from red meat or processed meats (total, red, or poultry) with dairy products was associated with lower CVD risk, is also in line with previous results.^23^, ^34^, ^35^, ^36^, ^37^ In the EPIC‐Norfolk Study, substitutions of SFA from red or processed meats with dairy (cheese or milk) were associated with lower CVD incidence.^23^ The EPIC Study among 409 885 men and women in 9 European countries, including United Kingdom, showed that substitutions of 100 kcal/day of red and processed meat with 100 kcal/day of yogurt (HR, 0.84 [95% CI, 0.76–0.92]) and cheese (HR, 0.85 [95% CI, 0.79–0.92]) were associated with significantly lower incidence of CHD and borderline significant with milk (HR, 0.95 [95% CI, 0.90–1.00]).^34^ This study showed similar effect estimates, although separate analyses for red meat and processed meat were not performed. Other prospective cohort studies among US adults also showed inverse associations with CHD and stroke with substitution of 1 serving of red meat with 1 serving of dairy,^35^, ^36^ supporting results of another study among 43 272 US men showing beneficial associations with CHD incidence, with substitution of 1 serving of red meat with 1 serving of milk (HR, 0.90 [95% CI, 0.85–0.96]), yogurt (HR, 0.78 [95% CI, 0.64–0.94]), or cheese (HR, 0.89 [95% CI, 0.82–0.98]).^37^ However, these studies were conducted among health professionals for which results may be less generalizable to people from the whole population.

The novelty of our study is the inclusion of processed poultry, as separate food group in the substitution models, allowing direct comparisons of associations of this food group with different types of dairy and other types of meats. Our findings do support the proposition that different types of meat are differentially associated with CVD risk.^27^, ^28^ This is further supported by our finding that substitution of SFA from processed meats with unprocessed meats was associated with lower risk of CVD and CHD, suggesting potential differential associations of processed and unprocessed meats with CVD. Similarly, we observed beneficial CVD associations with substitution of the same SFA content of processed poultry with unprocessed poultry, which, to our knowledge, has not been demonstrated previously. To evaluate potential harmful or beneficial associations of meat intake with CVD risk, more research into the associations (with and without substitution) of processed and unprocessed meats, and processed and unprocessed poultry, on CVD risk is needed. The latter is particularly important given the increased intake of poultry meat and products (such as breaded chicken pieces) that has occurred in recent times.

There are different explanations for the observed beneficial associations with CVD risk for the substitution of the equivalent amount of SFA from red and processed meats with dairy products and for processed poultry with unprocessed poultry. As this study was observational, no causality can be inferred, and confounders could play a role. First, participants with the highest consumption of SFA from dairy products were generally more likely to have a healthier lifestyle and favorable cardiovascular risk profile, compared with those with the lowest consumption, whereas participants with higher consumption of SFA from meat products had characteristics profiles in the opposite direction. The same applies to participants with consumption of SFA from unprocessed poultry, who were less likely to be a current smoker, compared with nonconsumers, whereas participants with consumption of processed poultry were more likely to be a current smoker, compared with zero consumers. However, we adjusted for a range of lifestyle (eg, smoking, physical activity) and cardiometabolic factors in our analyses and the inverse associations remained.

Several mechanisms have been proposed in the literature, but these are suggested mechanisms and are still not proven, which calls for further research. For example, the observed inverse associations might be due to the role of specific nutrients in dairy products and meat products in the food matrix. Dairy products contain bioavailable calcium, magnesium, and potassium,^38^ which may have antihypertensive effects,^39^ as well as lactotripeptides, which have been demonstrated to lower blood pressure, through angiotensin converting enzyme‐inhibitory activity.^40^ Also, part of the dairy matrices has been proposed to beneficially affect cardiovascular health, including dairy‐specific fatty acids (eg, odd‐chain SFA and branched chain fatty acids) and fraction of the milk fat globule membrane (polar lipids).^41^, ^42^ The fermented yogurt matrix may beneficially impact the gut microbiome^42^ and the cheese matrix rich in calcium, proteins (mostly casein), and bioactive fatty acids has been found to favorably affect blood lipid markers,^43^ attenuate inflammatory markers, and improve vascular function.^42^ Conversely, specific components in red meat, such as heme iron and l‐carnitine metabolites including trimethylamine‐N‐oxide, have been suggested to promote atherosclerosis,^44^, ^45^ increasing the risk of CVD. The presence of high levels of heme iron, sodium, nitrites, nitrates (N‐nitroso compounds), and preservatives in processed meats could potentially explain the harmful associations observed between processed meat intake and CVD risk.^46^, ^47^, ^48^, ^49^ Possible explanation for the observed beneficial associations with CVD for the substitution of equivalent amount of SFA from processed meats or processed poultry with unprocessed meats or unprocessed poultry could be the difference in preservatives such as the sodium content between processed and unprocessed meats, but more evidence is needed to be able to explain these results.

Strengths of this study include the large sample size, prospective nature, large number of incident cases of CVD, detailed covariate data with small proportion of missing values, and detailed dietary data collected over an extended period, allowing estimation of SFA intake from different types of meat and dairy. For poultry, data were available for unprocessed and processed poultry, providing novel insights into the associations of these with CVD incidence, which could be helpful to inform dietary advice.

Several limitations need to be considered when interpreting the findings. First, results on substitution impacts are based on estimates from statistical models, not from actual substitutions in a human experimental setting. Therefore, we are unable to infer causality for the observed associations. However, the use of isoenergetic substitution models enabled us to compare the associations of different food groups, with specification of the comparison food group, which is of interest and relevant to inform public health recommendations. Second, we observed moderate to high correlations between estimates of SFA from meat and dairy with the corresponding food groups, which made it difficult to separate the effects of SFA from the food group itself. Therefore, we performed within food substitution analyses, in which 1 serving of a specific type of meat was substituted with 1 serving of a specific type of dairy, adjusted for total SFA, where the associations remained statistically significant, suggesting that the associations may not be driven solely by the SFA, but components within the food matrices other than SFA. Third, our study relied on self‐reported dietary data, which are inherently imprecise and prone to bias. The degree of bias could not only be linked to participants’ inability to recall and estimate their food intake but also to participant’s obesity status. In our study population, 40.5% and 18.9% were overweight and obese. People who have overweight or obesity tend to underreport their dietary intake or intake of foods that are considered as “unhealthy.” This factor could bias our findings toward the null. Fourth limitation is the risk of measurement errors due to reliance on data from short‐term, baseline 24‐hour dietary assessments, which do not capture long‐term dietary exposure. Fifth, despite our rigorous adjustment for potential confounding factors in our models, the possibility of residual confounding cannot be ruled out in studies with observational design. Sixth, our findings may be limited in terms of generalizability to the whole UK population or elsewhere, as the cohort mainly consisted of White participants, who were less likely to have obesity, had fewer self‐reported health conditions, and had healthier lifestyle and higher socioeconomic status.^50^

## Conclusions

Our findings suggest that different SFA‐rich foods associate differently with CVD risk, which could be due to other nutrients within these foods, other than SFA, and their food matrix effects. Theoretical substitution analyses suggest that a lower intake of SFA from processed meats (total, red, or poultry), or unprocessed red meat and a concomitant higher intake of SFA from dairy products may be associated with a lower risk of CVD, which aligns with the general recommendation to limit processed meat intake and to have optimal intake of dairy products. Additionally, our findings suggest that unprocessed meat and unprocessed poultry, in contrast to processed meat and processed poultry, respectively, could be beneficial to cardiovascular health, suggesting the need for separate consideration of processed and unprocessed meats in future analyses and in the current dietary recommendations Current dietary recommendations are largely based on existing evidence from meta‐analyses of observational studies, similar to our study, and partly on randomized controlled trials. Replication studies, in other populations, with an observational and randomized controlled trial design, are needed to confirm our findings.

## Sources of Funding

Yakima D. Vogtschmidt has received funding for her PhD studentship from the Rank Prize Funds, the Dutch Dairy Association and the Danish Dairy Research Foundation. Sabita S. Soedamah‐Muthu has received unrestricted grants from the Dutch Dairy Association. These organizations played no role in the design, collection, analyses, interpretation or writing of the results, nor were they involved in the decision to publish.

## Disclosures

Yakima D. Vogtschmidt has received a grant for PhD studentship from the Rank Prize Funds, the Dutch Dairy Association, and the Danish Dairy Research Foundation for the submitted work. Sabita S. Soedamah‐Muthu has received unrestricted grants from the Dutch Dairy Association, outside the submitted work. David I. Givens has received travel expenses and honoraria in connection with lectures and meetings from the UK Dairy Council (now Dairy UK), the Dutch Dairy Association, the European Dairy Association, the International Dairy Federation, Centre National Interprofessionnel de l’Industrie Laitière, and the US Dairy Research Institute. He has also been a consultant to the Estonian Bio‐Competence Centre of Healthy Dairy Products and to the Dairy UK on fats in dairy products and cardiometabolic diseases. Julie A. Lovegrove is the Deputy Chair of the UK government’s Scientific Advisory Committee on Nutrition (SACN). This research is independent of SACN and does not necessarily reflect the views of SACN.

## Supporting information

Methods S1
Tables S1–S24
Figures S1–S2
References 51–57
