# Supplementary material for: Substitutions of Saturated Fatty Acids From Different Meats With Dairy and Incident Relationship With Cardiovascular Diseases: The UK Biobank Prospective Study
Source: J Am Heart Assoc. 2025 Dec 3;14(24):e042289. doi: 10.1161/JAHA.125.042289 (PMC12826886; doi:10.1161/JAHA.125.042289)
Supplement: Supplementary file 1 — Methods S1 Tables S1–S24 Figures S1–S2 References 51–57 [file JAH3-14-e042289-s001.pdf]

SUPPLEMENTAL MATERIAL

Substitution of saturated fatty acids from different meats with dairy and incident relationship with cardiovascular diseases: the UK Biobank Prospective Study

Yakima D Vogtschmidt et al.

Correspondence to: Professor Julie A. Lovegrove. Email: j.a.lovegrove@reading.ac.uk. Hugh Sinclair Unit of Human Nutrition, Department of Food and Nutritional Sciences, Harry Nursten, University of Reading, Harry Nursten, Reading RG6 6DZ, UK.

Methods S1. Dietary assessment and estimation of SFA intakes per food source

Dietary data were collected using an Oxford WebQ, a 24hr-dietary assessment, used to ask participants to report their food and beverage consumption over the past 24 hours (1). 206 types of foods and 32 types of beverages were included. The validity of this questionnaire has previously been assessed, against biomarkers for protein, potassium, total sugars and total energy expenditure, estimated by accelerometery, and has proven to be relatively accurate in estimating true intakes of these nutrients (2). The correlation between the mean value from two Oxford WebQ and the estimated true intakes was 0.47 for protein; 0.39 for potassium; 0.40 for total sugars; 0.38 for energy intake (2). The Oxford WebQ was also compared to an interview-based 24hr-dietary recall and was found to capture similar food items, estimates and nutrient intakes for a 24hr-dietary intake (1). The correlation estimates between the two methods were 0.58 for total energy; 0.57 for total fat; 0.64 for saturated fat and 0.54 for polyunsaturated fat (2). Although food intake estimates get more precise with more completion of 24hr-dietary assessments, the group of people with higher number of completed 24hr-dietary assessments (e.g., three or more) was smaller and had different characteristics, compared to the baseline group, thus increasing the risk of selection bias (3). We therefore included participants who completed at least two 24hr-dietary assessments in our analysis.

The intake of energy, saturated fatty acids (SFA) and other nutrients from each food were calculated by multiplying the amount consumed by the nutrient composition of each food, using the UK Nutrient Databank (UKND) Food Composition Table (FCT) from survey year 6, which included FCT for years 2012-2013 and 2013-2014 (4). We used SFA intake data, based on previously defined food groups (n=93). These food groups were based on dietary data from 24hr-dietary assessments and classified based on nutrient composition, culinary use, processing and plant/animal origin (5). The estimates of the intakes of SFA (in g/day) were converted to SFA estimates in kcal by multiplying the SFA intakes (in g/day) by 9. The resulting values were subsequently divided by the estimated total energy intakes and multiplied by 100%, giving the percentage of the proportion of SFA of the total energy intake (en%).

Table S1. Definitions of the meat and dairy sources of saturated fatty acids in the UK Biobank cohort

Saturated fatty acids from food source	Food items
**Total dairy**	Milk; yogurt; cheese; cream; milk-dairy desserts (incl. ice cream, milk puddings, milk-based desserts, cheesecake)
**Milk**	Whole milk (incl. milk>3.6 g fat per 100 g (cow, goat, sheep)); Semi-skimmed milk (incl. milk>1 g fat per 100 g (cow, other)); skimmed milk (incl. milk<1 g fat per 100 g (cow, cholesterol lowering, powdered)); milk-based & powdered drinks (incl. dairy-based smoothies, milk-based drinks, hot chocolate)
**Yogurt**	Whole milk yogurt (plain); fat free and lower fat yogurt, plain or flavoured
**Cheese**	Cheese >17.5 g fat per 100 g, including hard cheese, soft cheese, spreadable, Blue, Feta, Mozzarella, Goats, other); Cheese <=17.5g fat per 100 g, including hard and spreadable lower fat cheese, Cottage
**Total meat**	Unprocessed meat; processed meat; other meat (incl. offal)
**Unprocessed meat**	Unprocessed red meat; unprocessed poultry
**Processed meat**	Processed red meat; processed poultry
**Red meat**	Unprocessed red meat; processed red meat
**Unprocessed red meat**	Beef (incl. roast, stew, mince, curry, burgers); pork (incl. roast, chops, sweet and sour); lamb or mutton (incl. roast, chops, stew, burgers)
**Processed red meat**	Sausages, bacon (with and without fat), ham, liver pâté
**Poultry**	Unprocessed poultry; processed poultry
**Unprocessed poultry**	Poultry (with/without skin)
**Processed poultry**	Breaded/battered chicken (incl. fried poultry with batter/breadcrumbs)

Table S2. Definition of the study outcomes

Outcome	ICD-10 code
**Cardiovascular disease**	I20-I25; I60-I69
**Coronary heart disease**	I20-Angina pectoris
	I21-Acute myocardial infarction
	I22-Subsequent myocardial infarction
	I23-Certain current complications following acute myocardial infarction
	I24-Acute ischaemic heart disease
	I25-Chronic ischaemic heart disease
**Cerebrovascular disease**	I60-Subarachnoid haemorrhage
	I61-Intracerebral haemorrhage
	I62-Other nontraumatic intracranial haemorrhage
	I63-Cerebral infarction
	I64-Stroke not specified as haemorrhage or infarction
	I65-Occlusion and stenosis of precerebral arteries, not resulting in cerebral infarction
	I66-Occlusion and stenosis of cerebral arteries, not resulting in cerebral infarction
	I67-Other cerebrovascular diseases
	I68-Cerebrovascular disorders in diseases classified elsewhere
	I69-Sequelae of cerebrovascular disease

Abbreviations. ICD-10: the International Classification of Diseases-Tenth Edition

Table S3. Description of covariates with related UK Biobank field IDs*

Covariate	Description	Field ID
**Sociodemographic factors**		
Age	Participants' age (years) on the day they attended the assessment centre, which were derived from the date of birth and date of attending the assessment centre.	21003
Sex	Participants' sex (female/male)	31
Ethnic background	Information on participants' ethnic background was obtained based on the responses to the questions "What is your ethnic group?" and "What is your ethnic background?", participants were classified into White Europeans (British, Irish, any other White background); South Asians (Asian or Asian British, Indian, Pakistani, Bangladeshi); African Caribbean (Black or Black British, Caribbean, African, any other Black background); Multiple ethnic background or other (Mixed, White and Black Caribbean, White and Black African, White and Asian, any other mixed background, any other Asian background, Chinese, other ethnic group); Unknown/missing ("Do not know", "Prefer not to answer" or missing)	21000
Education	Participants were asked the question "Which of the following qualifications do you have? (You can select more than one)" and were categorised into College or university degree/vocation (College or university degree, National Vocational Qualification (NVQ) or Higher National Diploma (HDN) or Higher National Certificate (HNC) or equivalent, or other professional qualifications); National examination at 17-18 years of age (A levels/AS levels or equivalent); National examination at 16 years of age (O levels/ General Certificate of Secondary Education (GCSEs) or equivalent, or Certificate of Secondary Education (CSEs) or equivalent); Unknown/missing ("None of the above", missing)	6138

Covariate	Description	Field ID
Townsend deprivation index	Participants were categorised intro quintiles, ranging from least deprived (quintile 1) to most deprived (quintile 5) or missing.	22189
Country of assessment centre	The UK Biobank assessment centre at which participants consented. Based on this, participants were categorised into England; Scotland; Wales	54
**Lifestyle/dietary factors**		
Smoking	Participants were categorised into “Never smokers/just tried once or twice”, “Previous smoker”, “Current smoker” and “Unknown/missing”, based on their responses to the touchscreen questions "Do you smoke tobacco now?" and "In the past, how often have you smoked tobacco?"	1239, 1249
Physical activity	Participants were asked on the touchscreen questions on three activity categories: walking, moderate and vigorous physical activity. This information was used to estimate the excess metabolic equivalents (METs) of physical activity in hours per week, as described previously (6). The excess METs represent the energy expenditure above that of an inactive person. Participants were categorised into Low (0-10 excess METs hr/week); Moderate (10-50 excess METs hr/week); High (≥50 excess METs hr/week); Unable to walk; Unknown/missing (“Do not know”, “Prefer not to answer”, missing)	864, 874, 884, 894, 904, 914
Dietary supplement use	Participant who reported use of any dietary supplements were classified as user of dietary supplements. Vitamins, minerals, herbal supplements, and other non-vitamin non-mineral dietary supplements (such as glucosamine and amino acids) were considered as dietary supplements.	6155, 6179, 20003
Alcohol intake	Total alcohol intake was calculated based on responses given by participants to the touchscreen question "About how often do you drink alcohol?". Response categories were “Daily or almost daily”, “Three or four times a week”, “Once or twice a week”, “One to	1558, 4429, 4418, 4451, 4462, 4407, 4440, 1588,

Covariate	Description	Field ID
	three times a month", "Special occasions only" and "Never" or "Prefer not to answer". Participants were subsequently asked to report their average monthly or weekly consumption of alcoholic beverages, including beer and cider, champagne and white wine, fortified wine, other alcohol beverages, red wine and spirits. Total alcohol intake was calculated by multiplying the number of servings of each alcoholic beverage by 10 g of alcohol per serving, except for beer and cider, which was multiplied by 20 g of alcohol per serving. Based on the estimated total alcohol intake, participants were categorised into Non-drinkers (those who reported never drinking alcohol); <1 g/d (including those who reported drinking alcohol on special occasions only); 1-10 g/d; 10-20 g/d; ≥20 g/d; Unknown/missing ("Do not know", "Prefer not to answer", missing)	1578, 1608, 5364, 1568, 1598
Total energy intake	Total energy intake (kcal/d) was calculated by dividing total energy intake (kJ/d) by 4.184. The average energy intake was calculated from all food sources.	26002
Fruit and vegetables intake	Fruit and vegetables intake (g/d) was calculated by summing the intake of raw salad, green leafy vegetables, root vegetables, tomatoes, allium vegetables, other vegetables, peas and corn, citrus fruit, berries, apples and pears and other fruits.	26123, 26098, 26125, 26143, 26065, 26146, 26115, 26091, 26090, 26089, 26093
Fibre intake	The average fibre intake (g/d) was calculated from all food sources.	26017
Protein intake	The average protein intake (en%) was calculated from all food sources.	26005
Carbohydrate intake	The average carbohydrate intake (en%) was calculated from all food sources.	26013
Monounsaturated fatty acids (MUFA) intake	The average monounsaturated fatty acids intake (en%) was calculated from all food sources.	26032
Polyunsaturated fatty acids (PUFA) Intake	The average polyunsaturated fatty acids intake (en%) was calculated from all food sources, by summing n-3 fatty acids and n-6 fatty acids.	26015, 26016

Covariate	Description	Field ID
Saturated fatty acids (SFA) intake from other food sources	The average SFA intake (en%) was calculated from cereal and cereal products, fat spreads (incl. olive oil, dairy-based, plant-based spreads), vegetables and vegetables dishes, sugar, sweet spreads and preserves, eggs and egg products, soups, savoury sauces, samosa, pakora, sushi, fish and fish products, savoury snacks, nuts and seeds	-
Trans-fatty acids	The average trans fatty acids (en%) was calculated from all food sources.	26155
Dietary cholesterol	The average cholesterol intake (mg/d) was calculated from all food sources.	26037
**Female factors**		
Hormone replacement therapy	Female participants were asked to report if they have ever used hormone replacement, with response options; "Yes", "No", "Do not know", "Prefer not to answer".	2814
Menopausal status	Female participants were asked the touchscreen question ""Have you had your menopause (periods stopped)?". Response categories were "Yes", "No", "Not sure – had a hysterectomy", "Not sure – other reason", "Prefer not to answer" during the initial assessment centre visit.	2724
**Family history/cardiometabolic risk factors**		
Family history of CVD	Participants were classified as having family history of CVD based on their responses to the questions on illnesses of the mother, father and siblings (blood relations only). Questions included "Has/did your mother ever suffer from? (You can select more than one answer)", "Has/did your father ever suffer from? (You can select more than one answer)", "Have any of your brothers or sisters suffered from any of the following diseases? (You can select more than one answer)". Response options included "Heart disease", "Stroke", "High blood pressure", "Chronic bronchitis/emphysema", "Alzheimer's disease/dementia", "Diabetes", "Do not know", "Prefer not to answer", "None of the above", "Parkinson's disease",	20110, 20107, 20111

Covariate	Description	Field ID
	“Severe Depression”, “Lung cancer”, “Bowel cancer”, “Breast cancer”. Participants who have selected “Heart disease” or “Stroke” as part of their responses were classified as having family history of CVD.	
Body mass index (BMI)	Participants were categorised into one of the following BMI categories: “Underweight (<18.5 kg/m^2)”, “Healthy weight (18.5-24.99 kg/m^2)”, “Overweight (25-29.99 kg/m^2)”, “Obese (≥30 kg/m^2), “Missing”, based on their calculated BMI as weight (kg)/height (m^2).	23098, 21002, 50
Waist circumference	Data on waist circumference were used to categorise participants into non-abdominal obese (males: <102 cm; females: <88 cm), abdominal obese (males: ≥102 cm; females: ≥88 cm) or missing, using cut-points that were defined previously (7).	48
Baseline hypertension	Baseline hypertension was defined as self-reported hypertension or as use of antihypertensive medication. Antihypertensive medications included angiotensin-converting enzyme (ACE)-inhibitors, angiotensin receptor blockers (ARB), calcium channel blockers (CCB), thiazide-related diuretic (thiazide), alpha-blockers and beta-blockers.	6150, 20002, 20003
Baseline hypercholesterolemia	Baseline hypercholesterolemia was defined as self-reported high cholesterol and reported use of statins from nurse-guided interview.	20002, 20003
Baseline diabetes	Baseline diabetes was defined as self-reported diabetes or diabetes medication. Diabetes medication included insulin, metformin, sulfonylureas, other oral anti-diabetic (OAD) (acarbose, guar, gum), meglitinides, glitazones, non-metformin OADs.	2443, 20002, 6177, 6153, 20003

**Abbreviations. ACE: angiotensin converting enzyme; ARB: angiotensin receptor blockers; BMI: body mass index; CCB: calcium channel blockers; CSE: certificate of secondary education; CVD: cardiovascular diseases; en%: proportion (%) of total energy intake; GCSE: General Certificate of Secondary Education; HDN: higher national diploma; HNC: higher national certificate; METs: metabolic equivalent of tasks; NVQ: national vocational qualifications; OAD: oral anti-diabetic*

Table S4. Cohort characteristics across quartiles of saturated fatty acids intake from total dairy (percentage of total energy intake (%)) among 120,496 study participants in the UK Biobank*

	Quartiles of SFA intake from total dairy (en%)			
**Range**	**0-1.9**	**1.9-3.1**	**3.1-4.6**	**4.6-21.4**
N	30,124	30,124	30,124	30,124
**Sociodemographic factors**				
Age (years)	55.3 ±7.9	55.8 ±7.8	56.2 ±7.8	56.4 ±7.7
Female sex	54.3 (16,361)	56.2 (16,939)	57.7 (17,381)	59.8 (18,008)
White Europeans	94.8 (28,551)	96.7 (29,138)	97.2 (29,269)	97.5 (29,370)
College or university degree/vocation	53.9 (16,231)	55.8 (16,801)	58.4 (17,578)	60.1 (18,098)
Least deprived	20.9 (6,294)	22 (6,616)	22.4 (6,736)	22.2 (6,676)
Country of assessment centre				
England	91.6 (27,606)	91.5 (27,571)	91.7 (27,622)	91.3 (27,499)
Scotland	5.1 (1,523)	5.4 (1,621)	5.3 (1,583)	5.7 (1,703)
Wales	3.3 (995)	3.1 (932)	3.1 (919)	3.1 (922)
**Lifestyle factors**				
Current smoker	7.3 (2,211)	6.9 (2,064)	6.7 (2,017)	6.5 (1,967)
Physical activity (excess METs)				
Low <10	27.9 (8,409)	27.3 (8,221)	27.2 (8,203)	28.7 (8,644)
Moderate 10-50	52.6 (15,856)	53.3 (16,052)	53.8 (16,193)	52.6 (15,856)
High ≥50	17.5 (5,282)	17.4 (5,251)	17 (5,130)	16.6 (4,997)
Dietary supplement use	53.5 (16,128)	52.6 (15,835)	52.3 (15,759)	51.4 (15,488)

	Quartiles of SFA intake from total dairy (en%)			
**Range**	**0-1.9**	**1.9-3.1**	**3.1-4.6**	**4.6-21.4**
Alcohol intake (g/d)				
Non-drinker	6 (1,812)	5.3 (1,601)	5.1 (1,529)	6.1 (1,850)
<1	10.5 (3,152)	10.3 (3,106)	10.1 (3,042)	11.9 (3,588)
1-<10	24 (7,240)	25.9 (7,797)	28.1 (8,468)	29.5 (8,894)
10-<20	23.4 (7,044)	25.3 (7,610)	25.9 (7,810)	24.9 (7,493)
>=20	30.8 (9,275)	27.9 (8,416)	25.4 (7,643)	21.2 (6,383)
**Female factors**				
Hormone replacement therapy use†	18.9 (5,704)	19.3 (5,826)	20 (6,012)	21.2 (6,399)
Menopause status†	30.4 (9,169)	32.5 (9,785)	34.5 (10,394)	36.4 (10,957)
**Family history/cardiometabolic risk factors**				
Family history of CVD	56.6 (17,055)	55.9 (16,847)	56.3 (16,963)	55.9 (16,844)
BMI categories (kg/m^2)				
Underweight <18.5	0.5 (161)	0.5 (152)	0.6 (184)	0.7 (212)
Healthy weight 18.5-25	37.3 (11,244)	39.6 (11,941)	40.5 (12,214)	41.4 (12,470)
Overweight 25-30	41.4 (12,484)	40.6 (12,226)	40.6 (12,221)	39.6 (11,918)
Obesity 30<	20.5 (6,185)	19.1 (5,753)	18 (5,425)	18.1 (5,451)
Abdominal obesity	29 (8,745)	27.7 (8,338)	27.5 (8,275)	27.3 (8,233)
Baseline hypertension	25.6 (7,717)	24.6 (7,399)	23.5 (7,078)	22.8 (6,869)
Baseline hypercholesterolemia	14.4 (4,343)	13.3 (4,019)	12.5 (3,760)	11.5 (3,461)
Baseline diabetes mellitus	2.6 (795)	2.4 (713)	2.1 (647)	2 (605)
**Dietary intake**				
Total energy intake (kcal/d)	1,992 ±500	2,070 ±499	2,091 ±491	2,077 ±495

	Quartiles of SFA intake from total dairy (en%)			
**Range**	**0-1.9**	**1.9-3.1**	**3.1-4.6**	**4.6-21.4**
Total protein (en%)	16.1 ±3.4	15.9 ±3	15.8 ±2.8	15.7 ±2.8
Total carbohydrate (en%)	50.1 ±8	49.7 ±7.3	49.1 ±7	48 ±6.9
Total fat (en%)	29.7 ±6	31 ±5.5	32.2 ±5.3	34.1 ±5.3
Total MUFA (en%)	11.4 ±2.7	11.4 ±2.4	11.5 ±2.3	11.6 ±2.3
Total PUFA (en%)	6 ±1.7	5.7 ±1.5	5.6 ±1.5	5.3 ±1.5
Total SFA (en%)	9.7 ±2.6	11 ±2.4	12.2 ±2.3	14 ±2.5
SFA from total dairy (en%)	1.1 ±0.5	2.5 ±0.4	3.8 ±0.4	6.1 ±1.5
SFA from total meat (en%)	1.8 ±1.4	1.7 ±1.2	1.6 ±1.2	1.4 ±1.2
SFA from cereal and cereal products (en%)	2.7 ±1.7	2.8 ±1.6	2.8 ±1.6	2.6 ±1.5
SFA from fat spreads (en%)	1.1 ±1.4	1.2 ±1.4	1.3 ±1.4	1.3 ±1.5
SFA from vegetables and vegetables dishes (en%)	0.8 ±0.7	0.7 ±0.7	0.7 ±0.7	0.7 ±0.7
SFA from sugar, sweet spreads, preserves (en%)	0.6 ±1	0.6 ±0.9	0.5 ±0.8	0.5 ±0.8
SFA from eggs and egg products (en%)	0.4 ±0.7	0.4 ±0.6	0.4 ±0.6	0.3 ±0.6
SFA from soups, savoury sauces, samosa, pakora, sushi (en%)	0.4 ±0.4	0.4 ±0.4	0.4 ±0.3	0.4 ±0.3
SFA from fish and fish products (en%)	0.3 ±0.5	0.3 ±0.4	0.2 ±0.4	0.2 ±0.4
SFA from savoury snacks (en%)	0.2 ±0.3	0.2 ±0.3	0.1 ±0.3	0.1 ±0.2
SFA from nuts and seeds (en%)	0.3 ±0.5	0.3 ±0.4	0.2 ±0.4	0.2 ±0.4
Total TFA (en%)	0.4 ±0.2	0.5 ±0.2	0.5 ±0.2	0.7 ±0.2
Fibre (g/d)	18.1 ±6.3	18.2 ±5.8	18 ±5.6	17.2 ±5.4

Range	Quartiles of SFA intake from total dairy (en%)			
	**0-1.9**	**1.9-3.1**	**3.1-4.6**	**4.6-21.4**
Sodium (mg/d)	1,884 ±673	1,962 ±662	1,981 ±645	1,982 ±646
Dietary cholesterol (mg/d)	238 ±142	246 ±132	248 ±129	252 ±128
Fruit and vegetables (g/d)	399.4 ±249	389.3 ±228.1	380.8 ±214.2	365.6 ±207.8
Total dairy (g/d)	227.6 ±143.2	303.5 ±151	341 ±158.1	393 ±174.5
Total meat (g/d)	100.7 ±67	94.1 ±60.1	89.5 ±58.1	80.6 ±57.3

**Data are presented as means ±SD for continuous variables or as % (n) for categorical variables, with % representing column percentages. Abbreviations. BMI: body mass index; CVD: cardiovascular diseases; en%: proportion (%) of total energy intake; METs, Metabolic equivalent of tasks; MUFA: monounsaturated fatty acids; PUFA: polyunsaturated fatty acids; SFA: saturated fatty acids; TFA: trans unsaturated fatty acids*

†Females only

Table S5. Cohort characteristics across quartiles of saturated fatty acids intake from milk (percentage of total energy intake (%)) among 120,496 study participants in the UK Biobank*

	Quartiles of SFA intake from milk (en%)			
**Range**	**0.0-0.3**	**0.3-0.8**	**0.8-1.2**	**1.2-11.0**
N	30,124	30,124	30,124	30,124
**Sociodemographic factors**				
Female sex	62.9 (18,952)	54.1 (16,298)	53.8 (16,211)	57.2 (17,228)
Age, years	55.7 ±7.8	55.6 ±7.9	55.9 ±7.8	56.4 ±7.8
White Europeans	96.2 (28,990)	95.9 (28,900)	97 (29,228)	97 (29,210)
College or university degree/vocation	57.6 (17,359)	57.8 (17,425)	57.4 (17,304)	55.2 (16,620)
Least deprived	21.1 (6,371)	21.5 (6,464)	22.1 (6,670)	22.6 (6,817)
Country of assessment centre				
England	91.7 (27,621)	90.9 (27,392)	91.8 (27,650)	91.7 (27,635)
Scotland	4.9 (1,483)	5.9 (1,779)	5.2 (1,573)	91.7 (1,595)
Wales	3.4 (1,020)	3.2 (953)	3 (901)	3 (894)
**Lifestyle factors**				
Current smoker	6.1 (1,849)	6.9 (2,076)	6.5 (1,962)	7.9 (2,372)
Physical activity (excess METs)				
Low <10	27 (8,146)	27.6 (8,312)	28 (8,437)	28.5 (8,582)
Moderate 10-50	53.7 (16,165)	53.5 (16,124)	53.3 (16,060)	51.8 (15,608)
High ≥50	17.4 (5,255)	17 (5,113)	16.7 (5,016)	17.5 (5,276)
Dietary supplement use	56.1 (16,898)	51.9 (15,626)	50.4 (15,196)	51.4 (15,490)
Alcohol intake (g/d)				

	Quartiles of SFA intake from milk (en%)			
**Range**	**0.0-0.3**	**0.3-0.8**	**0.8-1.2**	**1.2-11.0**
Non-drinker	6.1 (1,843)	5 (1,519)	4.5 (1,370)	6.8 (2,060)
<1	10.8 (3,263)	9.6 (2,888)	9.4 (2,845)	12.9 (3,892)
1-<10	26 (7,846)	24.7 (7,451)	27 (8,143)	29.7 (8,959)
10-<20	24.5 (7,390)	24.5 (7,369)	26.5 (7,981)	24 (7,217)
>=20	26.9 (8,110)	31.2 (9,400)	27.4 (8,243)	19.8 (5,964)
**Female factors**				
Hormone replacement therapy†	22.6 (6,819)	18.4 (5,535)	18.2 (5,495)	20.2 (6,092)
Menopausal status†	36.2 (10,915)	31 (9,339)	31.6 (9,510)	35 (10,541)
**Family history/cardiometabolic risk factors**				
Family history of CVD	57.3 (17,267)	56 (16,858)	55.7 (16,772)	55.8 (16,812)
BMI categories (kg/m^2)				
Underweight <18.5	0.6 (184)	0.5 (143)	0.5 (165)	0.7 (217)
Healthy weight 18.5-25	38.9 (11,725)	37.4 (11,257)	40.2 (12,112)	42.4 (12,775)
Overweight 25-30	39.7 (11,968)	41.5 (12,512)	41.2 (12,410)	39.7 (11,959)
Obesity 30<	20.5 (6,187)	20.4 (6,154)	17.8 (5,369)	16.9 (5,104)
Abdominal obesity	29.8 (8967)	29.6 (8926)	26.8 (8060)	25.4 (7638)
Baseline hypertension	24.6 (7,400)	25.1 (7,570)	23.8 (7,176)	23 (6,917)
Baseline hypercholesterolemia	13.5 (4,052)	13.5 (4,076)	12.6 (3,782)	12.2 (3,673)
Baseline diabetes mellitus	2.2 (649)	2.3 (706)	2.3 (699)	2.3 (706)
**Dietary intake**				
Total energy intake (kcal/d)	2,002 ±494	2,147 ±524	2,110 ±475	1,970 ±475
Total protein (en%)	16 ±3.3	15.6 ±3	15.7 ±2.8	16.2 ±2.9

	Quartiles of SFA intake from milk (en%)			
**Range**	**0.0-0.3**	**0.3-0.8**	**0.8-1.2**	**1.2-11.0**
Total carbohydrate (en%)	49.1 ±8.1	48.4 ±7.5	49.1 ±6.9	50.3 ±6.8
Total fat (en%)	31.2 ±6.2	32 ±5.9	32.1 ±5.5	31.7 ±5.5
Total MUFA (en%)	11.5 ±2.7	11.7 ±2.5	11.6 ±2.3	11.2 ±2.3
Total PUFA (en%)	6 ±1.7	5.7 ±1.5	5.6 ±1.5	5.3 ±1.4
Total SFA (en%)	10.9 ±3	11.7 ±2.9	12 ±2.8	12.3 ±2.8
SFA from total dairy (en%)	1.1 ±0.5	2.5 ±0.4	3.8 ±0.4	6.1 ±1.5
SFA from milk (en%)	0.1 ±0.1	0.5 ±0.1	1.0 ±0.1	1.8 ±0.7
SFA from yogurt (en%)	0.2 ±0.4	0.2 ±0.4	0.2 ±0.4	0.3 ±0.4
SFA from cheese (en%)	1.4 ±1.5	1.4 ±1.4	1.4 ±1.3	1.3 ±1.4
SFA from total meat (en%)	1.6 ±1.3	1.7 ±1.3	1.6 ±1.2	1.6 ±1.2
SFA from unprocessed meat (en%)	0.6 ±0.6	0.7 ±0.6	0.7 ±0.6	0.6 ±0.6
SFA from processed meat (en%)	0.4 ±0.7	0.5 ±0.7	0.5 ±0.7	0.4 ±0.7
SFA from red meat (en%)	0.7 ±0.8	0.8 ±0.8	0.8 ±0.7	0.7 ±0.7
SFA from unprocessed red meat (en%)	0.8 ±1	0.9 ±1	0.9 ±1	0.8 ±1
SFA from processed red meat (en%)	0.4 ±0.7	0.5 ±0.7	0.4 ±0.7	0.4 ±0.7
SFA from poultry (en%)	0.3 ±0.4	0.3 ±0.4	0.3 ±0.3	0.3 ±0.4
SFA from unprocessed poultry (en%)	0.3 ±0.4	0.2 ±0.3	0.2 ±0.3	0.2 ±0.3
SFA from processed poultry (en%)	0.0 ±0.1	0.0 ±0.1	0.0 ±0.1	0.0 ±0.1
SFA from cereal and cereal products (en%)	2.7 ±1.7	2.8 ±1.6	2.8 ±1.6	2.6 ±1.5
SFA from fat spreads (en%)	1.0 ±1.3	1.3 ±1.5	1.3 ±1.4	1.3 ±1.4

Range	Quartiles of SFA intake from milk (en%)			
	**0.0-0.3**	**0.3-0.8**	**0.8-1.2**	**1.2-11.0**
SFA from vegetables and vegetables dishes (en%)	0.8 ±0.7	0.7 ±0.7	0.7 ±0.7	0.7 ±0.7
SFA from sugar, sweet spreads, preserves (en%)	0.6 ±0.9	0.6 ±0.9	0.5 ±0.8	0.5 ±0.8
SFA from eggs and egg products (en%)	0.4 ±0.7	0.4 ±0.6	0.4 ±0.6	0.3 ±0.6
SFA from soups, savoury sauces, samosa, pakora, sushi (en%)	0.4 ±0.4	0.4 ±0.4	0.4 ±0.3	0.4 ±0.3
SFA from fish and fish products (en%)	0.3 ±0.4	0.3 ±0.4	0.2 ±0.4	0.2 ±0.4
SFA from savoury snacks (en%)	0.2 ±0.3	0.2 ±0.3	0.2 ±0.3	0.1 ±0.2
SFA from nuts and seeds (en%)	0.3 ±0.5	0.3 ±0.5	0.2 ±0.4	0.2 ±0.4
Total TFA (en%)	0.5 ±0.2	0.5 ±0.2	0.5 ±0.2	0.6 ±0.2
Fibre (g/d)	18.6 ±6.3	18.2 ±5.9	17.9 ±5.5	16.9 ±5.4
Sodium (mg/d)	1,923 ±669	2,055 ±700	1,991 ±634	1,842 ±605
Dietary cholesterol (mg/d)	239 ±143	258 ±138	250 ±126	236 ±123
Fruit and vegetables (g/d)	427.2 ±249.6	387.7 ±229.1	370.6 ±211.9	349.6 ±202
Total dairy (g/d)	212.1 ±144.1	274.4 ±138.9	334.8 ±121.3	443.8 ±171.1
Milk (g/d)	124.8 ±117.7	186.5 ±112.5	247.8 ±91.4	361 ±149.8
Yogurt (g/d)	46.5 ±57.3	42.9 ±52.8	42.6 ±51.1	43 ±51.4
Cheese (g/d)	17.9 ±18.9	18.9 ±19.1	17.8 ±17.5	16 ±16.6
Total meat (g/d)	89.2 ±65.1	97 ±64.4	93.7 ±58.7	84.9 ±55.3
Unprocessed meat (g/d)	66.9 ±55.7	71.4 ±55	69.3 ±51.4	63.5 ±49

	Quartiles of SFA intake from milk (en%)			
**Range**	**0.0-0.3**	**0.3-0.8**	**0.8-1.2**	**1.2-11.0**
Processed meat (g/d)	20.2 ±28.2	23.4 ±29.7	22.2 ±27.7	19.6 ±25.7
Red meat (g/d)	52 ±50.2	59.3 ±51.6	57.6 ±48.1	52.2 ±45.1
Unprocessed red meat (g/d)	34.8 ±41.7	39.4 ±43.1	38.7 ±41	35.6 ±39
Processed red meat (g/d)	17.2 ±24.8	19.9 ±26.2	18.9 ±24.5	16.6 ±22.7
Poultry (g/d)	35.1 ±43.4	35.4 ±42.7	33.9 ±40.2	30.9 ±38.5
Unprocessed poultry (g/d)	32.1 ±41.6	32 ±40.8	30.6 ±38.6	27.9 ±36.8
Processed poultry (g/d)	3 ±12.8	3.4 ±13.7	3.3 ±12.8	3.1 ±12.4

**Data are presented as means ±SD for continuous variables or as % (n) for categorical variables, with % representing column percentages. Abbreviations. BMI: body mass index; CVD: cardiovascular diseases; en%: proportion (%) of total energy intake; METs, Metabolic equivalent of tasks; MUFA: monounsaturated fatty acids; PUFA: polyunsaturated fatty acids; SFA: saturated fatty acids; TFA: trans unsaturated fatty acids*

†*Females only*

Table S6. Cohort characteristics in zero consumers and consumers of saturated fatty acids intake from yogurt (percentage of total energy intake (%)) among 120,496 study participants in the UK Biobank*

	SFA from yogurt (en%)	
**Range**	**Zero consumers**	**Consumers (0.0-5.4)**
N	53,851	66,645
**Sociodemographic factors**		
Female sex	49.2 (26,489)	63.3 (42,200)
Age, years	55.4 ±8	56.4 ±7.7
White Europeans	96 (51,683)	97 (64,645)
College or university degree/vocation	56.5 (30,407)	57.5 (38,301)
Least deprived	21.1 (11,363)	22.4 (14,959)
Country of assessment centre		
England	91.2 (49,116)	91.8 (61,182)
Scotland	5.4 (2,920)	5.3 (3,510)
Wales	3.4 (1,815)	2.9 (1,953)
**Lifestyle factors**		
Current smoker	8.9 (4,817)	5.2 (3,442)
Physical activity (excess METs)		
Low <10	29.7 (16,004)	26.2 (17,473)
Moderate 10-50	51.6 (27,783)	54.3 (36,174)
High ≥50	16.7 (8,982)	17.5 (11,678)
Dietary supplement use	47.9 (25,813)	56.1 (37,397)
Alcohol intake (g/d)		
Non-drinker	5.5 (2,974)	5.7 (3,818)

	SFA from yogurt (en%)	
Range	Zero consumers	Consumers (0.0-5.4)
<1	10.1 (5,440)	11.2 (7,448)
1-<10	23.9 (12,895)	29.3 (19,504)
10-<20	23.8 (12,798)	25.7 (17,159)
>=20	31.2 (16,817)	22.4 (14,900)
**Female factors**		
Hormone replacement therapy†	15.9 (8,541)	23.1 (15,400)
Menopausal status†	27 (14,548)	38.6 (25,757)
**Family history/cardiometabolic risk factors**		
Family history of CVD	54.7 (29,440)	57.4 (38,269)
BMI categories (kg/m^2)		
Underweight <18.5	0.6 (312)	0.6 (397)
Healthy weight 18.5-25	37.9 (20,384)	41.2 (27,485)
Overweight 25-30	41.1 (22,137)	40.1 (26,712)
Obesity 30<	20.2 (10,898)	17.9 (11,916)
Abdominal obesity	28.7 (15,426)	27.3 (18,165)
Baseline hypertension	25.2 (13,578)	23.2 (15,485)
Baseline hypercholesterolemia	13.8 (7,454)	12.2 (8,129)
Baseline diabetes mellitus	2.9 (1,541)	1.8 (1,219)
**Dietary intake**		
Total energy intake (kcal/d)	2,065 ±516	2,051 ±482
Total protein (en%)	15.6 ±3	16.1 ±3
Total carbohydrate (en%)	48.1 ±7.6	50.1 ±7
Total fat (en%)	32.3 ±5.9	31.3 ±5.6

Range	SFA from yogurt (en%)	
	Zero consumers	Consumers (0.0-5.4)
Total MUFA (en%)	11.8 ±2.5	11.3 ±2.4
Total PUFA (en%)	5.7 ±1.6	5.6 ±1.5
Total SFA (en%)	12 ±3	11.5 ±2.8
SFA from total dairy (en%)	3.2 ±2.1	3.5 ±2
SFA from milk (en%)	0.9 ±0.8	0.9 ±0.7
SFA from yogurt (en%)	0 ±0	0.4 ±0.4
SFA from cheese (en%)	1.4 ±1.5	1.4 ±1.3
SFA from total meat (en%)	1.8 ±1.4	1.5 ±1.2
SFA from unprocessed meat (en%)	0.7 ±0.6	0.6 ±0.6
SFA from processed meat (en%)	0.5 ±0.8	0.4 ±0.6
SFA from red meat (en%)	0.8 ±0.8	0.7 ±0.7
SFA from unprocessed red meat (en%)	0.9 ±1.1	0.8 ±0.9
SFA from processed red meat (en%)	0.5 ±0.7	0.4 ±0.6
SFA from poultry (en%)	0.3 ±0.4	0.3 ±0.3
SFA from unprocessed poultry (en%)	0.3 ±0.4	0.2 ±0.3
SFA from processed poultry (en%)	0 ±0.1	0 ±0.1
SFA from cereal and cereal products (en%)	2.8 ±1.7	2.7 ±1.5
SFA from fat spreads (en%)	1.4 ±1.5	1.1 ±1.3
SFA from vegetables and vegetables dishes (en%)	0.7 ±0.7	0.7 ±0.7
SFA from sugar, sweet spreads, preserves (en%)	0.6 ±0.9	0.5 ±0.8
SFA from eggs and egg products (en%)	0.4 ±0.7	0.4 ±0.6
SFA from soups, savoury sauces, samosa, pakora, sushi (en%)	0.4 ±0.4	0.4 ±0.4

	SFA from yogurt (en%)	
Range	Zero consumers	Consumers (0.0-5.4)
SFA from fish and fish products (en%)	0.3 ±0.4	0.3 ±0.4
SFA from savoury snacks (en%)	0.2 ±0.3	0.1 ±0.2
SFA from nuts and seeds (en%)	0.2 ±0.5	0.2 ±0.4
Total TFA (en%)	0.5 ±0.2	0.5 ±0.2
Fibre (g/d)	17 ±5.7	18.6 ±5.8
Sodium (mg/d)	1,983 ±690	1,928 ±630
Fruit and vegetables (g/d)	339.8 ±211.9	419.3 ±230.2
Total dairy (g/d)	261.2 ±154.7	360.8 ±165.6
Milk (g/d)	217.8 ±145.2	239.9 ±149.8
Yogurt (g/d)	0 ±0.0	79.1 ±48.2
Cheese (g/d)	17.3 ±18.6	17.9 ±17.6
Total meat (g/d)	97 ±63.9	86.6 ±58.5
Unprocessed meat (g/d)	71 ±55.1	65.1 ±50.9
Processed meat (g/d)	23.6 ±30	19.5 ±25.9
Red meat (g/d)	60.2 ±51.9	51.3 ±46
Unprocessed red meat (g/d)	40.3 ±43.9	34.6 ±38.9
Processed red meat (g/d)	19.9 ±26.5	16.7 ±22.9
Poultry (g/d)	34.4 ±42.7	33.4 ±40.1
Unprocessed poultry (g/d)	30.7 ±40.6	30.5 ±38.6
Processed poultry (g/d)	3.6 ±14.1	2.8 ±11.8

**Data are presented as means ±SD for continuous variables or as % (n) for categorical variables, with % representing column percentages. Abbreviations. BMI: body mass index; CVD: cardiovascular diseases; en%: proportion (%) of total energy intake; METs, Metabolic equivalent of tasks; MUFA: monounsaturated fatty acids; PUFA: polyunsaturated fatty acids; SFA: saturated fatty acids; TFA: trans unsaturated fatty acids*

†Females only

Table S7. Cohort characteristics across quartiles of saturated fatty acids intake from cheese (percentage of total energy intake (%)) among 120,496 study participants in the UK Biobank*

	Quartiles of SFA intake from cheese (en%)			
**Range**	**0.0-0.0**	**0.0-1.1**	**1.1-2.1**	**2.1-21.4**
N	32,166	28,082	30,124	30,124
**Sociodemographic factors**				
Female sex	54.5 (17,522)	58 (16,293)	57.8 (17,409)	58.0 (17,465)
Age, years	55.7 ±7.8	56.1 ±7.8	56.1 ±7.8	55.8 ±7.8
White Europeans	94.7 (30,466)	96.9 (27,199)	97.3 (29,307)	97.5 (29,356)
College or university degree/vocation	51.1 (16,442)	56.1 (15,751)	58.6 (17,643)	62.6 (18,872)
Least deprived	21.7 (6,969)	22.1 (6,208)	22.4 (6,733)	21.3 (6,412)
Country of assessment centre				
England	91.3 (29,383)	91.9 (25,820)	91.8 (27,664)	91.1 (27,431)
Scotland	5.2 (1,678)	5.1 (1,443)	5.1 (1,540)	5.9 (1,769)
Wales	3.4 (1,105)	2.9 (819)	3.1 (920)	3.1 (924)
**Lifestyle factors**				
Current smoker	7.2 (2,303)	6.4 (1,788)	6.5 (1,968)	7.3 (2,200)
Physical activity (excess METs)				
Low <10	29.1 (9,367)	27.1 (7,620)	27.2 (8,189)	27.6 (8,301)
Moderate 10-50	51.3 (16,487)	53.4 (14,991)	54 (16,267)	53.8 (16,212)
High ≥50	17.4 (5,590)	17.6 (4,938)	16.9 (5,103)	16.7 (5,029)
Dietary supplements use	52.2 (16,778)	54.2 (15,214)	52.7 (15,876)	50.9 (15,342)
Alcohol intake (g/d)				
Non-drinker	7.2 (2,314)	5.3 (1,492)	4.9 (1,489)	5 (1,497)

	Quartiles of SFA intake from cheese (en%)			
**Range**	**0.0-0.0**	**0.0-1.1**	**1.1-2.1**	**2.1-21.4**
<1	12.1 (3,887)	11.2 (3,142)	9.6 (2,894)	9.8 (2,965)
1-<10	25.7 (8,267)	27.3 (7,655)	27.7 (8,338)	27 (8,139)
10-<20	23.1 (7,419)	24.8 (6,961)	25.9 (7,795)	25.8 (7,782)
>=20	25.7 (8,274)	26 (7,308)	26.6 (8,014)	27 (8,121)
**Female factors**				
Hormone replacement therapy†	19.7 (6,341)	20.5 (5,759)	20.2 (6,097)	19.1 (5,744)
Menopausal status†	31.4 (10,100)	34.1 (9,578)	34.2 (10,314)	34.2 (10,313)
**Family history/cardiometabolic risk factors**				
Family history of CVD	56.2 (18,068)	57 (16,011)	56.4 (16,997)	55.2 (16,633)
BMI categories (kg/m^2)				
Underweight <18.5	0.5 (145)	0.6 (162)	0.6 (181)	0.7 (221)
Healthy weight 18.5-25	36.7 (11,792)	39.6 (11,114)	40.5 (12,188)	42.4 (12,775)
Overweight 25-30	42 (13,511)	40.7 (11,432)	40.4 (12,170)	39 (11,736)
Obesity 30<	20.7 (6,659)	19 (5,326)	18.3 (5,515)	17.6 (5,314)
Abdominal obesity	29.2 (9388)	27.9 (7834)	27.7 (8343)	26.6 (8026)
Baseline hypertension	25.2 (8,104)	24.6 (6,910)	23.8 (7,179)	22.8 (6,870)
Baseline hypercholesterolemia	14.6 (4,698)	13.3 (3,732)	23.8 (3,799)	11.1 (3,354)
Baseline diabetes mellitus	2.7 (868)	2.2 (613)	2.1 (625)	2.2 (654)
**Dietary intake**				
Total energy intake (kcal/d)	1,952 ±493	2,106 ±479	2,105 ±493	2,077 ±507
Total protein (en%)	16.2 ±3.4	15.8 ±2.9	15.7 ±2.8	15.8 ±3
Total carbohydrate (en%)	50.7 ±7.7	50 ±7	48.9 ±6.9	47.3 ±7.3

	Quartiles of SFA intake from cheese (en%)			
**Range**	**0.0-0.0**	**0.0-1.1**	**1.1-2.1**	**2.1-21.4**
Total fat (en%)	30.2 ±6	31.1 ±5.5	32.1 ±5.4	33.7 ±5.7
Total MUFA (en%)	11.3 ±2.6	11.4 ±2.3	11.6 ±2.3	11.7 ±2.4
Total PUFA (en%)	5.7 ±1.7	5.7 ±1.5	5.6 ±1.5	5.5 ±1.5
Total SFA (en%)	10.5 ±2.9	11.2 ±2.6	12.0 ±2.6	13.3 ±2.8
SFA from total dairy (en%)	2 ±1.6	2.7 ±1.5	3.6 ±1.5	5.2 ±1.8
SFA from milk (en%)	0.9 ±0.8	0.9 ±0.7	0.9 ±0.7	0.9 ±0.7
SFA from yogurt (en%)	0.2 ±0.4	0.2 ±0.3	0.2 ±0.4	0.3 ±0.4
SFA from cheese (en%)	0 ±0.0	0.7 ±0.3	1.6 ±0.3	3.3 ±1.2
SFA from total meat (en%)	1.8 ±1.4	1.6 ±1.2	1.6 ±1.2	1.4 ±1.2
SFA from unprocessed meat (en%)	0.7 ±0.6	0.7 ±0.6	0.6 ±0.6	0.5 ±0.6
SFA from processed meat (en%)	0.5 ±0.8	0.5 ±0.6	0.5 ±0.7	0.4 ±0.7
SFA from red meat (en%)	0.8 ±0.8	0.8 ±0.8	0.8 ±0.7	0.7 ±0.7
SFA from unprocessed red meat (en%)	0.9 ±1.1	0.8 ±0.9	0.8 ±1.0	0.7 ±1.0
SFA from processed red meat (en%)	0.5 ±0.8	0.4 ±0.6	0.4 ±0.7	0.4 ±0.7
SFA from poultry (en%)	0.3 ±0.4	0.3 ±0.3	0.3 ±0.3	0.2 ±0.3
SFA from unprocessed poultry (en%)	0.3 ±0.4	0.3 ±0.3	0.2 ±0.3	0.2 ±0.3
SFA from processed poultry (en%)	0.0 ±0.2	0.0 ±0.1	0.0 ±0.1	0.0 ±0.1
SFA from cereal and cereal products (en%)	2.7 ±1.7	2.9 ±1.6	2.8 ±1.6	2.7 ±1.5
SFA from fat spreads (en%)	1.1 ±1.4	1.1 ±1.3	1.3 ±1.4	1.4 ±1.5
SFA from vegetables and vegetables dishes (en%)	0.7 ±0.7	0.7 ±0.7	0.7 ±0.7	0.7 ±0.7

Range	Quartiles of SFA intake from cheese (en%)			
	**0.0-0.0**	**0.0-1.1**	**1.1-2.1**	**2.1-21.4**
SFA from sugar, sweet spreads, preserves (en%)	0.6 ±1.0	0.6 ±0.8	0.5 ±0.8	0.5 ±0.8
SFA from eggs and egg products (en%)	0.4 ±0.7	0.4 ±0.6	0.4 ±0.6	0.4 ±0.7
SFA from soups, savoury sauces, samosa, pakora, sushi (en%)	0.4 ±0.4	0.4 ±0.3	0.4 ±0.3	0.4 ±0.3
SFA from fish and fish products (en%)	0.3 ±0.5	0.3 ±0.4	0.2 ±0.4	0.2 ±0.4
SFA from savoury snacks (en%)	0.2 ±0.3	0.2 ±0.3	0.2 ±0.3	0.1 ±0.3
SFA from nuts and seeds (en%)	0.2 ±0.5	0.3 ±0.4	0.2 ±0.4	0.2 ±0.4
Total TFA (en%)	0.4 ±0.2	0.5 ±0.2	0.5 ±0.2	0.6 ±0.2
Fibre (g/d)	17.2 ±6	18.5 ±5.7	18.1 ±5.7	17.8 ±5.7
Sodium (mg/d)	1,791 ±641	1,965 ±629	2,006 ±653	2,061 ±675
Dietary cholesterol (mg/d)	237 ±136	249 ±130	250 ±131	249 ±134
Fruit and vegetables (g/d)	374.1 ±236.8	397.3 ±227.5	384 ±217.7	381.3 ±218.9
Total dairy (g/d)	294.4 ±173.6	324.5 ±168.2	324.6 ±166.1	323.8 ±162.5
Milk (g/d)	228 ±153.3	239.4 ±148.4	234.1 ±146.4	219.4 ±143.3
Yogurt (g/d)	41.9 ±54.4	47.1 ±54.2	44.2 ±51.5	42.3 ±52.6
Cheese (g/d)	0 ±0	10.9 ±7	20.5 ±8.4	39.9 ±17.6
Total meat (g/d)	101.2 ±64.4	96.1 ±59.3	89.5 ±58.7	77.8 ±59.3
Unprocessed meat (g/d)	76.3 ±56.7	71.6 ±51.2	66.2 ±50.6	56.7 ±50.5
Processed meat (g/d)	22.8 ±30.3	22 ±27	21.3 ±27.2	19.2 ±26.6
Red meat (g/d)	59.1 ±52.2	57.5 ±47.7	55.5 ±47.9	48.9 ±46.8
Unprocessed red meat (g/d)	40.3 ±44.7	38.8 ±40.4	37.1 ±40.1	32.2 ±39
Processed red meat (g/d)	18.8 ±26.1	18.7 ±23.9	18.4 ±24.4	16.7 ±23.8

	Quartiles of SFA intake from cheese (en%)			
**Range**	**0.0-0.0**	**0.0-1.1**	**1.1-2.1**	**2.1-21.4**
Poultry (g/d)	40 ±46.4	36.1 ±40.5	31.9 ±38.7	27 ±37.4
Unprocessed poultry (g/d)	36 ±44.3	32.8 ±38.9	29.1 ±37.3	24.5 ±35.8
Processed poultry (g/d)	4 ±15.4	3.3 ±12.4	2.9 ±11.9	2.5 ±11.4

**Data are presented as means ±SD for continuous variables or as % (n) for categorical variables, with % representing column percentages. Abbreviations. BMI: body mass index; CVD: cardiovascular diseases; en%: proportion (%) of total energy intake; METs, Metabolic equivalent of tasks; MUFA: monounsaturated fatty acids; PUFA: polyunsaturated fatty acids; SFA: saturated fatty acids; TFA: trans unsaturated fatty acids*

†Females only

Table S8. Cohort characteristics across quartiles of saturated fatty acids intake from total meat (percentage of total energy intake (%)) among 120,496 study participants in the UK Biobank*

	Quartiles of SFA intake from total meat (en%)			
**Range**	**0.0-0.7**	**0.7-1.4**	**1.4-2.3**	**2.3-17.9**
N	30,124	30,124	30,124	30,124
**Sociodemographic factors**				
Age (years)	55.5 ±7.9	56.2 ±7.8	56.1 ±7.8	55.8 ±7.8
Female sex	63.5 (19,119)	58.4 (17,589)	55.5 (16,731)	50.6 (15,250)
White Europeans	95.9 (28,886)	96.9 (29,189)	97.2 (29,271)	96.2 (28,982)
College or university degree/vocation	61.9 (18,656)	56.5 (17,011)	55.3 (16,650)	54.4 (16,391)
Least deprived	20 (6,014)	22.8 (6,875)	22.9 (6,899)	21.7 (6,534)
Country of assessment centre				
England	91.1 (27,445)	91.3 (27,502)	91.7 (27,635)	92 (27,716)
Scotland	5.8 (1,745)	5.6 (1,695)	5.2 (1,571)	4.7 (1,419)
Wales	3.1 (934)	3.1 (927)	3 (918)	3.3 (989)
**Lifestyle factors**				
Current smoker	5.9 (1,775)	6.2 (1,853)	6.8 (2,053)	8.6 (2,578)
Physical activity (excess METs)				
Low <10	24.9 (7,488)	26.7 (8,038)	28.5 (8,572)	31.1 (9,379)
Moderate 10-50	54.6 (16,441)	53.9 (16,235)	53.1 (16,007)	50.7 (15,274)
High ≥50	18.6 (5,614)	17.5 (5,257)	16.4 (4,940)	16.1 (4,849)
Dietary supplement user	55.6 (16,764)	54 (16,258)	51.7 (15,587)	48.5 (14,601)
Alcohol intake (g/d)				
Non-drinker	7.2 (2,182)	5.4 (1,632)	5.1 (1,534)	4.8 (1,444)

	Quartiles of SFA intake from total meat (en%)			
**Range**	**0.0-0.7**	**0.7-1.4**	**1.4-2.3**	**2.3-17.9**
<1	12.5 (3,752)	10.8 (3,259)	10 (3,018)	9.5 (2,859)
1-<10	28.8 (8,687)	28.1 (8,478)	26.5 (7,973)	24.1 (7,261)
10-<20	24.4 (7,358)	25.7 (7,748)	25 (7,534)	24.3 (7,317)
>=20	21 (6,314)	24.4 (7,350)	27.9 (8,405)	32 (9,648)
**Female factors**				
Hormone replacement therapy use	20.3 (6,115)	21.2 (6,384)	19.9 (6,002)	18.1 (5,440)
Menopausal status	37.4 (11,261)	34.6 (10,413)	32.7 (9,863)	29.1 (8,768)
**Family history/cardiometabolic risk factors**				
Family history of CVD	55.9 (16,852)	57.1 (17,202)	56.6 (17,046)	55.1 (16,609)
BMI categories (kg/m^2)				
Underweight <18.5	1 (309)	0.6 (173)	0.4 (125)	0.3 (102)
Healthy weight 18.5-25	49.1 (14,802)	41.2 (12,397)	37 (11,155)	31.6 (9,515)
Overweight 25-30	35.9 (10,818)	40.8 (12,301)	42.2 (12,706)	43.2 (13,024)
Obesity 30<	13.7 (4,123)	17.2 (5,196)	20.2 (6,083)	24.6 (7,412)
Abdominal obesity	21.9 (6,602)	26.2 (7,878)	29.7 (8,942)	33.8 (10,169)
Baseline hypertension	21.1 (6,350)	23.7 (7,154)	25.2 (7,600)	26.4 (7,959)
Baseline hypercholesterolemia	10.4 (3,146)	12.5 (3,770)	13.9 (4,176)	14.9 (4,491)
Baseline diabetes mellitus	1.6 (483)	2 (604)	2.5 (753)	3.1 (920)
**Dietary intake**				
Total energy intake (kcal/d)	2,017 ±495	2,097 ±496	2,081 ±486	2,035 ±509
Total protein (en%)	14.2 ±2.6	15.6 ±2.6	16.2 ±2.7	17.5 ±3.2
Total carbohydrate (en%)	52.5 ±7.1	50.5 ±6.5	48.7 ±6.6	45.3 ±7.2

	Quartiles of SFA intake from total meat (en%)			
**Range**	**0.0-0.7**	**0.7-1.4**	**1.4-2.3**	**2.3-17.9**
Total fat (en%)	31.2 ±6.1	31.1 ±5.6	31.6 ±5.5	33.1 ±5.7
Total MUFA (en%)	11.2 ±2.6	11.2 ±2.3	11.5 ±2.3	12.1 ±2.4
Total PUFA (en%)	6.0 ±1.8	5.7 ±1.5	5.5 ±1.4	5.4 ±1.5
Total SFA (en%)	11.2 ±3.1	11.4 ±2.8	11.8 ±2.8	12.5 ±2.8
SFA from total dairy (en%)	3.6 ±2.2	3.5 ±2.0	3.4 ±2.0	3.1 ±2.0
SFA from total meat (en%)	0.2 ±0.2	1.1 ±0.2	1.8 ±0.2	3.3 ±1
SFA from cereal and cereal products (en%)	3.0 ±1.7	2.9 ±1.6	2.7 ±1.5	2.4 ±1.5
SFA from fat spreads (en%)	1.2 ±1.5	1.2 ±1.4	1.2 ±1.4	1.2 ±1.4
SFA from vegetables and vegetables dishes (en%)	0.8 ±0.8	0.7 ±0.7	0.7 ±0.6	0.7 ±0.6
SFA from sugar, sweet spreads, preserves (en%)	0.6 ±0.9	0.6 ±0.9	0.5 ±0.8	0.5 ±0.8
SFA from eggs and egg products (en%)	0.4 ±0.7	0.4 ±0.6	0.3 ±0.6	0.4 ±0.6
SFA from soups, savoury sauces, samosa, pakora, sushi (en%)	0.4 ±0.4	0.4 ±0.3	0.4 ±0.3	0.4 ±0.3
SFA from fish and fish products (en%)	0.4 ±0.5	0.3 ±0.4	0.2 ±0.4	0.2 ±0.3
SFA from savoury snacks (en%)	0.2 ±0.3	0.2 ±0.3	0.2 ±0.3	0.1 ±0.3
SFA from nuts and seeds (en%)	0.3 ±0.5	0.3 ±0.4	0.2 ±0.4	0.2 ±0.4
Total TFA (en%)	0.5 ±0.2	0.5 ±0.2	0.5 ±0.2	0.6 ±0.2
Fibre (g/d)	19.6 ±6.4	18.4 ±5.7	17.5 ±5.2	15.9 ±5.2

	Quartiles of SFA intake from total meat (en%)			
**Range**	**0.0-0.7**	**0.7-1.4**	**1.4-2.3**	**2.3-17.9**
Sodium (mg/d)	1,899 ±610	1,931 ±614	1,949 ±638	2,031 ±752
Dietary cholesterol (mg/d)	204 ±133	240 ±124	254 ±124	285 ±137
Fruit and vegetables (g/d)	424.4 ±251.9	396.2 ±223.1	373.1 ±212.1	341.3 ±204.5
Total dairy (g/d)	325.9 ±183.8	333.3 ±167.4	318.7 ±160.8	287.3 ±156.1
Total meat (g/d)	25.8 ±30.4	82.4 ±36.4	109 ±40.6	147.6 ±56.8

**Data are presented as means ±SD for continuous variables or as % (n) for categorical variables, with % representing column percentages. Abbreviations. BMI: body mass index; CVD: cardiovascular diseases; en%: proportion (%) of total energy intake; METs, Metabolic equivalent of tasks; MUFA: monounsaturated fatty acids; PUFA: polyunsaturated fatty acids; SFA: saturated fatty acids; TFA: trans unsaturated fatty acids*

†Females only

Table S9. Cohort characteristics across tertiles of saturated fatty acids intake from unprocessed meat (percentage of total energy intake (%)) among 120,496 study participants in the UK Biobank*

	Tertiles of SFA from unprocessed meat (en%)		
**Range**	**0.0-0.3**	**0.3-0.8**	**0.8-10.8**
N	40,166	40,165	40,165
**Sociodemographic factors**			
Female sex	60.3 (24,223)	56.1 (22,531)	54.6 (21,935)
Age (years)	55.6 ±7.9	56.1 ±7.8	56.1 ±7.8
White Europeans	96.4 (38,723)	96.9 (38,937)	96.3 (38,668)
College or university degree/vocation	60.3 (24,224)	55.4 (22,248)	55.4 (22,236)
Least deprived	20.5 (8,225)	23 (9,258)	22 (8,839)
Country of assessment centre			
England	91 (36,549)	91.2 (36,643)	92.4 (37,106)
Scotland	5.9 (2,373)	5.5 (2,220)	4.6 (1,837)
Wales	3.1 (1,244)	3.2 (1,302)	3 (1,222)
**Lifestyle factors**			
Current smoker	6.6 (2,643)	6.6 (2,638)	7.4 (2,978)
Physical activity (excess METs)			
Low <10	26 (10,437)	27.4 (11,022)	29.9 (12,018)
Moderate 10-50	53.9 (21,640)	53.5 (21,494)	51.8 (20,823)
High ≥50	18.1 (7,282)	17.1 (6,852)	16.2 (6,526)
Dietary supplement use	54.1 (21,745)	53 (21,283)	50.2 (20,182)
Alcohol intake (g/d)			
Non-drinker	6.7 (2,696)	5 (2,003)	5.2 (2,093)

	Tertiles of SFA from unprocessed meat (en%)		
**Range**	**0.0-0.3**	**0.3-0.8**	**0.8-10.8**
<1	11.7 (4,707)	10.2 (4,102)	10.2 (4,079)
1-<10	27.6 (11,072)	27 (10,858)	26.1 (10,469)
10-<20	24.8 (9,976)	25.3 (10,147)	24.5 (9,834)
>=20	23.1 (9,278)	26.9 (10,808)	29 (11,631)
**Female factors**			
Hormone replacement therapy†	19.9 (7,975)	20.1 (8,088)	19.6 (7,878)
Menopausal status†	35.1 (14,109)	33.1 (13,304)	32.1 (12,892)
**Family history/cardiometabolic risk factors**			
Family history of CVD	55.8 (22,427)	57 (22,886)	55.8 (22,396)
BMI categories (kg/m^2)			
Underweight <18.5	0.8 (330)	0.5 (193)	0.5 (186)
Healthy weight 18.5-25	45.4 (18,242)	38.5 (15,456)	35.3 (14,171)
Overweight 25-30	37.7 (15,153)	41.6 (16,722)	42.3 (16,974)
Obesity 30<	15.8 (6,345)	19.2 (7,723)	21.8 (8,746)
Abdominal obesity	24.3 (9,776)	28.3 (11,353)	31 (12,462)
Baseline hypertension	22.1 (8,877)	24.7 (9,939)	25.5 (10,247)
Baseline hypercholesterolemia	11.2 (4,499)	13.4 (5,366)	14.2 (5,718)
Baseline diabetes mellitus	1.9 (776)	2.2 (897)	2.7 (1,087)
**Dietary intake**			
Total energy intake (kcal/d)	2,041 ±500	2,104 ±506	2,027 ±483
Total protein (en%)	14.3 ±2.6	16.0 ±2.7	17.3 ±3.0
Total carbohydrate (en%)	51.5 ±7.3	49.3 ±6.9	46.9 ±7.1

	Tertiles of SFA from unprocessed meat (en%)		
**Range**	**0.0-0.3**	**0.3-0.8**	**0.8-10.8**
Total fat (en%)	31.8 ±6.1	31.4 ±5.7	32.1 ±5.6
Total MUFA (en%)	11.5 ±2.6	11.4 ±2.4	11.6 ±2.3
Total PUFA (en%)	6.0 ±1.7	5.6 ±1.5	5.4 ±1.4
Total SFA (en%)	11.5 ±3.1	11.6 ±2.9	12.1 ±2.8
SFA from total dairy (en%)	3.6 ±2.2	3.4 ±2.0	3.2 ±1.9
SFA from milk (en%)	0.9 ±0.8	0.9 ±0.7	0.9 ±0.7
SFA from yogurt (en%)	0.3 ±0.4	0.2 ±0.4	0.2 ±0.3
SFA from cheese (en%)	1.6 ±1.5	1.3 ±1.3	1.2 ±1.3
SFA from total meat (en%)	0.7 ±0.9	1.5 ±0.8	2.7 ±1.2
SFA from unprocessed meat (en%)	0.1 ±0.1	0.5 ±0.1	1.3 ±0.6
SFA from processed meat (en%)	0.5 ±0.7	0.5 ±0.7	0.5 ±0.7
SFA from red meat (en%)	0.2 ±0.4	0.6 ±0.5	1.4 ±0.8
SFA from unprocessed red meat (en%)	0.0 ±0.1	0.6 ±0.5	1.8 ±1
SFA from processed red meat (en%)	0.4 ±0.7	0.4 ±0.7	0.4 ±0.6
SFA from poultry (en%)	0.2 ±0.2	0.4 ±0.4	0.3 ±0.4
SFA from unprocessed poultry (en%)	0.1 ±0.2	0.3 ±0.4	0.3 ±0.4
SFA from processed poultry (en%)	0 ±0.1	0.0 ±0.1	0.0 ±0.1
SFA from cereal and cereal products (en%)	2.9 ±1.7	2.8 ±1.6	2.5 ±1.5
SFA from fat spreads (en%)	1.3 ±1.5	1.2 ±1.4	1.2 ±1.3
SFA from vegetables and vegetables dishes (en%)	0.8 ±0.8	0.7 ±0.6	0.7 ±0.6

Range	Tertiles of SFA from unprocessed meat (en%)		
	0.0-0.3	0.3-0.8	0.8-10.8
SFA from sugar, sweet spreads, preserves (en%)	0.6 ±0.9	0.6 ±0.9	0.5 ±0.8
SFA from eggs and egg products (en%)	0.4 ±0.7	0.4 ±0.6	0.3 ±0.6
SFA from soups, savoury sauces, samosa, pakora, sushi (en%)	0.4 ±0.4	0.4 ±0.4	0.4 ±0.3
SFA from fish and fish products (en%)	0.3 ±0.5	0.2 ±0.4	0.2 ±0.3
SFA from savoury snacks (en%)	0.2 ±0.3	0.2 ±0.3	0.1 ±0.2
SFA from nuts and seeds (en%)	0.3 ±0.5	0.2 ±0.4	0.2 ±0.4
Total TFA (en%)	0.5 ±0.2	0.5 ±0.2	0.6 ±0.2
Fibre (g/d)	19.1 ±6.3	18 ±5.6	16.6 ±5.2
Sodium (mg/d)	2,030 ±683	1,977 ±660	1,851 ±617
Dietary cholesterol (mg/d)	218 ±139	254 ±130	266 ±125
Fruit and vegetables (g/d)	403.4 ±246.2	386.1 ±220.9	361.7 ±206.1
Total dairy (g/d)	322.6 ±179	326.0 ±168.3	300.3 ±155.6
Milk (g/d)	231.4 ±157.6	237.4 ±148.4	221.3 ±137.3
Yogurt (g/d)	47.6 ±56.1	44.4 ±53.2	39.3 ±49.8
Cheese (g/d)	20.4 ±19.8	17.1 ±17.6	15.4 ±16.2
Total meat (g/d)	42.1 ±44.1	100.1 ±46.6	131.5 ±54.7
Unprocessed meat (g/d)	18.4 ±25.7	75.3 ±36.3	109.6 ±46.6
Processed meat (g/d)	21.2 ±30.6	22.5 ±27.6	20.3 ±25.2
Red meat (g/d)	19.6 ±28.3	54.2 ±39.1	92.0 ±47.2
Unprocessed red meat (g/d)	2.3 ±9.6	34.9 ±28.5	74.2 ±40.2
Processed red meat (g/d)	17.3 ±26.3	19.3 ±24.7	17.8 ±22.7

	Tertiles of SFA from unprocessed meat (en%)		
**Range**	**0.0-0.3**	**0.3-0.8**	**0.8-10.8**
Poultry (g/d)	19.9 ±29.6	43.6 ±45.4	38.0 ±43.2
Unprocessed poultry (g/d)	16.1 ±25.2	40.4 ±44.2	35.4 ±42
Processed poultry (g/d)	3.8 ±15.1	3.2 ±12.5	2.5 ±10.7

**Data are presented as means ±SD for continuous variables or as % (n) for categorical variables, with % representing column percentages. Abbreviations. BMI: body mass index; CVD: cardiovascular diseases; en%: proportion (%) of total energy intake; METs, Metabolic equivalent of tasks; MUFA: monounsaturated fatty acids; PUFA: polyunsaturated fatty acids; SFA: saturated fatty acids; TFA: trans unsaturated fatty acids*

†Females only

Table S10. Cohort characteristics across tertiles of saturated fatty acids intake from processed meat (percentage of total energy intake (%)) among 120,496 study participants in the UK Biobank*

	Tertiles of SFA from processed meat (en%)		
**Range**	**0.0-0.0**	**0.0-0.5**	**0.5-10.5**
N	46,750	33,581	40,165
**Sociodemographic factors**			
Female sex	62.3 (29,119)	57.5 (19,293)	50.5 (20,277)
Age (years)	55.7 ±7.8	56.2 ±7.8	55.8 ±7.8
White Europeans	95.2 (44,519)	97.3 (32,658)	97.5 (39,151)
College or university degree/vocation	58.9 (27,545)	56.7 (19,028)	55.1 (22,135)
Least deprived	20.9 (9,781)	22.9 (7,687)	22 (8,854)
Country of assessment centre			
England	91.4 (42,707)	91.4 (30,689)	91.9 (36,902)
Scotland	5.4 (2,526)	5.5 (1,863)	5.1 (2,041)
Wales	3.2 (1,517)	3.1 (1,029)	3 (1,222)
**Lifestyle factors**			
Current smoker	6.4 (3,008)	5.9 (1,989)	8.1 (3,262)
Physical activity (excess METs)			
Low <10	26.4 (12,351)	27.7 (9,287)	29.5 (11,839)
Moderate 10-50	53.6 (25,068)	53.7 (18,020)	52 (20,869)
High ≥50	18 (8,415)	16.7 (5,616)	16.5 (6,629)
Alcohol intake (g/d)			
Non-drinker	6.8 (3,197)	5.2 (1,751)	4.6 (1,844)
<1	11.9 (5,586)	10.2 (3,426)	9.7 (3,876)

	Tertiles of SFA from processed meat (en%)		
Range	0.0-0.0	0.0-0.5	0.5-10.5
1-<10	28.1 (13,116)	28 (9,401)	24.6 (9,882)
10-<20	24.5 (11,435)	25.5 (8,576)	24.8 (9,946)
>=20	22.9 (10,714)	25.6 (8,610)	30.9 (12,393)
Dietary supplement use	54.8 (25,626)	52.7 (17,697)	49.5 (19,887)
**Female factors**			
Hormone replacement therapy†	21 (9,819)	20.8 (6,992)	17.8 (7,130)
Menopausal status†	36.9 (17,257)	33.9 (11,398)	29 (11,650)
**Family history/cardiometabolic risk Factors**			
Family history of CVD	56.1 (26,211)	56.7 (19,043)	55.9 (22,455)
BMI categories (kg/m^2)			
Underweight <18.5	0.8 (392)	0.5 (182)	0.3 (135)
Healthy weight 18.5-25	44.5 (20,791)	39.9 (13,404)	34 (13,674)
Overweight 25-30	38.5 (17,993)	41 (13,756)	42.6 (17,100)
Obesity 30<	16 (7,460)	18.4 (6,177)	22.8 (9,177)
Abdominal obesity	24.6 (11,503)	27.5 (9,229)	32 (12,859)
Baseline hypertension	22.3 (10,413)	24.5 (8,239)	25.9 (10,411)
Baseline hypercholesterolemia	11.6 (5,435)	13.2 (4,422)	14.3 (5,726)
Baseline diabetes mellitus	1.9 (881)	2.2 (728)	2.9 (1,151)
**Dietary intake**			
Total energy intake (kcal/d)	1,976 ±487	2,108 ±487	2,110 ±506
Total protein (en%)	15.7 ±3.2	15.8 ±2.8	16.1 ±2.9
Total carbohydrate (en%)	50.5 ±7.4	49.7 ±6.8	47.3 ±7.3

	Tertiles of SFA from processed meat (en%)		
**Range**	**0.0-0.0**	**0.0-0.5**	**0.5-10.5**
Total fat (en%)	31.1 ±6	31.4 ±5.5	32.9 ±5.6
Total MUFA (en%)	11.2 ±2.5	11.3 ±2.3	12 ±2.4
Total PUFA (en%)	5.7 ±1.7	5.6 ±1.5	5.7 ±1.4
Total SFA (en%)	11.4 ±3	11.6 ±2.8	12.2 ±2.8
SFA from total dairy (en%)	3.4 ±2.2	3.4 ±1.9	3.3 ±2
SFA from milk (en%)	0.9 ±0.8	0.9 ±0.7	0.9 ±0.7
SFA from yogurt (en%)	0.3 ±0.4	0.2 ±0.3	0.2 ±0.3
SFA from cheese (en%)	1.5 ±1.5	1.4 ±1.3	1.3 ±1.3
SFA from total meat (en%)	1.1 ±1.2	1.4 ±1	2.4 ±1.2
SFA from unprocessed meat (en%)	0.6 ±0.6	0.7 ±0.6	0.7 ±0.6
SFA from processed meat (en%)	0 ±0	0.2 ±0.1	1.2 ±0.7
SFA from red meat (en%)	0.4 ±0.6	0.7 ±0.6	1.2 ±0.8
SFA from unprocessed red meat (en%)	0.8 ±1.1	0.9 ±0.9	0.9 ±1
SFA from processed red meat (en%)	0 ±0	0.2 ±0.1	1.2 ±0.8
SFA from poultry (en%)	0.3 ±0.4	0.3 ±0.3	0.3 ±0.4
SFA from unprocessed poultry (en%)	0.3 ±0.4	0.3 ±0.3	0.2 ±0.3
SFA from processed poultry (en%)	0 ±0	0 ±0.1	0.1 ±0.2
SFA from cereal and cereal products (en%)	2.7 ±1.7	2.9 ±1.6	2.6 ±1.5
SFA from fat spreads (en%)	1.2 ±1.4	1.2 ±1.4	1.3 ±1.4
SFA from vegetables and vegetables dishes (en%)	0.8 ±0.8	0.7 ±0.6	0.7 ±0.6

Range	Tertiles of SFA from processed meat (en%)		
	0.0-0.0	0.0-0.5	0.5-10.5
SFA from sugar, sweet spreads, preserves (en%)	0.6 ±0.9	0.6 ±0.8	0.5 ±0.8
SFA from eggs and egg products (en%)	0.4 ±0.7	0.3 ±0.6	0.4 ±0.6
SFA from soups, savoury sauces, samosa, pakora, sushi (en%)	0.4 ±0.4	0.4 ±0.3	0.3 ±0.3
SFA from fish and fish products (en%)	0.3 ±0.5	0.2 ±0.4	0.2 ±0.4
SFA from savoury snacks (en%)	0.1 ±0.3	0.2 ±0.3	0.2 ±0.3
SFA from nuts and seeds (en%)	0.3 ±0.5	0.2 ±0.4	0.2 ±0.4
Total TFA (en%)	0.5 ±0.2	0.5 ±0.2	0.5 ±0.2
Fibre (g/d)	18.3 ±6.3	18.2 ±5.5	17.1 ±5.4
Sodium (mg/d)	1,728 ±573	1,928 ±569	2,235 ±712
Dietary cholesterol (mg/d)	223 ±131	243 ±121	275 ±139
Fruit and vegetables (g/d)	405.6 ±241.8	391.8 ±215.9	351.7 ±210
Total dairy (g/d)	314.5 ±176.4	329.4 ±163	307.4 ±162.1
Milk (g/d)	226.1 ±155.7	238.9 ±143.3	227.2 ±142.8
Yogurt (g/d)	47 ±55.7	45.8 ±53	38.3 ±50
Cheese (g/d)	18.3 ±18.9	17.8 ±17.4	16.8 ±17.5
Total meat (g/d)	65.6 ±58.1	92.9 ±51.3	119.6 ±59.4
Unprocessed meat (g/d)	63.7 ±56.6	74 ±49.2	67.3 ±50.9
Processed meat (g/d)	0 ±0	16.7 ±11.5	50 ±29.2
Red meat (g/d)	33.6 ±42.5	53.7 ±41.1	81.8 ±49.1

	Tertiles of SFA from processed meat (en%)		
**Range**	**0.0-0.0**	**0.0-0.5**	**0.5-10.5**
Unprocessed red meat (g/d)	33.6 ±42.5	40.6 ±40	38.3 ±40.6
Processed red meat (g/d)	0 ±0	13.1 ±9.4	43.5 ±26.4
Poultry (g/d)	30 ±41.5	37.1 ±39.7	35.5 ±41.9
Unprocessed poultry (g/d)	30 ±41.5	33.4 ±38.7	29 ±37.7
Processed poultry (g/d)	0 ±0	3.6 ±10.6	6.5 ±19.6

**Data are presented as means ±SD for continuous variables or as % (n) for categorical variables, with % representing column percentages. Abbreviations. BMI: body mass index; CVD: cardiovascular diseases; en%: proportion (%) of total energy intake; METs, Metabolic equivalent of tasks; MUFA: monounsaturated fatty acids; PUFA: polyunsaturated fatty acids; SFA: saturated fatty acids; TFA: trans unsaturated fatty acids*

†Females only

Table S11. Cohort characteristics across tertiles of saturated fatty acids intake from red meat (percentage of total energy intake (%)) among 120,496 study participants in the UK Biobank*

	Tertiles of SFA from red meat (en%)		
**Range**	**0-0.3**	**0.3-0.9**	**0.9-10.8**
N	40,166	40,165	40,165
**Sociodemographic factors**			
Female sex	63.3 (25,438)	56.5 (22,688)	51.2 (20,563)
Age (years)	55.5 ±7.9	56.1 ±7.8	56.2 ±7.7
White Europeans	95.6 (38,391)	97 (38,968)	97 (38,969)
College or university degree/vocation	59.7 (23,979)	56 (22,497)	55.4 (22,232)
Least deprived	20.5 (8,243)	23.2 (9,321)	21.8 (8,758)
Country of assessment centre			
England	91.1 (36,588)	91.2 (36,640)	92.3 (37,070)
Scotland	5.7 (2,290)	5.7 (2,270)	4.7 (1,870)
Wales	3.2 (1,288)	3.1 (1,255)	3 (1,225)
**Lifestyle factors**			
Current smoker	6 (2,394)	6.5 (2,612)	8.1 (3,253)
Physical activity (excess METs)			
Low <10	25.8 (10,371)	27.3 (10,981)	30.2 (12,125)
Moderate 10-50	54.1 (21,727)	53.4 (21,458)	51.7 (20,772)
High ≥50	18.1 (7,276)	17.2 (6,918)	16.1 (6,466)
Dietary supplement use	55.3 (22,210)	52.6 (21,131)	49.5 (19,869)
Alcohol intake (g/d)			
Non-drinker	6.9 (2,788)	5.1 (2,040)	4.9 (1,964)

	Tertiles of SFA from red meat (en%)		
**Range**	**0-0.3**	**0.3-0.9**	**0.9-10.8**
<1	12.2 (4,918)	10.3 (4,127)	9.6 (3,843)
1-<10	28.6 (11,487)	26.9 (10,824)	25.1 (10,088)
10-<20	24.8 (9,944)	25.3 (10,160)	24.5 (9,853)
>=20	21.4 (8,590)	26.9 (10,788)	30.7 (12,339)
**Female factors**			
Hormone replacement therapy	20.9 (8,403)	20.3 (8,140)	18.4 (7,398)
Menopausal status	36.8 (14,765)	33.4 (13,399)	30.2 (12,141)
**Family history/cardiometabolic risk factors**			
Family history of CVD	56.2 (22,585)	56.4 (22,645)	56 (22,479)
BMI categories (kg/m^2)			
Underweight <18.5	0.9 (351)	0.5 (204)	0.4 (154)
Healthy weight 18.5-25	46.1 (18,531)	39.1 (15,719)	33.9 (13,619)
Overweight 25-30	37.5 (15,067)	41.4 (16,636)	42.7 (17,146)
Obesity 30<	15.2 (6,118)	18.8 (7,540)	22.8 (9,156)
Abdominal obesity	23.6 (9486)	27.9 (11,194)	32.1 (12,911)
Baseline hypertension	21.7 (8,706)	24.5 (9,821)	26.2 (10,536)
Baseline hypercholesterolemia	10.9 (4,384)	13.1 (5,258)	14.8 (5,941)
Baseline diabetes mellitus	1.7 (677)	2.3 (918)	2.9 (1,165)
**Dietary intake**			
Total energy intake (kcal/d)	1,997 ±501	2,110 ±492	2,065 ±493
Total protein (en%)	15.0 ±3.1	15.8 ±2.8	16.8 ±2.9
Total carbohydrate (en%)	51.7 ±7.3	49.4 ±6.8	46.6 ±7.1

	Tertiles of SFA from red meat (en%)		
**Range**	**0-0.3**	**0.3-0.9**	**0.9-10.8**
Total fat (en%)	31.0 ±6.1	31.5 ±5.6	32.8 ±5.5
Total MUFA (en%)	11.2 ±2.6	11.4 ±2.3	11.9 ±2.3
Total PUFA (en%)	6.0 ±1.7	5.6 ±1.5	5.4 ±1.4
Total SFA (en%)	11.0 ±3	11.7 ±2.8	12.4 ±2.8
SFA from total dairy (en%)	3.5 ±2.2	3.4 ±2	3.2 ±1.9
SFA from milk (en%)	0.9 ±0.8	0.9 ±0.7	0.9 ±0.7
SFA from yogurt (en%)	0.3 ±0.4	0.2 ±0.4	0.2 ±0.3
SFA from cheese (en%)	1.5 ±1.5	1.3 ±1.3	1.3 ±1.3
SFA from total meat (en%)	0.5 ±0.6	1.5 ±0.6	2.9 ±1.1
SFA from unprocessed meat (en%)	0.2 ±0.2	0.5 ±0.3	1.2 ±0.7
SFA from processed meat (en%)	0.1 ±0.2	0.4 ±0.5	0.9 ±0.9
SFA from red meat (en%)	0.1 ±0.1	0.6 ±0.2	1.6 ±0.7
SFA from unprocessed red meat (en%)	0.0 ±0.1	0.7 ±0.6	1.7 ±1.1
SFA from processed red meat (en%)	0.1 ±0.1	0.4 ±0.5	0.8 ±0.9
SFA from poultry (en%)	0.3 ±0.4	0.3 ±0.3	0.2 ±0.3
SFA from unprocessed poultry (en%)	0.3 ±0.4	0.3 ±0.3	0.2 ±0.3
SFA from processed poultry (en%)	0 ±0.1	0 ±0.1	0 ±0.1
SFA from cereal and cereal products (en%)	2.9 ±1.7	2.8 ±1.6	2.5 ±1.5
SFA from fat spreads (en%)	1.2 ±1.4	1.2 ±1.4	1.3 ±1.4
SFA from vegetables and vegetables dishes (en%)	0.8 ±0.8	0.7 ±0.7	0.7 ±0.6

Range	Tertiles of SFA from red meat (en%)		
	0-0.3	0.3-0.9	0.9-10.8
SFA from sugar, sweet spreads, preserves (en%)	0.6 ±0.9	0.6 ±0.9	0.5 ±0.8
SFA from eggs and egg products (en%)	0.4 ±0.7	0.4 ±0.6	0.4 ±0.6
SFA from soups, savoury sauces, samosa, pakora, sushi (en%)	0.6 ±0.9	0.6 ±0.9	0.5 ±0.8
SFA from fish and fish products (en%)	0.3 ±0.5	0.2 ±0.4	0.2 ±0.3
SFA from savoury snacks (en%)	0.2 ±0.3	0.2 ±0.3	0.1 ±0.2
SFA from nuts and seeds (en%)	0.3 ±0.5	0.2 ±0.4	0.2 ±0.4
Total TFA (en%)	0.4 ±0.2	0.5 ±0.2	0.6 ±0.2
Fibre (g/d)	18.9 ±6.4	18.1 ±5.6	16.6 ±5.2
Sodium (mg/d)	1,845 ±614	1,969 ±632	2,044 ±709
Dietary cholesterol (mg/d)	217 ±136	248 ±128	272.7 ±130
Fruit and vegetables (g/d)	416.7 ±248.2	384.8 ±220	349.8 ±201.4
Total dairy (g/d)	320.5 ±179.7	327.9 ±167.4	300.5 ±155.7
Milk (g/d)	228.8 ±158.2	239.0 ±147.6	222.2 ±137.5
Yogurt (g/d)	49.4 ±56.9	44.3 ±52.9	37.6 ±48.8
Cheese (g/d)	19.3 ±19.5	17.5 ±17.6	16.1 ±16.8
Total meat (g/d)	49.5 ±52.2	94.4 ±47.6	129.7 ±54.9
Unprocessed meat (g/d)	38.9 ±47	70.9 ±46	93.5 ±50.7
Processed meat (g/d)	8.5 ±17.4	21.2 ±24.4	34.3 ±33.2
Red meat (g/d)	8.4 ±14.7	56.1 ±26.8	101.3 ±44

	Tertiles of SFA from red meat (en%)		
**Range**	**0-0.3**	**0.3-0.9**	**0.9-10.8**
Unprocessed red meat (g/d)	3.4 ±11.7	38.2 ±30	69.7 ±43.3
Processed red meat (g/d)	5 ±9.5	17.9 ±20.5	31.5 ±30.9
Poultry (g/d)	39.0 ±47.5	35.9 ±40	26.5 ±34.2
Unprocessed poultry (g/d)	35.5 ±45.5	32.6 ±38.6	23.8 ±32.5
Processed poultry (g/d)	3.5 ±14.3	3.3 ±12.9	2.8 ±11.3

**Data are presented as means ±SD for continuous variables or as % (n) for categorical variables, with % representing column percentages. Abbreviations. BMI: body mass index; CVD: cardiovascular diseases; en%: proportion (%) of total energy intake; METs, Metabolic equivalent of tasks; MUFA: monounsaturated fatty acids; PUFA: polyunsaturated fatty acids; SFA: saturated fatty acids; TFA: trans unsaturated fatty acids*

†Females only

Table S12. Cohort characteristics across tertiles of saturated fatty acids intake from unprocessed red meat (percentage of total energy intake (%)) among 120,496 study participants in the UK Biobank*

	Tertiles of SFA from unprocessed red meat (en%)		
**Range**	**(0.0-0.0)**	**(0.1-1.1)**	**(1.1-17.9)**
N	49,462	30,869	40,165
**Sociodemographic factors**			
Female sex	61.5 (30,423)	54.5 (16,816)	53.4 (21,450)
Age (years)	55.4 ±7.9	56.3 ±7.7	56.2 ±7.8
White Europeans	96.1 (47,551)	97.2 (30,018)	96.5 (38,759)
College or university degree/vocation	59 (29,187)	56.7 (17,516)	54.8 (22,005)
Least deprived	20.7 (10,214)	23 (7,095)	22.4 (9,013)
Country of assessment centre			
England	91.1 (45,052)	91.8 (28,343)	91.9 (36,903)
Scotland	5.7 (2,815)	5.3 (1,626)	5 (1,989)
Wales	3.2 (1,595)	2.9 (900)	3.2 (1,273)
**Lifestyle factors**			
Current smoker	6.5 (3,213)	6.3 (1,945)	7.7 (3,101)
Physical activity (excess METs)			
Low <10	26.6 (13,136)	27.2 (8,406)	29.7 (11,935)
Moderate 10-50	53.8 (26,626)	53.3 (16,457)	52 (20,874)
High ≥50	17.6 (8,719)	17.6 (5,426)	16.2 (6,515)
Dietary supplement use	54.2 (26,810)	52.7 (16,274)	50.1 (20,126)
Alcohol intake (g/d)			
Non-drinker	6.6 (3,251)	5 (1,552)	5 (1,989)
<1	11.8 (5,831)	10.2 (3,138)	9.8 (3,919)

	Tertiles of SFA from unprocessed red meat (en%)		
**Range**	**(0.0-0.0)**	**(0.1-1.1)**	**(1.1-17.9)**
1-<10	28 (13,827)	27.1 (8,354)	25.4 (10,218)
10-<20	24.9 (12,295)	25.2 (7,778)	24.6 (9,884)
>=20	22.7 (11,212)	27.5 (8,481)	29.9 (12,024)
**Female factors**			
Hormone replacement therapy†	20.4 (10,110)	19.5 (6,027)	19.4 (7,804)
Menopausal status†	35.5 (17,574)	32.5 (10,018)	31.7 (12,713)
**Family history/cardiometabolic risk Factors**			
Family history of CVD	56.2 (27,798)	56.7 (17,514)	55.8 (22,397)
BMI categories (kg/m^2)			
Underweight <18.5	0.8 (386)	0.5 (156)	0.4 (167)
Healthy weight 18.5-25	44.1 (21,790)	39.2 (12,103)	34.8 (13,976)
Overweight 25-30	38.3 (18,922)	41.4 (12,775)	42.7 (17,152)
Obesity 30<	16.7 (8,247)	18.8 (5,788)	21.9 (8,779)
Abdominal obesity	25.2 (12,470)	28 (8,648)	31.1 (12,473)
Baseline hypertension	22.2 (10,990)	25.2 (7,791)	25.6 (10,282)
Baseline hypercholesterolemia	11.4 (5,617)	13.6 (4,185)	14.4 (5,781)
Baseline diabetes mellitus	1.9 (937)	2.3 (697)	2.8 (1,126)
**Dietary intake**			
Total energy intake (kcal/d)	1,995 ±499	2,170 ±489	2,047 ±489
Total protein (en%)	15.1 ±3.1	15.8 ±2.6	16.9 ±3
Total carbohydrate (en%)	51 ±7.4	49.3 ±6.7	47.1 ±7.2
Total fat (en%)	31.5 ±6.1	31.6 ±5.4	32.2 ±5.6

	Tertiles of SFA from unprocessed red meat (en%)		
**Range**	**(0.0-0.0)**	**(0.1-1.1)**	**(1.1-17.9)**
Total MUFA (en%)	11.4 ±2.6	11.5 ±2.3	11.6 ±2.4
Total PUFA (en%)	6 ±1.7	5.6 ±1.4	5.3 ±1.4
Total SFA (en%)	11.3 ±3	11.7 ±2.8	12.2 ±2.8
SFA from total dairy (en%)	3.5 ±2.1	3.4 ±1.9	3.2 ±2
SFA from milk (en%)	0.9 ±0.8	0.9 ±0.7	0.9 ±0.7
SFA from yogurt (en%)	0.3 ±0.4	0.2 ±0.3	0.2 ±0.3
SFA from cheese (en%)	1.5 ±1.5	1.3 ±1.3	1.3 ±1.3
SFA from total meat (en%)	0.8 ±0.9	1.5 ±0.8	2.7 ±1.2
SFA from unprocessed meat (en%)	0.2 ±0.2	0.6 ±0.3	1.3 ±0.6
SFA from processed meat (en%)	0.4 ±0.7	0.5 ±0.6	0.5 ±0.7
SFA from red meat (en%)	0.2 ±0.4	0.7 ±0.5	1.4 ±0.8
SFA from unprocessed red meat (en%)	0 ±0	0.7 ±0.2	2 ±0.9
SFA from processed red meat (en%)	0.4 ±0.7	0.5 ±0.6	0.4 ±0.7
SFA from poultry (en%)	0.3 ±0.4	0.3 ±0.3	0.2 ±0.3
SFA from unprocessed poultry (en%)	0.3 ±0.4	0.2 ±0.3	0.2 ±0.3
SFA from processed poultry (en%)	0 ±0.1	0 ±0.1	0 ±0.1
SFA from cereal and cereal products (en%)	2.8 ±1.7	2.9 ±1.5	2.6 ±1.5
SFA from fat spreads (en%)	1.2 ±1.5	1.2 ±1.4	1.2 ±1.4
SFA from vegetables and vegetables dishes (en%)	0.8 ±0.8	0.7 ±0.6	0.7 ±0.6

Range	Tertiles of SFA from unprocessed red meat (en%)		
	(0.0-0.0)	(0.1-1.1)	(1.1-17.9)
SFA from sugar, sweet spreads, preserves (en%)	0.6 ±0.9	0.6 ±0.8	0.5 ±0.8
SFA from eggs and egg products (en%)	0.4 ±0.7	0.4 ±0.6	0.3 ±0.6
SFA from soups, savoury sauces, samosa, pakora, sushi (en%)	0.4 ±0.4	0.4 ±0.3	0.4 ±0.4
SFA from fish and fish products (en%)	0.3 ±0.5	0.2 ±0.4	0.2 ±0.4
SFA from savoury snacks (en%)	0.2 ±0.3	0.2 ±0.3	0.1 ±0.3
SFA from nuts and seeds (en%)	0.3 ±0.5	0.2 ±0.4	0.2 ±0.4
Total TFA (en%)	0.5 ±0.2	0.5 ±0.2	0.6 ±0.2
Fibre (g/d)	18.5 ±6.3	18.4 ±5.5	16.7 ±5.3
Sodium (mg/d)	1,957 ±678	2,041 ±647	1,880 ±632
Dietary cholesterol (mg/d)	227 ±139	256 ±125	262 ±129
Fruit and vegetables (g/d)	400.6 ±242.6	388.6 ±216.7	359.3 ±207.9
Total dairy (g/d)	315.7 ±175.4	332.6 ±166.2	304.4 ±159.6
Milk (g/d)	226.8 ±154.1	241.8 ±147.4	224.9 ±140.5
Yogurt (g/d)	47.4 ±55.8	44.4 ±52.2	38.8 ±50.3
Cheese (g/d)	19 ±19.2	17.9 ±17.2	15.8 ±17
Total meat (g/d)	58.1 ±55.7	98.8 ±48	126.2 ±55.1
Unprocessed meat (g/d)	35.6 ±44.9	72.5 ±39.2	103.7 ±46.3
Processed meat (g/d)	20.3 ±29.3	23.7 ±26.9	20.8 ±26.7
Red meat (g/d)	16.7 ±25.4	60.1 ±29.7	99.0 ±44.1

	Tertiles of SFA from unprocessed red meat (en%)		
**Range**	**(0.0-0.0)**	**(0.1-1.1)**	**(1.1-17.9)**
Unprocessed red meat (g/d)	0 ±0	39.7 ±16.1	80.9 ±36.1
Processed red meat (g/d)	16.7 ±25.4	20.4 ±24	18.2 ±24
Poultry (g/d)	39.1 ±46.9	36.2 ±37.8	25.5 ±34.6
Unprocessed poultry (g/d)	35.6 ±44.9	32.9 ±36.4	22.9 ±33
Processed poultry (g/d)	3.5 ±14.2	3.4 ±12.3	2.6 ±11.6

**Data are presented as means ±SD for continuous variables or as % (n) for categorical variables, with % representing column percentages. Abbreviations. BMI: body mass index; CVD: cardiovascular diseases; en%: proportion (%) of total energy intake; METs, Metabolic equivalent of tasks; MUFA: monounsaturated fatty acids; PUFA: polyunsaturated fatty acids; SFA: saturated fatty acids; TFA: trans unsaturated fatty acids*

†Females only

Table S13. Cohort characteristics across tertiles of saturated fatty acids intake from processed red meat (percentage of total energy intake (%)) among 120,496 study participants in the UK Biobank*

	Tertiles of SFA from processed red meat (en%)		
**Range**	**0.0-0.0**	**0.0-0.4**	**0.4-10.5**
N	50,329	30,002	40,165
**Sociodemographic factors**			
Female sex	61.6 (31,013)	58.1 (17,445)	50.4 (20,231)
Age, years	55.7 ±7.9	56.3 ±7.8	55.9 ±7.8
White Europeans	94.9 (47,768)	97.6 (29,280)	97.8 (39,280)
College or university degree/vocation	58.3 (29,361)	56.5 (16,954)	55.8 (22,393)
Least deprived	20.8 (10,468)	23 (6,889)	22.3 (8,965)
Country of assessment centre			
England	91.3 (45,953)	91.4 (27,429)	91.9 (36,916)
Scotland	5.5 (2,754)	5.5 (1,644)	5.1 (2,032)
Wales	3.2 (1,622)	3.1 (929)	3 (1,217)
**Lifestyle factors**			
Current smoker	6.5 (3,277)	5.9 (1,766)	8 (3,216)
Physical activity (excess METs)			
Low <10	26.7 (13,414)	27.7 (8,313)	29.3 (11,750)
Moderate 10-50	53.4 (26,889)	53.6 (16,096)	52.2 (20,972)
High ≥50	17.9 (9,031)	16.7 (5,007)	16.5 (6,622)
Dietary supplement use	54.6 (27,497)	52.7 (15,799)	49.6 (19,914)
Alcohol intake (g/d)			
Non-drinker	6.9 (3,494)	5 (1,507)	4.5 (1,791)

	**Tertiles of SFA from processed red meat (en%)**		
**Range**	**0.0-0.0**	**0.0-0.4**	**0.4-10.5**
<1	12 (6,061)	10.3 (3,080)	9.3 (3,747)
1-<10	28 (14,070)	28.1 (8,425)	24.7 (9,904)
10-<20	24.3 (12,210)	25.6 (7,678)	25.1 (10,069)
>=20	23 (11,586)	25.6 (7,670)	31 (12,461)
**Female factors**			
Hormone replacement therapy	20.8 (10,475)	21.2 (6,354)	17.7 (7,112)
Menopausal status	36.3 (18,263)	34.4 (10,317)	29.2 (11,725)
**Family history/cardiometabolic risk Factors**			
Family history of CVD	56 (28,181)	56.9 (17,079)	55.9 (22,449)
BMI categories (kg/m^2)			
Underweight <18.5	0.8 (403)	0.6 (177)	0.3 (129)
Healthy weight 18.5-25	43.8 (22,057)	39.9 (11,959)	34.5 (13,853)
Overweight 25-30	38.8 (19,507)	41 (12,315)	42.4 (17,027)
Obesity 30<	16.4 (8,236)	18.3 (5,496)	22.6 (9,082)
Abdominal obesity	24.9 (12,554)	27.6 (8,272)	31.8 (12,765)
Baseline hypertension	22.5 (11,313)	24.5 (7,345)	25.9 (10,405)
Baseline hypercholesterolemia	11.7 (5,892)	13.3 (3,984)	14.2 (5,707)
Baseline diabetes mellitus	1.9 (957)	2.2 (662)	2.8 (1,141)
**Dietary intake**			
Total energy intake (kcal/d)	1,979 ±489	2,102 ±488	2,122 ±502
Total protein (en%)	15.7 ±3.2	15.8 ±2.8	16.1 ±2.9
Total carbohydrate (en%)	50.5 ±7.4	49.7 ±6.8	47.3 ±7.3

	Tertiles of SFA from processed red meat (en%)		
**Range**	**0.0-0.0**	**0.0-0.4**	**0.4-10.5**
Total fat (en%)	31.1 ±6	31.3 ±5.5	32.9 ±5.6
Total MUFA (en%)	11.2 ±2.5	11.3 ±2.3	12.0 ±2.3
Total PUFA (en%)	5.7 ±1.7	5.6 ±1.5	5.6 ±1.4
Total SFA (en%)	11.3 ±3	11.6 ±2.8	12.3 ±2.8
SFA from total dairy (en%)	3.4 ±2.1	3.4 ±1.9	3.3 ±2
SFA from milk (en%)	0.9 ±0.8	0.9 ±0.7	0.9 ±0.7
SFA from yogurt (en%)	0.3 ±0.4	0.2 ±0.3	0.2 ±0.3
SFA from cheese (en%)	1.4 ±1.5	1.4 ±1.3	1.3 ±1.3
SFA from total meat (en%)	1.1 ±1.1	1.4 ±1	2.4 ±1.2
SFA from unprocessed meat (en%)	0.6 ±0.6	0.7 ±0.6	0.7 ±0.6
SFA from processed meat (en%)	0 ±0.1	0.2 ±0.2	1.2 ±0.7
SFA from red meat (en%)	0.4 ±0.6	0.7 ±0.6	1.2 ±0.8
SFA from unprocessed red meat (en%)	0.8 ±1.1	0.9 ±0.9	0.9 ±1
SFA from processed red meat (en%)	0 ±0	0.2 ±0.1	1.2 ±0.7
SFA from poultry (en%)	0.3 ±0.4	0.3 ±0.3	0.3 ±0.3
SFA from unprocessed poultry (en%)	0.3 ±0.4	0.3 ±0.3	0.2 ±0.3
SFA from processed poultry (en%)	0.0 ±0.1	0.0 ±0.1	0.0 ±0.1
SFA from cereal and cereal products (en%)	2.7 ±1.7	2.9 ±1.6	2.7 ±1.5
SFA from fat spreads (en%)	1.1 ±1.4	1.2 ±1.4	1.3 ±1.4
SFA from vegetables and vegetables dishes (en%)	0.8 ±0.8	0.7 ±0.6	0.7 ±0.6

Range	Tertiles of SFA from processed red meat (en%)		
	0.0-0.0	0.0-0.4	0.4-10.5
SFA from eggs and egg products (en%)	0.4 ±0.7	0.3 ±0.6	0.4 ±0.6
SFA from soups, savoury sauces, samosa, pakora, sushi (en%)	0.4 ±0.4	0.4 ±0.3	0.4 ±0.3
SFA from fish and fish products (en%)	0.3 ±0.5	0.2 ±0.4	0.2 ±0.4
SFA from savoury snacks (en%)	0.2 ±0.3	0.2 ±0.3	0.2 ±0.3
SFA from nuts and seeds (en%)	0.3 ±0.5	0.2 ±0.4	0.2 ±0.4
Total TFA (en%)	0.5 ±0.2	0.5 ±0.2	0.5 ±0.2
Fibre (g/d)	18.2 ±6.3	18.2 ±5.4	17.2 ±5.4
Sodium (mg/d)	1,733 ±573	1,928 ±568	2,246 ±707
Dietary cholesterol (mg/d)	222 ±131	243 ±121	278 ±138
Fruit and vegetables (g/d)	401.5 ±240.6	393.1 ±216.1	354.6 ±209.9
Total dairy (g/d)	314.4 ±176.1	329.7 ±162.1	308.6 ±162.1
Milk (g/d)	226.5 ±155.4	238.8 ±142.2	227.8 ±142.9
Yogurt (g/d)	46.6 ±55.4	46.1 ±53.2	38.5 ±50
Cheese (g/d)	18 ±18.8	17.8 ±17.5	17.1 ±17.5
Total meat (g/d)	68.4 ±58.8	93.4 ±52	118.2 ±59.1
Unprocessed meat (g/d)	63.4 ±56.1	74.2 ±49.4	68.4 ±50.8
Processed meat (g/d)	3 ±13	17.1 ±15.4	47.5 ±28.8
Red meat (g/d)	33.5 ±42.2	54.3 ±40.8	83.2 ±48.2

	Tertiles of SFA from processed red meat (en%)		
**Range**	**0.0-0.0**	**0.0-0.4**	**0.4-10.5**
Unprocessed red meat (g/d)	33.5 ±42.2	40.7 ±40	39.0 ±40.7
Processed red meat (g/d)	0 ±0	13.7 ±8.8	44.2 ±25.5
Poultry (g/d)	32.9 ±43.1	36.9 ±40.2	32.6 ±39.6
Unprocessed poultry (g/d)	29.9 ±41.2	33.5 ±38.7	29.4 ±37.8
Processed poultry (g/d)	3 ±13	3.4 ±12.6	3.2 ±13

**Data are presented as means ±SD for continuous variables or as % (n) for categorical variables, with % representing column percentages. Abbreviations. BMI: body mass index; CVD: cardiovascular diseases; en%: proportion (%) of total energy intake; METs, Metabolic equivalent of tasks; MUFA: monounsaturated fatty acids; PUFA: polyunsaturated fatty acids; SFA: saturated fatty acids; TFA: trans unsaturated fatty acids*

†Females only

Table S14. Cohort characteristics in non-consumers and consumers of saturated fatty acids intake from poultry (percentage of total energy intake (%)) among 120,496 study participants in the UK Biobank*

	**SFA from poultry (en%)**	
**Range**	**Zero consumers**	**Consumers (0-5.5)**
N	55,038	65,458
**Sociodemographic factors**		
Female sex	57.3 (31,524)	56.8 (37,165)
Age (years)	56.3 ±7.8	55.6 ±7.9
White Europeans	96.7 (53,249)	96.4 (63,079)
College or university degree/vocation	58.5 (32,217)	55.7 (36,491)
Least deprived	21.1 (11,595)	22.5 (14,727)
Country of assessment centre		
England	91.5 (50,376)	91.5 (59,922)
Scotland	5.4 (2,971)	5.3 (3,459)
Wales	3.1 (1,691)	3.2 (2,077)
**Lifestyle factors**		
Current smoker	7.3 (4,011)	6.5 (4,248)
Physical activity (excess METs)		
Low <10	27 (14,867)	28.4 (18,610)
Moderate 10-50	53.1 (29,243)	53 (34,714)
High ≥50	17.8 (9,775)	16.6 (10,885)
Dietary supplement use	52.7 (29,008)	52.3 (34,202)
Alcohol intake (g/d)		
Non-drinker	6 (3,305)	5.3 (3,487)

	SFA from poultry (en%)	
**Range**	**Zero consumers**	**Consumers (0-5.5)**
<1	10.9 (6,014)	10.5 (6,874)
1-<10	26.8 (14,737)	27 (17,662)
10-<20	24.8 (13,649)	24.9 (16,308)
>=20	25.8 (14,185)	26.8 (17,532)
**Female factors**		
Hormone replacement therapy†	19.8 (10,883)	19.9 (13,058)
Menopausal status†	34.7 (19,074)	32.4 (21,231)
**Family history/cardiometabolic risk factors**		
Family history of CVD	56.2 (30,937)	56.2 (36,772)
BMI categories (kg/m^2)		
Underweight <18.5	0.7 (376)	333 (0.5)
Healthy weight 18.5-25	41.7 (22,977)	38 (24,892)
Overweight 25-30	39.7 (21,833)	41.3 (27,016)
Obesity 30<	17.7 (9,722)	20 (13,092)
Abdominal obesity	26.6 (14,611)	29 (18,980)
Baseline hypertension	23.8 (13,112)	24.4 (15,951)
Baseline hypercholesterolemia	12.8 (7,026)	13.1 (8,557)
Baseline diabetes mellitus	2.5 (1,362)	2.1 (1,398)
**Dietary intake**		
Total energy intake (kcal/d)	2,042 ±499	2,070 ±496
Total protein (en%)	15 ±2.8	16.6 ±3
Total carbohydrate (en%)	49.9 ±7.5	48.6 ±7.2
Total fat (en%)	32.1 ±5.9	31.4 ±5.6

Range	SFA from poultry (en%)	
	Zero consumers	Consumers (0-5.5)
Total MUFA (en%)	11.5 ±2.5	11.5 ±2.4
Total PUFA (en%)	5.6 ±1.6	5.7 ±1.5
Total SFA (en%)	12 ±3	11.5 ±2.8
SFA from total dairy (en%)	3.5 ±2.1	3.2 ±1.9
SFA from milk (en%)	0.9 ±0.8	0.9 ±0.7
SFA from yogurt (en%)	0.2 ±0.4	0.2 ±0.4
SFA from cheese (en%)	1.5 ±1.5	1.2 ±1.3
SFA from total meat (en%)	1.5 ±1.4	1.7 ±1.1
SFA from unprocessed meat (en%)	0.5 ±0.6	0.7 ±0.6
SFA from processed meat (en%)	0.4 ±0.7	0.5 ±0.7
SFA from red meat (en%)	0.8 ±0.8	0.7 ±0.7
SFA from unprocessed red meat (en%)	1 ±1.1	0.7 ±0.9
SFA from processed red meat (en%)	0.4 ±0.7	0.4 ±0.6
SFA from poultry (en%)	0 ±0	0.5 ±0.3
SFA from unprocessed poultry (en%)	0 ±0	0.5 ±0.3
SFA from processed poultry (en%)	0 ±0	0.1 ±0.2
SFA from cereal and cereal products (en%)	2.8 ±1.7	2.7 ±1.6
SFA from fat spreads (en%)	1.3 ±1.5	1.2 ±1.3
SFA from vegetables and vegetables dishes (en%)	0.8 ±0.7	0.7 ±0.6
SFA from sugar, sweet spreads, preserves (en%)	0.6 ±0.9	0.6 ±0.9
SFA from eggs and egg products (en%)	0.4 ±0.7	0.4 ±0.6
SFA from soups, savoury sauces, samosa, pakora, sushi (en%)	0.4 ±0.4	0.4 ±0.3

	SFA from poultry (en%)	
Range	Zero consumers	Consumers (0-5.5)
SFA from fish and fish products (en%)	0.3 ±0.5	0.2 ±0.4
SFA from savoury snacks (en%)	0.2 ±0.3	0.2 ±0.3
SFA from nuts and seeds (en%)	0.3 ±0.5	0.2 ±0.4
Total TFA (en%)	0.5 ±0.2	0.5 ±0.2
Fibre (g/d)	18.3 ±6	17.5 ±5.6
Sodium (mg/d)	1,987 ±673	1,924 ±643
Dietary cholesterol (mg/d)	227 ±135	262 ±129
Fruit and vegetables (g/d)	387.6 ±230.3	380.5 ±221.7
Total dairy (g/d)	318.4 ±173.2	314.5 ±164
Milk (g/d)	230.5 ±153	229.6 ±143.9
Yogurt (g/d)	43.9 ±53.7	43.6 ±52.8
Cheese (g/d)	19.5 ±19.3	16.1 ±16.8
Total meat (g/d)	62.8 ±56.1	115.1 ±54.7
Unprocessed meat (g/d)	41.9 ±45.8	89.5 ±48.5
Processed meat (g/d)	18.3 ±26.2	23.9 ±29
Red meat (g/d)	60.3 ±54.2	51.1 ±43.6
Unprocessed red meat (g/d)	41.9 ±45.8	33.1 ±36.5
Processed red meat (g/d)	18.3 ±26.2	18.0 ±23.2
Poultry (g/d)	0 ±0	62.3 ±37
Unprocessed poultry (g/d)	0 ±0	56.4 ±37.8
Processed poultry (g/d)	0 ±0	5.9 ±17

**Data are presented as means ±SD for continuous variables or as % (n) for categorical variables, with % representing column percentages. Abbreviations. BMI: body mass index; CVD: cardiovascular diseases; en%: proportion (%) of total energy intake; METs, Metabolic equivalent of tasks; MUFA: monounsaturated fatty acids; PUFA: polyunsaturated fatty acids; SFA: saturated fatty acids; TFA: trans unsaturated fatty acids. †Females only*

Table S15. Cohort characteristics in non-consumers and consumers of saturated fatty acids intake from unprocessed poultry (percentage of total energy intake (%)) among 120,496 study participants in the UK Biobank*

	SFA from unprocessed poultry (en%)	
	**Zero consumers**	**Consumers (range: 0.1-5.5)**
N	60,029	60,467
**Sociodemographic factors**		
Female sex	56.6 (33,966)	57.4 (34,723)
Age (years)	56.2 (7.8)	55.6 (7.8)
White Europeans	96.6 (57,995)	96.5 (58,333)
College or university degree/vocation	57.9 (34,782)	56.1 (33,926)
Least deprived	21 (12,594)	22.7 (13,728)
Country of assessment centre		
England	91.5 (54,938)	91.6 (55,360)
Scotland	5.5 (3,276)	5.2 (3,154)
Wales	3 (1,815)	3.2 (1,953)
**Lifestyle factors**		
Current smoker	7.3 (4,392)	6.4 (3,867)
Physical activity (excess METs)		
Low <10	27.3 (16,359)	28.3 (17,118)
Moderate 10-50	52.9 (31,751)	53.3 (32,206)
High ≥50	17.8 (10,672)	16.5 (9,988)
Dietary supplement use	52.5 (31,537)	52.4 (31,673)
Alcohol intake (g/d)		
Non-drinker	6 (3,627)	5.2 (3,165)

	SFA from unprocessed poultry (en%)	
	Zero consumers	Consumers (range: 0.1-5.5)
<1	11 (6,614)	10.4 (6,274)
1-<10	26.6 (15,965)	27.2 (16,434)
10-<20	24.6 (14,786)	25.1 (15,171)
>=20	26 (15,605)	26.6 (16,112)
**Female factors**		
Hormone replacement therapy†	19.6 (11,744)	20.2 (12,197)
Menopausal status†	34.1 (20,440)	32.9 (19,865)
**Family history/cardiometabolic risk factors**		
Family history of CVD	56.1 (33,662)	56.3 (34,047)
BMI categories (kg/m^2)		
Underweight <18.5	0.7 (401)	0.5 (308)
Healthy weight 18.5-25	41 (24,639)	38.4 (23,230)
Overweight 25-30	39.9 (23,949)	41.2 (24,900)
Obesity 30<	18.2 (10,900)	19.7 (11,914)
Abdominal obesity	27 (16,233)	28.7 (17,358)
Baseline hypertension	24 (14,394)	24.3 (14,669)
Baseline hypercholesterolemia	12.8 (7,685)	13.1 (7,898)
Baseline diabetes mellitus	2.5 (1,496)	2.1 (1,264)
**Dietary intake**		
Total energy intake (kcal/d)	2,047 ±502	2,067 ±493
Total protein (en%)	15 ±2.8	16.8 ±3
Total carbohydrate (en%)	49.9 ±7.5	48.6 ±7.2
Total fat (en%)	32.2 ±5.9	31.4 ±5.6

	SFA from unprocessed poultry (en%)	
	Zero consumers	Consumers (range: 0.1-5.5)
Total MUFA (en%)	11.5 ±2.5	11.4 ±2.4
Total PUFA (en%)	5.6 ±1.6	5.7 ±1.5
Total SFA (en%)	12.0 ±3	11.4 ±2.8
SFA from total dairy (en%)	3.5 ±2.1	3.2 ±1.9
SFA from milk (en%)	0.9 ±0.8	0.9 ±0.7
SFA from yogurt (en%)	0.2 ±0.4	0.2 ±0.4
SFA from cheese (en%)	1.5 ±1.5	1.2 ±1.3
SFA from total meat (en%)	1.5 ±1.4	1.7 ±1.1
SFA from unprocessed meat (en%)	0.5 ±0.6	0.7 ±0.6
SFA from processed meat (en%)	0.5 ±0.7	0.4 ±0.6
SFA from red meat (en%)	0.8 ±0.8	0.7 ±0.7
SFA from unprocessed red meat (en%)	1.0 ±1.1	0.7 ±0.9
SFA from processed red meat (en%)	0.5 ±0.7	0.4 ±0.6
SFA from poultry (en%)	0 ±0.1	0.5 ±0.3
SFA from unprocessed poultry (en%)	0 ±0	0.5 ±0.3
SFA from processed poultry (en%)	0 ±0.1	0 ±0.1
SFA from cereal and cereal products (en%)	2.8 ±1.7	2.7 ±1.6
SFA from fat spreads (en%)	1.3 ±1.5	1.2 ±1.3
SFA from vegetables and vegetables dishes (en%)	0.8 ±0.7	0.7 ±0.6
SFA from sugar, sweet spreads, preserves (en%)		
SFA from eggs and egg products (en%)	0.4 ±0.7	0.4 ±0.6
SFA from soups, savoury sauces, samosa, pakora, sushi (en%)	0.4 ±0.4	0.4 ±0.3

	SFA from unprocessed poultry (en%)	
	Zero consumers	Consumers (range: 0.1-5.5)
SFA from fish and fish products (en%)	0.3 ±0.5	0.2 ±0.4
SFA from savoury snacks (en%)	0.2 ±0.3	0.2 ±0.3
SFA from nuts and seeds (en%)	0.3 ±0.5	0.2 ±0.4
Total TFA (en%)	0.5 ±0.2	0.5 ±0.2
Fibre (g/d)	18.2 ±6	17.5 ±5.6
Sodium (mg/d)	1,996 ±675	1,910 ±637
Dietary cholesterol (mg/d)	226 ±135	265 ±129
Fruit and vegetables (g/d)	383.1 ±229.3	384.4 ±222
Total dairy (g/d)	318 ±172.9	314.6 ±163.5
Milk (g/d)	230.6 ±152.7	229.5 ±143.5
Yogurt (g/d)	43.5 ±53.5	44 ±53
Cheese (g/d)	19.3 ±19.2	16 ±16.7
Total meat (g/d)	66.1 ±57.1	116.1 ±54.5
Unprocessed meat (g/d)	41.7 ±45.4	93.6 ±46.8
Processed meat (g/d)	22.0 ±29.8	20.7 ±25.8
Red meat (g/d)	60.2 ±53.6	50.4 ±43.2
Unprocessed red meat (g/d)	41.7 ±45.4	32.6 ±36.1
Processed red meat (g/d)	18.5 ±26.1	17.8 ±23
Poultry (g/d)	3.5 ±14	63.9 ±37.1
Unprocessed poultry (g/d)	0 ±0	61 ±35.5
Processed poultry (g/d)	3.5 ±14	2.9 ±11.7

**Data are presented as means ±SD for continuous variables or as % (n) for categorical variables, with % representing column percentages. Abbreviations. BMI: body mass index; CVD: cardiovascular diseases; en%: proportion (%) of total energy intake; METs, Metabolic equivalent of tasks; MUFA: monounsaturated fatty acids; PUFA: polyunsaturated fatty acids; SFA: saturated fatty acids; TFA: trans unsaturated fatty acid. †Females only*

Table S16. Cohort characteristics in non-consumers and consumers of saturated fatty acids intake from processed poultry (percentage of total energy intake (%)) among 120,496 study participants in the UK Biobank*

	SFA from processed poultry (en%)	
	**Zero consumers**	**Consumers (range: 0.0-3.7)**
N	110,627	9869
**Sociodemographic factors**		
Female sex	57.7 (63,785)	49.7 (4,904)
Age (years)	56 ±7.8	54.8 ±8.1
White Europeans	96.7 (107,012)	94.4 (9,316)
College or university degree/vocation	57.5 (63,595)	51.8 (5,113)
Least deprived	22 (24,344)	20 (1,978)
Country of assessment centre		
England	91.5 (101,262)	91.6 (9,036)
Scotland	5.3 (5,874)	5.6 (556)
Wales	3.2 (3,491)	2.8 (277)
**Lifestyle factors**		
Current smoker	6.8 (7,510)	7.6 (749)
Physical activity (excess METs)		
Low <10	27.6 (30,540)	29.8 (2,937)
Moderate 10-50	53.2 (58,891)	51.3 (5,066)
High ≥50	17.2 (18,984)	17 (1,676)
Dietary supplement use	52.7 (58,255)	50.2 (4,955)
Alcohol intake (g/d)		
Non-drinker	5.6 (6,148)	6.5 (644)

	SFA from processed poultry (en%)	
	Zero consumers	Consumers (range: 0.0-3.7)
<1	10.6 (11,723)	11.8 (1,165)
1-<10	27 (29,905)	25.3 (2,494)
10-<20	25.1 (27,727)	22.6 (2,230)
>=20	26.2 (28,945)	28.1 (2,772)
**Female factors**		
Hormone replacement therapy†	20.1 (22,271)	16.9 (1,670)
Menopausal status†	34.1 (37,696)	26.4 (2,609)
**Family history/cardiometabolic risk factors**		
Family history of CVD	56.3 (62,285)	55 (5,424)
BMI categories (kg/m^2)		
Underweight <18.5	0.6 (667)	0.4 (42)
Healthy weight 18.5-25	40.3 (44,635)	32.8 (3,234)
Overweight 25-30	40.3 (44,608)	43 (4,241)
Obesity 30<	18.5 (20,484)	23.6 (2,330)
Abdominal obesity	27.5 (30,418)	32.2 (3,173)
Baseline hypertension	24 (26,547)	25.5 (2,516)
Baseline hypercholesterolemia	12.9 (14,271)	13.3 (1,312)
Baseline diabetes mellitus	2.3 (2,504)	2.6 (256)
**Dietary intake**		
Total energy intake (kcal/d)	2,052 ±495	2,119 ±522
Total protein (en%)	15.9 ±3	15.5 ±2.8
Total carbohydrate (en%)	49.2 ±7.4	49.3 ±7
Total fat (en%)	31.7 ±5.8	32.2 ±5.5

	SFA from processed poultry (en%)	
	**Zero consumers**	**Consumers (range: 0.0-3.7)**
Total MUFA (en%)	11.4 ±2.4	12 ±2.3
Total PUFA (en%)	5.6 ±1.6	5.9 ±1.5
Total SFA (en%)	11.7 ±2.9	11.5 ±2.8
SFA from total dairy (en%)	3.4 ±2	3.2 ±1.9
SFA from milk (en%)	0.9 ±0.7	0.9 ±0.7
SFA from yogurt (en%)	0.2 ±0.4	0.2 ±0.3
SFA from cheese (en%)	1.4 ±1.4	1.2 ±1.2
SFA from total meat (en%)	1.6 ±1.3	1.8 ±1.1
SFA from unprocessed meat (en%)	0.6 ±0.6	0.6 ±0.6
SFA from processed meat (en%)	0.4 ±0.7	0.8 ±0.7
SFA from red meat (en%)	0.7 ±0.8	0.8 ±0.7
SFA from unprocessed red meat (en%)	0.8 ±1	0.7 ±0.9
SFA from processed red meat (en%)	0.4 ±0.7	0.4 ±0.7
SFA from poultry (en%)	0.3 ±0.3	0.6 ±0.4
SFA from unprocessed poultry (en%)	0.3 ±0.3	0.2 ±0.3
SFA from processed poultry (en%)	0 ±0	0.4 ±0.2
SFA from cereal and cereal products (en%)	2.7 ±1.6	2.7 ±1.6
SFA from fat spreads (en%)	1.2 ±1.4	1.1 ±1.3
SFA from vegetables and vegetables dishes (en%)	0.7 ±0.7	0.7 ±0.6
SFA from sugar, sweet spreads, preserves (en%)	0.5 ±0.9	0.6 ±0.9
SFA from eggs and egg products (en%)	0.4 ±0.7	0.3 ±0.6
SFA from soups, savoury sauces, samosa, pakora, sushi (en%)	0.4 ±0.4	0.3 ±0.3

	SFA from processed poultry (en%)	
	Zero consumers	Consumers (range: 0.0-3.7)
SFA from savoury snacks (en%)	0.2 ±0.3	0.2 ±0.3
SFA from nuts and seeds (en%)	0.2 ±0.4	0.2 ±0.4
Total TFA (en%)	0.5 ±0.2	0.5 ±0.2
Fibre (g/d)	18 ±5.8	17 ±5.6
Sodium (mg/d)	1,943 ±656	2,056 ±668
Dietary cholesterol (mg/d)	246 ±133	240 ±131
Fruit and vegetables (g/d)	387.7 ±226.5	339.7 ±211.1
Total dairy (g/d)	316.7 ±168.4	312 ±166.3
Milk (g/d)	229.9 ±148.3	231.6 ±146.5
Yogurt (g/d)	44.2 ±53.5	38.9 ±50
Cheese (g/d)	17.8 ±18.2	15.4 ±16.8
Total meat (g/d)	88.6 ±60.6	120.5 ±60.5
Unprocessed meat (g/d)	68.3 ±53.3	61.1 ±48.3
Processed meat (g/d)	18.1 ±24.7	57.7 ±35.1
Red meat (g/d)	55.5 ±49.2	53.1 ±45.7
Unprocessed red meat (g/d)	37.4 ±41.5	34.3 ±38.5
Processed red meat (g/d)	18.1 ±24.7	18.8 ±24
Poultry (g/d)	31 ±39.9	65.7 ±43.1
Unprocessed poultry (g/d)	31 ±39.9	26.8 ±35.1
Processed poultry (g/d)	0 ±0	38.9 ±25.3

**Data are presented as means ±SD for continuous variables or as % (n) for categorical variables, with % representing column percentages. Abbreviations. BMI: body mass index; CVD: cardiovascular diseases; en%: proportion (%) of total energy intake; METs, Metabolic equivalent of tasks; MUFA: monounsaturated fatty acids; PUFA: polyunsaturated fatty acids; SFA: saturated fatty acids; TFA: trans unsaturated fatty acids. †Females only*

Table S17. Hazard ratio (95% confidence intervals) for risk of cardiovascular diseases, coronary heart diseases and cerebrovascular diseases associated with substitution of 2.5% of energy of saturated fatty acids from total and subtypes of meats with 2.5% of energy of saturated fatty acids from total and subtypes of dairy, with adjustment for total energy and without adjustment for protein, carbohydrate, monounsaturated, polyunsaturated, saturated fatty acids from non-meat and non-dairy sources, trans fatty acids, and dietary cholesterol, the UK Biobank Study (n=120,496)

	Hazard ratio (95% confidence intervals)*		
	**Cardiovascular diseases**	**Coronary heart diseases**	**Cerebrovascular diseases**
**Total meat SFA (2.5 en%) substituted with:**			
Total dairy SFA	0.94 (0.90,0.99)	0.94 (0.89,0.99)	0.98 (0.90,1.05)
**Unprocessed meat SFA (2.5 en%) substituted with:**			
Total dairy SFA	1.00 (0.92,1.08)	1.01 (0.92,1.12)	1.02 (0.88,1.18)
Milk SFA	1.06 (0.96,1.18)	1.10 (0.97,1.24)	1.03 (0.86,1.23)
Yogurt SFA	0.91 (0.77,1.07)	1.00 (0.83,1.21)	0.78 (0.59,1.04)
Cheese SFA	0.98 (0.90,1.07)	0.98 (0.88,1.08)	1.04 (0.89,1.21)
**Processed meat SFA (2.5 en%) substituted with:**			
Total dairy SFA	0.90 (0.84,0.97)	0.89 (0.82,0.97)	0.94 (0.82,1.06)
Milk SFA	0.96 (0.87,1.05)	0.96 (0.86,1.08)	0.94 (0.80,1.12)
Yogurt SFA	0.82 (0.69,0.96)	0.87 (0.73,1.05)	0.72 (0.54,0.95)
Cheese SFA	0.88 (0.82,0.95)	0.86 (0.78,0.94)	0.95 (0.83,1.09)
**Red meat SFA (2.5 en%) substituted with:**			
Total dairy SFA	0.96 (0.89,1.02)	0.96 (0.89,1.05)	0.99 (0.87,1.11)
Milk SFA	1.02 (0.93,1.12)	1.04 (0.93,1.16)	0.99 (0.84,1.17)
Yogurt SFA	0.87 (0.74,1.02)	0.95 (0.79,1.14)	0.76 (0.57,1.00)
Cheese SFA	0.94 (0.87,1.01)	0.93 (0.85,1.01)	1.00 (0.88,1.14)

	Hazard ratio (95% confidence intervals)*		
	Cardiovascular diseases	Coronary heart diseases	Cerebrovascular diseases
**Unprocessed red meat SFA (2.5 en%) substituted with:**			
Total dairy SFA	0.95 (0.90,1.00)	0.95 (0.90,1.02)	0.96 (0.88,1.06)
Milk SFA	1.01 (0.93,1.10)	1.03 (0.94,1.14)	0.97 (0.84,1.12)
Yogurt SFA	0.86 (0.74,1.01)	0.94 (0.79,1.13)	0.74 (0.57,0.97)
Cheese SFA	0.93 (0.88,0.99)	0.92 (0.86,0.99)	0.98 (0.89,1.09)
**Processed red meat SFA (2.5 en%) substituted with:**			
Total dairy SFA	0.91 (0.84,0.98)	0.90 (0.83,0.98)	0.94 (0.83,1.07)
Milk SFA	0.96 (0.88,1.06)	0.97 (0.87,1.09)	0.95 (0.80,1.12)
Yogurt SFA	0.82 (0.70,0.97)	0.89 (0.73,1.07)	0.72 (0.55,0.96)
Cheese SFA	0.89 (0.82,0.96)	0.86 (0.79,0.95)	0.96 (0.84,1.10)
**Poultry SFA substituted (2.5 en%) with:**			
Total dairy SFA	1.00 (0.86,1.16)	0.94 (0.79,1.11)	1.26 (0.97,1.64)
Milk SFA	1.07 (0.91,1.26)	1.02 (0.85,1.23)	1.27 (0.95,1.69)
Yogurt SFA	0.91 (0.74,1.12)	0.93 (0.73,1.18)	0.97 (0.67,1.39)
Cheese SFA	0.98 (0.85,1.14)	0.91 (0.76,1.08)	1.28 (0.98,1.67)
**Unprocessed poultry SFA (2.5 en%) substituted with:**			
Total dairy SFA	1.06 (0.90,1.24)	0.99 (0.83,1.19)	1.32 (0.99,1.74)
Milk SFA	1.13 (0.95,1.34)	1.08 (0.89,1.31)	1.32 (0.98,1.78)
Yogurt SFA	0.96 (0.78,1.19)	0.98 (0.76,1.26)	1.01 (0.69,1.47)
Cheese SFA	1.04 (0.89,1.22)	0.96 (0.80,1.16)	1.34 (1.01,1.77)
**Processed poultry SFA (2.5 en%) substituted with:**			
Total dairy SFA	0.67 (0.45,0.98)	0.63 (0.41,0.98)	0.90 (0.44,1.85)

	Hazard ratio (95% confidence intervals)*		
	**Cardiovascular diseases**	**Coronary heart diseases**	**Cerebrovascular diseases**
**Processed poultry SFA (2.5 en%) substituted with:**			
Milk SFA	0.72 (0.49,1.06)	0.70 (0.44,1.09)	0.91 (0.44,1.89)
Yogurt SFA	0.61 (0.41,0.92)	0.63 (0.39,1.01)	0.69 (0.32,1.49)
Cheese SFA	0.66 (0.45,0.97)	0.62 (0.40,0.96)	0.92 (0.45,1.89)

**Hazard ratios (95% confidence intervals) were derived from Cox proportional hazard regression models, adjusted for sex (female/males), age (y), total energy intake (kcal/d), ethnic background (white Europeans/south Asians/African Caribbean/multiple ethnic background or other/unknown), education (college or university degree or vocation/national examination at 17-18 years of age/national examination at 16 years of age/unknown), Townsend deprivation index (quintiles), country of assessment centre (England/Scotland/Wales), smoking (current/former/never/unknown), physical activity (low/moderate/high/unknown), alcohol intake (non-drinkers/<1 g/d/1-10 g/d/10-20 g/d/≥20 g/d/unknown), dietary supplement use (yes/no), hormone replacement therapy use (females only) (yes/no/unknown), menopausal status (females only) (yes/no/not sure – had a hysterectomy/not sure -other reason/unknown), fruit and vegetables (g/d), fibre (g/d), family history of CVD (yes/no), BMI (underweight/healthy weight/overweight/obesity/unknown), abdominal obesity (yes/no/unknown), baseline hypertension (yes/no), baseline hypercholesterolemia (yes/no) and baseline diabetes (yes/no). Saturated fatty acids from individual food sources were mutually adjusted for each other in the analyses, as appropriate. Abbreviations. SFA: saturated fatty acids.*

Table S18. Hazard ratio (95% confidence intervals) for risk of cardiovascular diseases, coronary heart diseases and cerebrovascular diseases associated with substitution of 2.5% of energy of saturated fatty acids from a type of meat with 2.5% of energy of saturated fatty acids from another type of meat, with adjustment for total energy and without adjustment for protein, carbohydrate, monounsaturated, polyunsaturated, saturated fatty acids from non-meat sources, trans fatty acids, and dietary cholesterol, the UK Biobank Study (n=120,496)

	Hazard ratio (95% confidence intervals)*		
	**Cardiovascular diseases**	**Coronary heart diseases**	**Cerebrovascular diseases**
**Processed meat SFA (2.5 en%) substituted with:**			
Unprocessed meat SFA	0.90 (0.82,1.00)	0.88 (0.78,0.99)	0.92 (0.77,1.10)
**Processed red meat SFA (2.5 en%) substituted with:**			
Unprocessed red meat SFA	0.96 (0.88,1.04)	0.94 (0.86,1.04)	0.98 (0.84,1.13)
**Processed poultry SFA (2.5 en%) substituted with:**			
Unprocessed poultry SFA	0.63 (0.42,0.95)	0.64 (0.40,1.02)	0.68 (0.32,1.46)
**Red meat SFA (2.5 en%) substituted with:**			
Poultry SFA	0.97 (0.83,1.12)	1.04 (0.87,1.24)	0.79 (0.60,1.03)

**Hazard ratios (95% confidence intervals) were derived from Cox proportional hazard regression models, adjusted for sex (female/males), age (y), total energy intake (kcal/d), ethnic background (white Europeans/south Asians/African Caribbean/multiple ethnic background or other/unknown), education (college or university degree or vocation/national examination at 17-18 years of age/national examination at 16 years of age/unknown), Townsend deprivation index (quintiles), country of assessment centre (England/Scotland/Wales), smoking (current/former/never/unknown), physical activity (low/moderate/high/unknown), alcohol intake (non-drinkers/<1 g/d/1-10 g/d/10-20 g/d/≥20 g/d/unknown), dietary supplement use (yes/no), hormone replacement therapy use (females only) (yes/no/unknown), menopausal status (females only) (yes/no/not sure – had a hysterectomy/not sure -other reason/unknown), fruit and vegetables (g/d), fibre (g/d), family history of CVD (yes/no), BMI (underweight/healthy weight/overweight/obesity/unknown), abdominal obesity (yes/no/unknown), baseline hypertension (yes/no), baseline hypercholesterolemia (yes/no) and baseline diabetes (yes/no). Saturated fatty acids from individual food sources were mutually adjusted for each other in the analyses, as appropriate. Abbreviations. SFA: saturated fatty acids*

Table S19. Hazard ratio (95% confidence intervals) for risk of cardiovascular diseases, coronary heart diseases and cerebrovascular diseases associated with substitution of one serving of total and subtypes of meat with one serving of total and subtypes of dairy, additionally adjusted for total saturated fatty acids, UK Biobank Study (n=120,496)

	Hazard ratio (95% confidence intervals)*		
	**Cardiovascular diseases**	**Coronary heart diseases**	**Cerebrovascular diseases**
**Total meat (100 g) substituted with:**			
Total dairy (100 g)	0.93 (0.89,0.97)	0.92 (0.88,0.97)	0.97 (0.90,1.04)
**Unprocessed meat (100 g) substituted with:**			
Total dairy (100 g)	0.95 (0.90,1.00)	0.95 (0.90,1.01)	0.99 (0.90,1.08)
Milk (200 g)	0.96 (0.91,1.02)	0.96 (0.90,1.03)	1.00 (0.90,1.10)
Yogurt (125 g)	0.93 (0.87,1.00)	0.97 (0.90,1.05)	0.89 (0.79,1.01)
Cheese (30 g)	0.92 (0.87,0.98)	0.91 (0.85,0.97)	1.00 (0.90,1.10)
**Processed meat (50 g) substituted with:**			
Total dairy (100 g)	0.92 (0.89,0.96)	0.92 (0.88,0.96)	0.97 (0.91,1.04)
Milk (200 g)	0.93 (0.89,0.97)	0.92 (0.87,0.97)	0.98 (0.90,1.07)
Yogurt (125 g)	0.90 (0.85,0.96)	0.93 (0.87,1.00)	0.88 (0.79,0.98)
Cheese (30 g)	0.89 (0.85,0.94)	0.87 (0.82,0.92)	0.98 (0.90,1.07)
**Red meat (100 g) substituted with:**			
Total dairy (100 g)	0.90 (0.85,0.94)	0.90 (0.85,0.96)	0.94 (0.86,1.03)
Milk (200 g)	0.91 (0.86,0.96)	0.92 (0.86,0.98)	0.95 (0.86,1.05)

	Hazard ratio (95% confidence intervals)*		
	**Cardiovascular diseases**	**Coronary heart diseases**	**Cerebrovascular diseases**
Yogurt (125 g)	0.88 (0.83,0.95)	0.93 (0.85,1.00)	0.85 (0.75,0.96)
Cheese (30 g)	0.88 (0.83,0.93)	0.87 (0.81,0.93)	0.96 (0.86,1.06)
**Unprocessed red meat (100 g) substituted with:**			
Total dairy (100 g)	0.91 (0.86,0.97)	0.93 (0.87,1.00)	0.93 (0.83,1.03)
Milk (200 g)	0.93 (0.87,0.99)	0.95 (0.88,1.02)	0.94 (0.84,1.05)
Yogurt (125 g)	0.90 (0.84,0.97)	0.96 (0.88,1.05)	0.84 (0.73,0.96)
Cheese (30 g)	0.90 (0.84,0.96)	0.90 (0.83,0.97)	0.95 (0.84,1.06)
**Processed red meat (50 g) substituted with:**			
Total dairy (100 g)	0.93 (0.89,0.97)	0.92 (0.88,0.97)	0.98 (0.90,1.05)
Milk (200 g)	0.94 (0.89,0.98)	0.93 (0.88,0.98)	0.98 (0.90,1.08)
Yogurt (125 g)	0.91 (0.85,0.97)	0.94 (0.87,1.01)	0.88 (0.79,0.99)
Cheese (30 g)	0.90 (0.86,0.95)	0.88 (0.82,0.93)	0.99 (0.90,1.09)
**Poultry (100 g) substituted with:**			
Total dairy (100 g)	0.96 (0.91,1.02)	0.94 (0.88,1.01)	1.04 (0.94,1.15)
Milk (200 g)	0.97 (0.91,1.03)	0.95 (0.88,1.02)	1.04 (0.93,1.17)
Yogurt (125 g)	0.94 (0.87,1.01)	0.96 (0.88,1.04)	0.94 (0.82,1.07)
Cheese (30 g)	0.93 (0.87,1.00)	0.90 (0.83,0.97)	1.05 (0.93,1.18)
**Unprocessed poultry (100 g) substituted with:**			
Total dairy (100 g)	0.98 (0.92,1.04)	0.96 (0.90,1.03)	1.05 (0.94,1.17)
Milk (200 g)	0.98 (0.92,1.05)	0.97 (0.90,1.05)	1.06 (0.94,1.19)
Yogurt (125 g)	0.96 (0.89,1.03)	0.98 (0.90,1.07)	0.95 (0.83,1.08)
Cheese (30 g)	0.95 (0.89,1.02)	0.92 (0.85,0.99)	1.06 (0.94,1.20)

	Hazard ratio (95% confidence intervals)*		
	**Cardiovascular diseases**	**Coronary heart diseases**	**Cerebrovascular diseases**
**Processed poultry (50 g) substituted with:**			
Total dairy (100 g)	0.91 (0.85,0.98)	0.91 (0.83,0.98)	0.97 (0.84,1.12)
Milk (200 g)	0.92 (0.85,0.99)	0.91 (0.83,0.99)	0.98 (0.84,1.13)
Yogurt (125 g)	0.89 (0.81,0.97)	0.92 (0.83,1.01)	0.88 (0.74,1.03)
Cheese (30 g)	0.88 (0.81,0.96)	0.86 (0.78,0.94)	0.98 (0.84,1.15)

**Hazard ratios (95% confidence intervals) were derived from Cox proportional hazard regression models, adjusted for sex (female/males), age (y), ethnic background (white Europeans/south Asians/African Caribbean/multiple ethnic background or other/unknown), education (college or university degree or vocation/national examination at 17-18 years of age/national examination at 16 years of age/unknown), Townsend deprivation index (quintiles), country of assessment centre (England/Scotland/Wales), smoking (current/former/never/unknown), physical activity (low/moderate/high/unknown), alcohol intake (non- drinkers/<1 g/d/1-10 g/d/10-20 g/d/≥20 g/d/unknown), dietary supplement use (yes/no), hormone replacement therapy use (females only) (yes/no/unknown), menopausal status (females only) (yes/no/not sure – had a hysterectomy/not sure -other reason/unknown), fruit and vegetables (g/d), fibre (g/d), family history of CVD (yes/no), BMI (underweight/healthy weight/overweight/obesity/unknown), abdominal obesity (yes/no/unknown), baseline hypertension (yes/no), baseline hypercholesterolemia (yes/no) and baseline diabetes (yes/no), trans unsaturated fatty acids (en%), dietary cholesterol (mg/d), protein (en%), carbohydrate (en%), monounsaturated fatty acids (en%), polyunsaturated fatty acids (en%), saturated fatty acids (en%). Dairy and meat sources were mutually adjusted for each other in the analyses, as appropriate. Abbreviations. SFA: saturated fatty acids.*

Table S20. Hazard ratio (95% confidence intervals) for risk of cardiovascular diseases, coronary heart diseases and cerebrovascular diseases associated with substitution of one serving of a type of meat with another, additionally adjusted for total saturated fatty acids, UK Biobank Study (n=120,496)

	Hazard ratio (95% confidence intervals)*		
	**Cardiovascular diseases**	**Coronary heart diseases**	**Cerebrovascular diseases**
**Processed meat (50 g) substituted with:**			
Unprocessed meat (100 g)	0.98 (0.92,1.03)	0.97 (0.91,1.03)	0.99 (0.90,1.09)
**Processed red meat (50 g) substituted with:**			
Unprocessed red meat (100 g)	1.02 (0.95,1.09)	0.99 (0.92,1.07)	1.05 (0.93,1.19)
**Processed poultry (50 g) substituted with:**			
Unprocessed poultry (100 g)	0.93 (0.85,1.02)	0.94 (0.85,1.04)	0.93 (0.78,1.10)
**Red meat (100 g) substituted with:**			
Poultry (100 g)	0.94 (0.88,0.99)	0.96 (0.89,1.03)	0.90 (0.81,1.01)

**Hazard ratios (95% confidence intervals) were derived from Cox proportional hazard regression models, adjusted for sex (female/males), age (y), ethnic background (white Europeans/south Asians/African Caribbean/multiple ethnic background or other/unknown), education (college or university degree or vocation/national examination at 17-18 years of age/national examination at 16 years of age/unknown), Townsend deprivation index (quintiles), country of assessment centre (England/Scotland/Wales), smoking (current/former/never/unknown), physical activity (low/moderate/high/unknown), alcohol intake (non- drinkers/<1 g/d/1-10 g/d/10-20 g/d/≥20 g/d/unknown), dietary supplement use (yes/no), hormone replacement therapy use (females only) (yes/no/unknown), menopausal status (females only) (yes/no/not sure – had a hysterectomy/not sure -other reason/unknown), fruit and vegetables (g/d), fibre (g/d), family history of CVD (yes/no), BMI (underweight/healthy weight/overweight/obesity/unknown), abdominal obesity (yes/no/unknown), baseline hypertension (yes/no), baseline hypercholesterolemia (yes/no) and baseline diabetes (yes/no), trans unsaturated fatty acids (en%), dietary cholesterol (mg/d), protein (en%), carbohydrate (en%), monounsaturated fatty acids (en%), polyunsaturated fatty acids (en%), saturated fatty acids (en%). Meat sources were mutually adjusted for each other in the analyses, as appropriate. Abbreviations. SFA: saturated fatty acids.*

Table S21. Hazard ratios (95% confidence intervals) for risk of cardiovascular diseases, coronary heart diseases and cerebrovascular diseases associated with substitution of saturated fatty acids from total and subtypes of meat with saturated fatty acids from total and subtypes of dairy among UK Biobank participants (n=120,496): Sensitivity analyses

	Hazard ratio (95% confidence intervals)*		
	Cardiovascular diseases	Coronary heart diseases	Stroke
**Substitution of SFA from total meat with total dairy (2.5 en%)**			
Overall (main result)	0.91 (0.86,0.96)	0.92 (0.86,0.98)	0.91 (0.83,1.00)
(1) Age as underlying timescale	0.91 (0.86,0.96)	0.92 (0.86,0.98)	0.91 (0.83,1.00)
(2) Exclusion of first two years of follow-up (n=1,417)	0.89 (0.84,0.95)	0.89 (0.83,0.96)	0.92 (0.83,1.02)
(3) Exclusion of prevalent diabetes and hypercholesterolemia (n=16,165)	0.89 (0.84,0.95)	0.90 (0.83,0.97)	0.90 (0.81,1.01)
(4) Exclusion of ill/fasting participants (n=3,351)	0.90 (0.85,0.95)	0.91 (0.85,0.97)	0.91 (0.82,1.00)
(5) Restriction to ≥three 24hr-dietary assessments (n=74,934)	0.88 (0.81,0.94)	0.86 (0.79,0.94)	0.94 (0.82,1.07)
(6) Exclusion of ethnic minority groups (n=4168)	0.90 (0.85,0.96)	0.92 (0.86,0.98)	0.90 (0.81,0.99)
**Substitution of SFA from unprocessed meat with total dairy (2.5 en%)**			
Overall (main result)	1.01 (0.91,1.13)	1.07 (0.94,1.21)	0.97 (0.80,1.17)
(1) Age as underlying timescale	1.03 (0.92,1.14)	1.08 (0.95,1.23)	0.99 (0.82,1.19)
(2) Exclusion of first two years of follow-up (n=1,417)	0.97 (0.86,1.09)	1.00 (0.87,1.15)	0.99 (0.81,1.21)
(3) Exclusion of prevalent diabetes and hypercholesterolemia (n=16,165)	1.01 (0.89,1.15)	1.08 (0.93,1.26)	0.91 (0.73,1.13)
(4) Exclusion of ill/fasting participants (n=3,351)	1.01 (0.90,1.13)	1.06 (0.93,1.21)	0.97 (0.80,1.18)
(5) Restriction to ≥three 24hr-dietary assessments (n=74,934)	0.93 (0.81,1.06)	0.94 (0.80,1.11)	0.94 (0.74,1.19)
(6) Exclusion of ethnic minority groups (n=4168)	0.99 (0.89,1.11)	1.04 (0.92,1.19)	0.95 (0.79,1.15)
**Substitution of SFA from unprocessed meat with milk (2.5 en%)**			
Overall (main result)	1.07 (0.94,1.21)	1.15 (0.99,1.33)	0.98 (0.79,1.23)
(1) Age as underlying timescale	1.08 (0.95,1.23)	1.16 (1.00,1.35)	1.00 (0.80,1.25)
(2) Exclusion of first two years of follow-up (n=1,417)	1.02 (0.89,1.18)	1.08 (0.91,1.27)	0.98 (0.77,1.24)
(3) Exclusion of prevalent diabetes and hypercholesterolemia (n=16,165)	1.08 (0.93,1.25)	1.17 (0.98,1.40)	0.95 (0.73,1.23)
(4) Exclusion of ill/fasting participants (n=3,351)	1.07 (0.94,1.22)	1.14 (0.98,1.33)	0.98 (0.78,1.23)
(5) Restriction to ≥three 24hr-dietary assessments (n=74,934)	0.96 (0.82,1.14)	1.00 (0.82,1.21)	0.95 (0.71,1.26)
(6) Exclusion of ethnic minority groups (n=4168)	1.05 (0.92,1.19)	1.12 (0.97,1.31)	0.96 (0.77,1.21)
**Substitution of SFA from unprocessed meat with yogurt (2.5 en%)**			
Overall (main result)	0.92 (0.77,1.11)	1.05 (0.85,1.30)	0.77 (0.56,1.05)
(1) Age as underlying timescale	0.93 (0.78,1.11)	1.06 (0.86,1.31)	0.77 (0.56,1.06)
(2) Exclusion of first two years of follow-up (n=1,417)	0.93 (0.76,1.13)	1.04 (0.82,1.30)	0.83 (0.59,1.15)
(3) Exclusion of prevalent diabetes and hypercholesterolemia (n=16,165)	0.90 (0.73,1.11)	1.04 (0.81,1.33)	0.70 (0.49,1.01)
(4) Exclusion of ill/fasting participants (n=3,351)	0.91 (0.76,1.10)	1.05 (0.85,1.30)	0.75 (0.55,1.04)
(5) Restriction to ≥three 24hr-dietary assessments (n=74,934)	0.81 (0.63,1.03)	0.86 (0.65,1.15)	0.74 (0.49,1.12)
(6) Exclusion of ethnic minority groups (n=4168)	0.89 (0.74,1.07)	1.01 (0.82,1.26)	0.73 (0.53,1.01)
**Substitution of SFA from unprocessed meat with cheese (2.5 en%)**			
Overall (main result)	1.02 (0.91,1.13)	1.06 (0.94,1.21)	0.99 (0.82,1.20)
(1) Age as underlying timescale	1.03 (0.92,1.15)	1.08 (0.95,1.22)	1.01 (0.84,1.22)

	Hazard ratio (95% confidence intervals)*		
	Cardiovascular diseases	Coronary heart diseases	Stroke
(2) Exclusion of first two years of follow-up (n=1,417)	0.97 (0.86,1.09)	0.99 (0.86,1.14)	1.01 (0.82,1.23)
(3) Exclusion of prevalent diabetes and hypercholesterolemia (n=16,165)	1.01 (0.89,1.15)	1.08 (0.93,1.26)	0.93 (0.74,1.16)
(4) Exclusion of ill/fasting participants (n=3,351)	1.01 (0.90,1.13)	1.05 (0.92,1.20)	1.00 (0.82,1.21)
(5) Restriction to ≥three 24hr-dietary assessments (n=74,934)	0.94 (0.82,1.08)	0.95 (0.81,1.12)	0.96 (0.76,1.22)
(6) Exclusion of ethnic minority groups (n=4168)	1.00 (0.89,1.11)	1.04 (0.91,1.19)	0.97 (0.80,1.18)
**Substitution of SFA from processed meat with total dairy (2.5 en%)**			
Overall (main result)	0.88 (0.81,0.95)	0.87 (0.80, 0.96)	0.90 (0.78, 1.02)
(1) Age as underlying timescale	0.88 (0.81,0.95)	0.88 (0.80,0.96)	0.89 (0.78,1.02)
(2) Exclusion of first two years of follow-up (n=1,417)	0.86 (0.80,0.94)	0.85 (0.77,0.93)	0.92 (0.80,1.07)
(3) Exclusion of prevalent diabetes and hypercholesterolemia (n=16,165)	0.85 (0.78,0.94)	0.85 (0.76,0.95)	0.87 (0.74,1.02)
(4) Exclusion of ill/fasting participants (n=3,351)	0.87 (0.80,0.94)	0.87 (0.80,0.95)	0.88 (0.77,1.01)
(5) Restriction to ≥three 24hr-dietary assessments (n=74,934)	0.86 (0.77,0.95)	0.86 (0.76,0.97)	0.89 (0.74,1.07)
(6) Exclusion of ethnic minority groups (n=4168)	0.88 (0.82,0.95)	0.88 (0.81,0.97)	0.89 (0.78,1.03)
**Substitution of SFA from processed meat with milk (2.5 en%)**			
Overall (main result)	0.91 (0.82,1.00)	0.91 (0.81,1.02)	0.91 (0.76,1.08)
(1) Age as underlying timescale	0.91 (0.82,1.00)	0.91 (0.81,1.03)	0.91 (0.76,1.08)
(2) Exclusion of first two years of follow-up (n=1,417)	0.90 (0.81,1.00)	0.89 (0.78,1.01)	0.92 (0.76,1.11)
(3) Exclusion of prevalent diabetes and hypercholesterolemia (n=16,165)	0.89 (0.79,1.01)	0.90 (0.78,1.03)	0.89 (0.72,1.09)
(4) Exclusion of ill/fasting participants (n=3,351)	0.91 (0.82,1.00)	0.91 (0.81,1.03)	0.89 (0.74,1.06)
(5) Restriction to ≥three 24hr-dietary assessments (n=74,934)	0.88 (0.77,1.01)	0.89 (0.76,1.05)	0.89 (0.70,1.13)
(6) Exclusion of ethnic minority groups (n=4168)	0.91 (0.82,1.01)	0.92 (0.82,1.04)	0.90 (0.75,1.08)
**Substitution of SFA from processed meat with yogurt (2.5 en%)**			
Overall (main result)	0.78 (0.67,0.92)	0.84 (0.69,1.01)	0.71 (0.53,0.94)
(1) Age as underlying timescale	0.78 (0.66,0.92)	0.84 (0.69,1.01)	0.70 (0.52,0.93)
(2) Exclusion of first two years of follow-up (n=1,417)	0.81 (0.68,0.97)	0.86 (0.70,1.05)	0.77 (0.57,1.05)
(3) Exclusion of prevalent diabetes and hypercholesterolemia (n=16,165)	0.75 (0.62,0.90)	0.79 (0.63,0.99)	0.66 (0.47,0.91)
(4) Exclusion of ill/fasting participants (n=3,351)	0.77 (0.65,0.91)	0.84 (0.69,1.02)	0.68 (0.51,0.91)
(5) Restriction to ≥three 24hr-dietary assessments (n=74,934)	0.74 (0.59,0.92)	0.77 (0.59,1.01)	0.70 (0.47,1.02)
(6) Exclusion of ethnic minority groups (n=4168)	0.77 (0.65,0.91)	0.83 (0.68,1.01)	0.69 (0.51,0.92)
**Substitution of SFA from processed meat with cheese (2.5 en%)**			
Overall (main result)	0.86 (0.80,0.94)	0.85 (0.77,0.93)	0.91 (0.79,1.05)
(1) Age as underlying timescale	0.86 (0.80,0.94)	0.85 (0.77,0.93)	0.91 (0.79,1.05)
(2) Exclusion of first two years of follow-up (n=1,417)	0.85 (0.78,0.93)	0.82 (0.74,0.91)	0.94 (0.81,1.10)
(3) Exclusion of prevalent diabetes and hypercholesterolemia (n=16,165)	0.84 (0.76,0.92)	0.83 (0.74,0.93)	0.87 (0.73,1.03)
(4) Exclusion of ill/fasting participants (n=3,351)	0.86 (0.79,0.93)	0.84 (0.76,0.92)	0.90 (0.78,1.04)
(5) Restriction to ≥three 24hr-dietary assessments (n=74,934)	0.86 (0.77,0.96)	0.85 (0.75,0.97)	0.91 (0.74,1.11)
(6) Exclusion of ethnic minority groups (n=4168)	0.87 (0.80,0.94)	0.85 (0.77,0.94)	0.91 (0.79,1.06)
**Substitution of SFA from red meat with total dairy (2.5 en%)**			
Overall (main result)	0.95 (0.87, 1.03)	0.97 (0.88, 1.07)	0.95 (0.82, 1.10)

	Hazard ratio (95% confidence intervals)*		
	Cardiovascular diseases	Coronary heart diseases	Stroke
(1) Age as underlying timescale	0.96 (0.88,1.04)	0.98 (0.89,1.08)	0.96 (0.83,1.11)
(2) Exclusion of first two years of follow-up (n=1,417)	0.92 (0.84,1.01)	0.92 (0.83,1.02)	0.99 (0.84,1.16)
(3) Exclusion of prevalent diabetes and hypercholesterolemia (n=16,165)	0.94 (0.85,1.04)	0.97 (0.86,1.10)	0.91 (0.76,1.08)
(4) Exclusion of ill/fasting participants (n=3,351)	0.94 (0.86,1.03)	0.97 (0.88,1.07)	0.94 (0.81,1.09)
(5) Restriction to ≥three 24hr-dietary assessments (n=74,934)	0.89 (0.80,0.98)	0.89 (0.79,1.01)	0.92 (0.77,1.10)
(6) Exclusion of ethnic minority groups (n=4168)	0.94 (0.86,1.02)	0.96 (0.87,1.06)	0.94 (0.81,1.09)
**Substitution of SFA from red meat with milk (2.5 en%)**			
Overall (main result)	0.99 (0.89,1.10)	1.03 (0.91,1.17)	0.96 (0.80,1.16)
(1) Age as underlying timescale	1.00 (0.90,1.11)	1.04 (0.92,1.18)	0.98 (0.81,1.18)
(2) Exclusion of first two years of follow-up (n=1,417)	0.97 (0.86,1.09)	0.98 (0.86,1.12)	0.98 (0.80,1.20)
(3) Exclusion of prevalent diabetes and hypercholesterolemia (n=16,165)	1.00 (0.88,1.13)	1.04 (0.89,1.21)	0.94 (0.75,1.17)
(4) Exclusion of ill/fasting participants (n=3,351)	0.99 (0.89,1.11)	1.03 (0.91,1.17)	0.95 (0.78,1.15)
(5) Restriction to ≥three 24hr-dietary assessments (n=74,934)	0.92 (0.80,1.05)	0.94 (0.80,1.10)	0.93 (0.73,1.17)
(6) Exclusion of ethnic minority groups (n=4168)	0.98 (0.88,1.10)	1.02 (0.90,1.16)	0.95 (0.79,1.15)
**Substitution of SFA from red meat with yogurt (2.5 en%)**			
Overall (main result)	0.85 (0.72,1.01)	0.94 (0.78,1.15)	0.75 (0.56,1.00)
(1) Age as underlying timescale	0.86 (0.73,1.01)	0.95 (0.78,1.15)	0.75 (0.56,1.00)
(2) Exclusion of first two years of follow-up (n=1,417)	0.87 (0.73,1.04)	0.94 (0.76,1.16)	0.82 (0.60,1.12)
(3) Exclusion of prevalent diabetes and hypercholesterolemia (n=16,165)	0.83 (0.68,1.01)	0.92 (0.73,1.15)	0.69 (0.49,0.96)
(4) Exclusion of ill/fasting participants (n=3,351)	0.84 (0.71,1.00)	0.95 (0.78,1.15)	0.72 (0.54,0.97)
(5) Restriction to ≥three 24hr-dietary assessments (n=74,934)	0.77 (0.61,0.96)	0.81 (0.62,1.05)	0.72 (0.49,1.05)
(6) Exclusion of ethnic minority groups (n=4168)	0.83 (0.70,0.98)	0.92 (0.75,1.12)	0.72 (0.54,0.97)
**Substitution of SFA from red meat with cheese (2.5 en%)**			
Overall (main result)	0.94 (0.87,1.03)	0.96 (0.87,1.06)	0.97 (0.83,1.12)
(1) Age as underlying timescale	0.95 (0.88,1.04)	0.97 (0.88,1.07)	0.98 (0.85,1.14)
(2) Exclusion of first two years of follow-up (n=1,417)	0.91 (0.83,1.00)	0.90 (0.81,1.01)	1.01 (0.86,1.18)
(3) Exclusion of prevalent diabetes and hypercholesterolemia (n=16,165)	0.94 (0.85,1.04)	0.96 (0.85,1.09)	0.92 (0.77,1.10)
(4) Exclusion of ill/fasting participants (n=3,351)	0.94 (0.86,1.03)	0.95 (0.86,1.06)	0.96 (0.83,1.12)
(5) Restriction to ≥three 24hr-dietary assessments (n=74,934)	0.89 (0.80,1.00)	0.90 (0.79,1.02)	0.94 (0.78,1.13)
(6) Exclusion of ethnic minority groups (n=4168)	0.94 (0.86,1.02)	0.95 (0.86,1.05)	0.96 (0.83,1.12)
**Substitution of SFA from unprocessed red meat with total dairy (2.5 en%)**			
Overall (main result)	0.91 (0.85,0.98)	0.94 (0.86, 1.06)	0.89 (0.79, 1.01)
(1) Age as underlying timescale	0.91 (0.85,0.98)	0.94 (0.86,1.02)	0.89 (0.79,1.01)
(2) Exclusion of first two years of follow-up (n=1,417)	0.89 (0.82,0.96)	0.92 (0.84,1.00)	0.89 (0.78,1.02)
(3) Exclusion of prevalent diabetes and hypercholesterolemia (n=16,165)	0.90 (0.83,0.98)	0.93 (0.84,1.03)	0.88 (0.76,1.01)
(4) Exclusion of ill/fasting participants (n=3,351)	0.91 (0.84,0.98)	0.93 (0.86,1.01)	0.90 (0.79,1.02)
(5) Restriction to ≥three 24hr-dietary assessments (n=74,934)	0.88 (0.79,0.97)	0.87 (0.78,0.98)	0.93 (0.78,1.11)
(6) Exclusion of ethnic minority groups (n=4168)	0.90 (0.83,0.96)	0.93 (0.85,1.01)	0.87 (0.77,0.99)
**Substitution of SFA from unprocessed red meat with milk (2.5 en%)**			

	Hazard ratio (95% confidence intervals)*		
	Cardiovascular diseases	Coronary heart diseases	Stroke
Overall (main result)	0.95 (0.86,1.05)	1.00 (0.89,1.12)	0.90 (0.75,1.06)
(1) Age as underlying timescale	0.95 (0.86,1.05)	0.99 (0.89,1.11)	0.89 (0.75,1.06)
(2) Exclusion of first two years of follow-up (n=1,417)	0.93 (0.84,1.04)	0.98 (0.86,1.11)	0.88 (0.73,1.05)
(3) Exclusion of prevalent diabetes and hypercholesterolemia (n=16,165)	0.95 (0.85,1.07)	0.99 (0.86,1.14)	0.91 (0.74,1.10)
(4) Exclusion of ill/fasting participants (n=3,351)	0.95 (0.86,1.05)	1.00 (0.89,1.12)	0.90 (0.75,1.07)
(5) Restriction to ≥three 24hr-dietary assessments (n=74,934)	0.90 (0.79,1.04)	0.91 (0.78,1.07)	0.93 (0.74,1.18)
(6) Exclusion of ethnic minority groups (n=4168)	0.94 (0.85,1.04)	0.99 (0.88,1.11)	0.87 (0.73,1.03)
**Substitution of SFA from unprocessed red meat with yogurt (2.5 en%)**			
Overall (main result)	0.82 (0.70,0.97)	0.92 (0.76,1.11)	0.70 (0.53,0.93)
(1) Age as underlying timescale	0.82 (0.70,0.96)	0.91 (0.76,1.10)	0.69 (0.52,0.91)
(2) Exclusion of first two years of follow-up (n=1,417)	0.85 (0.71,1.01)	0.94 (0.77,1.16)	0.74 (0.55,0.99)
(3) Exclusion of prevalent diabetes and hypercholesterolemia (n=16,165)	0.80 (0.66,0.96)	0.88 (0.71,1.10)	0.67 (0.49,0.92)
(4) Exclusion of ill/fasting participants (n=3,351)	0.82 (0.69,0.96)	0.92 (0.76,1.11)	0.69 (0.52,0.92)
(5) Restriction to ≥three 24hr-dietary assessments (n=74,934)	0.76 (0.61,0.95)	0.79 (0.61,1.03)	0.73 (0.50,1.06)
(6) Exclusion of ethnic minority groups (n=4168)	0.80 (0.67,0.94)	0.89 (0.74,1.08)	0.66 (0.50,0.88)
**Substitution of SFA from unprocessed red meat with cheese (2.5 en%)**			
Overall (main result)	0.91 (0.85,0.98)	0.93 (0.86,1.01)	0.91 (0.80,1.03)
(1) Age as underlying timescale	0.91 (0.85,0.98)	0.93 (0.85,1.01)	0.91 (0.80,1.04)
(2) Exclusion of first two years of follow-up (n=1,417)	0.89 (0.82,0.96)	0.90 (0.82,0.99)	0.91 (0.80,1.04)
(3) Exclusion of prevalent diabetes and hypercholesterolemia (n=16,165)	0.90 (0.83,0.98)	0.93 (0.84,1.03)	0.89 (0.77,1.04)
(4) Exclusion of ill/fasting participants (n=3,351)	0.91 (0.84,0.98)	0.92 (0.85,1.01)	0.92 (0.81,1.04)
(5) Restriction to ≥three 24hr-dietary assessments (n=74,934)	0.89 (0.80,0.98)	0.88 (0.78,0.99)	0.95 (0.80,1.14)
(6) Exclusion of ethnic minority groups (n=4168)	0.90 (0.84,0.97)	0.92 (0.84,1.00)	0.89 (0.78,1.01)
**Substitution of SFA from processed red meat with total dairy (2.5 en%)**			
Overall (main result)	0.88 (0.82, 0.96)	0.88 (0.81, 0.97)	0.91 (0.79, 1.04)
(1) Age as underlying timescale	0.89 (0.82,0.96)	0.89 (0.81,0.97)	0.90 (0.79,1.04)
(2) Exclusion of first two years of follow-up (n=1,417)	0.88 (0.81,0.95)	0.86 (0.78,0.94)	0.94 (0.81,1.08)
(3) Exclusion of prevalent diabetes and hypercholesterolemia (n=16,165)	0.86 (0.79,0.95)	0.86 (0.77,0.95)	0.89 (0.75,1.04)
(4) Exclusion of ill/fasting participants (n=3,351)	0.88 (0.81,0.95)	0.88 (0.80,0.96)	0.89 (0.77,1.02)
(5) Restriction to ≥three 24hr-dietary assessments (n=74,934)	0.87 (0.78,0.97)	0.86 (0.76,0.98)	0.91 (0.75,1.10)
(6) Exclusion of ethnic minority groups (n=4168)	0.89 (0.82,0.96)	0.89 (0.81,0.98)	0.90 (0.79,1.04)
**Substitution of SFA from processed red meat with milk (2.5 en%)**			
Overall (main result)	0.92 (0.83,1.02)	0.92 (0.82,1.04)	0.92 (0.77,1.09)
(1) Age as underlying timescale	0.92 (0.83,1.02)	0.92 (0.82,1.04)	0.91 (0.76,1.09)
(2) Exclusion of first two years of follow-up (n=1,417)	0.91 (0.82,1.02)	0.90 (0.79,1.03)	0.93 (0.77,1.12)
(3) Exclusion of prevalent diabetes and hypercholesterolemia (n=16,165)	0.90 (0.80,1.02)	0.90 (0.78,1.04)	0.90 (0.73,1.11)
(4) Exclusion of ill/fasting participants (n=3,351)	0.91 (0.83,1.01)	0.92 (0.82,1.04)	0.89 (0.75,1.07)
(5) Restriction to ≥three 24hr-dietary assessments (n=74,934)	0.89 (0.77,1.02)	0.90 (0.76,1.05)	0.91 (0.72,1.16)
(6) Exclusion of ethnic minority groups (n=4168)	0.92 (0.83,1.02)	0.93 (0.83,1.05)	0.91 (0.76,1.09)

	Hazard ratio (95% confidence intervals)*		
	Cardiovascular diseases	Coronary heart diseases	Stroke
**Substitution of SFA from processed red meat with yogurt (2.5 en%)**			
Overall (main result)	0.80 (0.68,0.94)	0.85 (0.70,1.03)	0.72 (0.54,0.95)
(1) Age as underlying timescale	0.79 (0.67,0.93)	0.85 (0.70,1.03)	0.70 (0.53,0.94)
(2) Exclusion of first two years of follow-up (n=1,417)	0.83 (0.69,0.99)	0.87 (0.71,1.07)	0.78 (0.58,1.06)
(3) Exclusion of prevalent diabetes and hypercholesterolemia (n=16,165)	0.76 (0.63,0.92)	0.80 (0.64,1.00)	0.67 (0.48,0.92)
(4) Exclusion of ill/fasting participants (n=3,351)	0.78 (0.66,0.92)	0.85 (0.70,1.03)	0.69 (0.51,0.92)
(5) Restriction to ≥three 24hr-dietary assessments (n=74,934)	0.75 (0.60,0.93)	0.78 (0.60,1.01)	0.71 (0.48,1.04)
(6) Exclusion of ethnic minority groups (n=4168)	0.78 (0.66,0.93)	0.84 (0.69,1.02)	0.69 (0.52,0.93)
**Substitution of SFA from processed red meat with cheese (2.5 en%)**			
Overall (main result)	0.88 (0.81,0.96)	0.86 (0.78,0.95)	0.93 (0.81,1.08)
(1) Age as underlying timescale	0.88 (0.81,0.96)	0.86 (0.78,0.95)	0.93 (0.81,1.08)
(2) Exclusion of first two years of follow-up (n=1,417)	0.87 (0.79,0.95)	0.83 (0.75,0.92)	0.96 (0.83,1.13)
(3) Exclusion of prevalent diabetes and hypercholesterolemia (n=16,165)	0.86 (0.78,0.95)	0.84 (0.75,0.95)	0.89 (0.75,1.05)
(4) Exclusion of ill/fasting participants (n=3,351)	0.87 (0.80,0.95)	0.85 (0.77,0.94)	0.92 (0.79,1.06)
(5) Restriction to ≥three 24hr-dietary assessments (n=74,934)	0.87 (0.78,0.98)	0.86 (0.75,0.99)	0.93 (0.76,1.14)
(6) Exclusion of ethnic minority groups (n=4168)	0.89 (0.82,0.96)	0.87 (0.79,0.96)	0.93 (0.80,1.08)
**Substitution of SFA from poultry with total dairy (2.5 en%)**			
Overall (main result)	0.97 (0.82, 1.15)	0.95 (0.78,1.15)	1.11 (0.82, 1.49)
(1) Age as underlying timescale	0.97 (0.82,1.15)	0.94 (0.78,1.15)	1.11 (0.82,1.49)
(2) Exclusion of first two years of follow-up (n=1,417)	0.97 (0.81,1.16)	0.91 (0.73,1.12)	1.22 (0.89,1.68)
(3) Exclusion of prevalent diabetes and hypercholesterolemia (n=16,165)	0.92 (0.76,1.13)	0.91 (0.72,1.14)	1.03 (0.73,1.46)
(4) Exclusion of ill/fasting participants (n=3,351)	0.95 (0.80,1.12)	0.92 (0.76,1.12)	1.10 (0.82,1.49)
(5) Restriction to ≥three 24hr-dietary assessments (n=74,934)	1.02 (0.80,1.29)	1.05 (0.79,1.40)	1.09 (0.72,1.67)
(6) Exclusion of ethnic minority groups (n=4168)	0.95 (0.80,1.13)	0.92 (0.75,1.12)	1.09 (0.81,1.48)
**Substitution of SFA from poultry with milk (2.5 en%)**			
Overall (main result)	1.02 (0.85,1.22)	1.01 (0.82,1.24)	1.12 (0.82,1.54)
(1) Age as underlying timescale	1.02 (0.85,1.21)	1.00 (0.81,1.23)	1.12 (0.82,1.54)
(2) Exclusion of first two years of follow-up (n=1,417)	1.01 (0.83,1.23)	0.97 (0.77,1.21)	1.21 (0.86,1.70)
(3) Exclusion of prevalent diabetes and hypercholesterolemia (n=16,165)	0.98 (0.79,1.21)	0.96 (0.75,1.24)	1.08 (0.74,1.56)
(4) Exclusion of ill/fasting participants (n=3,351)	1.00 (0.83,1.19)	0.98 (0.80,1.22)	1.11 (0.81,1.54)
(5) Restriction to ≥three 24hr-dietary assessments (n=74,934)	1.05 (0.81,1.36)	1.10 (0.81,1.48)	1.11 (0.71,1.73)
(6) Exclusion of ethnic minority groups (n=4168)	1.00 (0.83,1.20)	0.98 (0.79,1.21)	1.11 (0.80,1.53)
**Substitution of SFA from poultry with yogurt (2.5 en%)**			
Overall (main result)	0.88 (0.71,1.09)	0.92 (0.72,1.19)	0.87 (0.59,1.28)
(1) Age as underlying timescale	0.87 (0.70,1.08)	0.91 (0.71,1.18)	0.86 (0.58,1.26)
(2) Exclusion of first two years of follow-up (n=1,417)	0.92 (0.72,1.16)	0.93 (0.71,1.22)	1.01 (0.67,1.52)
(3) Exclusion of prevalent diabetes and hypercholesterolemia (n=16,165)	0.82 (0.63,1.05)	0.85 (0.63,1.15)	0.79 (0.51,1.23)
(4) Exclusion of ill/fasting participants (n=3,351)	0.85 (0.68,1.06)	0.90 (0.70,1.17)	0.85 (0.57,1.26)
(5) Restriction to ≥three 24hr-dietary assessments (n=74,934)	0.88 (0.64,1.19)	0.95 (0.66,1.36)	0.86 (0.50,1.46)

	Hazard ratio (95% confidence intervals)*		
	Cardiovascular diseases	Coronary heart diseases	Stroke
(6) Exclusion of ethnic minority groups (n=4168)	0.84 (0.67,1.05)	0.88 (0.68,1.14)	0.84 (0.57,1.24)
**Substitution of SFA from poultry with cheese (2.5 en%)**			
Overall (main result)	0.97 (0.82,1.15)	0.94 (0.77,1.14)	1.13 (0.84,1.52)
(1) Age as underlying timescale	0.97 (0.82,1.15)	0.93 (0.77,1.13)	1.13 (0.84,1.53)
(2) Exclusion of first two years of follow-up (n=1,417)	0.96 (0.80,1.15)	0.89 (0.72,1.10)	1.24 (0.90,1.71)
(3) Exclusion of prevalent diabetes and hypercholesterolemia (n=16,165)	0.92 (0.76,1.12)	0.90 (0.71,1.13)	1.05 (0.74,1.49)
(4) Exclusion of ill/fasting participants (n=3,351)	0.94 (0.79,1.12)	0.91 (0.74,1.11)	1.13 (0.83,1.53)
(5) Restriction to ≥three 24hr-dietary assessments (n=74,934)	1.02 (0.80,1.31)	1.05 (0.79,1.40)	1.12 (0.73,1.71)
(6) Exclusion of ethnic minority groups (n=4168)	0.95 (0.80,1.13)	0.91 (0.74,1.11)	1.12 (0.82,1.52)
**Substitution of SFA from unprocessed poultry with total dairy (2.5 en%)**			
Overall (main result)	1.05 (0.88, 1.25)	1.02 (0.83,1.26)	1.17 (0.85, 1.60)
(1) Age as underlying timescale	1.04 (0.87,1.25)	1.02 (0.83,1.25)	1.17 (0.85,1.60)
(2) Exclusion of first two years of follow-up (n=1,417)	1.05 (0.87,1.28)	0.99 (0.79,1.24)	1.27 (0.91,1.79)
(3) Exclusion of prevalent diabetes and hypercholesterolemia (n=16,165)	1.00 (0.81,1.23)	0.97 (0.76,1.25)	1.12 (0.77,1.61)
(4) Exclusion of ill/fasting participants (n=3,351)	1.02 (0.85,1.22)	1.00 (0.81,1.23)	1.16 (0.84,1.61)
(5) Restriction to ≥three 24hr-dietary assessments (n=74,934)	1.08 (0.83,1.40)	1.07 (0.79,1.45)	1.21 (0.77,1.90)
(6) Exclusion of ethnic minority groups (n=4168)	1.02 (0.85,1.23)	1.00 (0.81,1.24)	1.15 (0.83,1.59)
**Substitution of SFA from unprocessed poultry with milk (2.5 en%)**			
Overall (main result)	1.10 (0.91,1.32)	1.08 (0.87,1.35)	1.18 (0.84,1.65)
(1) Age as underlying timescale	1.09 (0.90,1.32)	1.08 (0.86,1.34)	1.18 (0.84,1.65)
(2) Exclusion of first two years of follow-up (n=1,417)	1.10 (0.90,1.35)	1.05 (0.83,1.34)	1.26 (0.88,1.81)
(3) Exclusion of prevalent diabetes and hypercholesterolemia (n=16,165)	1.06 (0.84,1.32)	1.04 (0.79,1.35)	1.16 (0.79,1.72)
(4) Exclusion of ill/fasting participants (n=3,351)	1.07 (0.88,1.30)	1.06 (0.85,1.33)	1.17 (0.83,1.65)
(5) Restriction to ≥three 24hr-dietary assessments (n=74,934)	1.12 (0.85,1.47)	1.12 (0.81,1.54)	1.23 (0.76,1.97)
(6) Exclusion of ethnic minority groups (n=4168)	1.07 (0.88,1.30)	1.06 (0.85,1.33)	1.16 (0.82,1.63)
**Substitution of SFA from unprocessed poultry with yogurt (2.5 en%)**			
Overall (main result)	0.94 (0.75,1.18)	0.99 (0.76,1.29)	0.92 (0.61,1.37)
(1) Age as underlying timescale	0.94 (0.74,1.17)	0.98 (0.75,1.28)	0.90 (0.61,1.35)
(2) Exclusion of first two years of follow-up (n=1,417)	0.99 (0.78,1.27)	1.01 (0.76,1.35)	1.05 (0.69,1.61)
(3) Exclusion of prevalent diabetes and hypercholesterolemia (n=16,165)	0.88 (0.68,1.15)	0.92 (0.67,1.25)	0.86 (0.54,1.36)
(4) Exclusion of ill/fasting participants (n=3,351)	0.91 (0.72,1.15)	0.97 (0.74,1.27)	0.89 (0.60,1.34)
(5) Restriction to ≥three 24hr-dietary assessments (n=74,934)	0.93 (0.68,1.29)	0.96 (0.66,1.41)	0.95 (0.55,1.66)
(6) Exclusion of ethnic minority groups (n=4168)	0.91 (0.72,1.14)	0.96 (0.73,1.25)	0.88 (0.58,1.32)
**Substitution of SFA from unprocessed poultry with cheese (2.5 en%)**			
Overall (main result)	1.04 (0.87,1.25)	1.01 (0.82,1.24)	1.19 (0.86,1.64)
(1) Age as underlying timescale	1.04 (0.87,1.25)	1.00 (0.81,1.24)	1.19 (0.87,1.64)
(2) Exclusion of first two years of follow-up (n=1,417)	1.04 (0.86,1.27)	0.97 (0.77,1.22)	1.30 (0.92,1.82)
(3) Exclusion of prevalent diabetes and hypercholesterolemia (n=16,165)	1.00 (0.80,1.23)	0.96 (0.75,1.24)	1.14 (0.79,1.65)
(4) Exclusion of ill/fasting participants (n=3,351)	1.02 (0.85,1.22)	0.98 (0.79,1.22)	1.19 (0.86,1.65)

	Hazard ratio (95% confidence intervals)*		
	Cardiovascular diseases	Coronary heart diseases	Stroke
(5) Restriction to ≥three 24hr-dietary assessments (n=74,934)	1.09 (0.84,1.41)	1.07 (0.79,1.45)	1.25 (0.79,1.96)
(6) Exclusion of ethnic minority groups (n=4168)	1.02 (0.85,1.23)	0.99 (0.80,1.23)	1.17 (0.85,1.63)
**Substitution of SFA from processed poultry with total dairy (2.5 en%)**			
Overall (main result)	0.64 (0.43,0.94)	0.63 (0.40, 0.98)	0.79 (0.38, 1.64)
(1) Age as underlying timescale	0.64 (0.43,0.94)	0.62 (0.40,0.97)	0.80 (0.39,1.66)
(2) Exclusion of first two years of follow-up (n=1,417)	0.60 (0.39,0.91)	0.57 (0.35,0.92)	0.93 (0.42,2.05)
(3) Exclusion of prevalent diabetes and hypercholesterolemia (n=16,165)	0.59 (0.38,0.94)	0.61 (0.36,1.04)	0.63 (0.28,1.45)
(4) Exclusion of ill/fasting participants (n=3,351)	0.62 (0.41,0.92)	0.60 (0.38,0.94)	0.79 (0.37,1.66)
(5) Restriction to ≥three 24hr-dietary assessments (n=74,934)	0.68 (0.37,1.24)	0.92 (0.45,1.90)	0.54 (0.19,1.57)
(6) Exclusion of ethnic minority groups (n=4168)	0.61 (0.41,0.91)	0.58 (0.36,0.91)	0.80 (0.38,1.71)
**Substitution of SFA from processed poultry with milk (2.5 en%)**			
Overall (main result)	0.67 (0.45,0.99)	0.66 (0.42,1.04)	0.81 (0.38,1.69)
(1) Age as underlying timescale	0.66 (0.45,0.99)	0.65 (0.41,1.03)	0.81 (0.39,1.71)
(2) Exclusion of first two years of follow-up (n=1,417)	0.63 (0.41,0.96)	0.60 (0.37,0.98)	0.93 (0.42,2.07)
(3) Exclusion of prevalent diabetes and hypercholesterolemia (n=16,165)	0.63 (0.40,1.00)	0.65 (0.38,1.11)	0.65 (0.28,1.52)
(4) Exclusion of ill/fasting participants (n=3,351)	0.65 (0.43,0.97)	0.63 (0.40,1.00)	0.80 (0.37,1.70)
(5) Restriction to ≥three 24hr-dietary assessments (n=74,934)	0.70 (0.38,1.30)	0.97 (0.47,2.02)	0.55 (0.19,1.61)
(6) Exclusion of ethnic minority groups (n=4168)	0.64 (0.42,0.96)	0.61 (0.38,0.97)	0.81 (0.38,1.75)
**Substitution of SFA from processed poultry with yogurt (2.5 en%)**			
Overall (main result)	0.58 (0.38,0.87)	0.61 (0.38,0.98)	0.63 (0.29,1.35)
(1) Age as underlying timescale	0.57 (0.38,0.86)	0.60 (0.37,0.96)	0.62 (0.29,1.35)
(2) Exclusion of first two years of follow-up (n=1,417)	0.56 (0.36,0.88)	0.58 (0.34,0.97)	0.77 (0.34,1.78)
(3) Exclusion of prevalent diabetes and hypercholesterolemia (n=16,165)	0.53 (0.32,0.85)	0.57 (0.33,1.01)	0.48 (0.20,1.16)
(4) Exclusion of ill/fasting participants (n=3,351)	0.55 (0.36,0.84)	0.58 (0.36,0.94)	0.61 (0.28,1.34)
(5) Restriction to ≥three 24hr-dietary assessments (n=74,934)	0.59 (0.31,1.11)	0.84 (0.39,1.79)	0.43 (0.14,1.30)
(6) Exclusion of ethnic minority groups (n=4168)	0.54 (0.35,0.82)	0.55 (0.33,0.89)	0.61 (0.28,1.37)
**Substitution of SFA from processed poultry with cheese (2.5 en%)**			
Overall (main result)	0.64 (0.43,0.94)	0.62 (0.39,0.97)	0.81 (0.39,1.69)
(1) Age as underlying timescale	0.64 (0.43,0.94)	0.61 (0.39,0.95)	0.82 (0.40,1.71)
(2) Exclusion of first two years of follow-up (n=1,417)	0.59 (0.39,0.90)	0.55 (0.34,0.90)	0.95 (0.43,2.10)
(3) Exclusion of prevalent diabetes and hypercholesterolemia (n=16,165)	0.59 (0.37,0.94)	0.60 (0.35,1.03)	0.64 (0.28,1.47)
(4) Exclusion of ill/fasting participants (n=3,351)	0.61 (0.41,0.91)	0.58 (0.37,0.92)	0.81 (0.38,1.71)
(5) Restriction to ≥three 24hr-dietary assessments (n=74,934)	0.69 (0.37,1.26)	0.93 (0.45,1.92)	0.56 (0.19,1.62)
(6) Exclusion of ethnic minority groups (n=4168)	0.61 (0.41,0.91)	0.57 (0.36,0.90)	0.82 (0.39,1.75)

**Hazard ratios (95% confidence intervals) were derived from Cox proportional hazard regression models, adjusted for sex (female/males), age (y), ethnic background (white Europeans/south Asians/African Caribbean/multiple ethnic background or other/unknown), education (college or university degree or vocation/national examination at 17-18 years of age/national examination at 16 years of age/unknown), Townsend deprivation index (quintiles), country of assessment centre (England/Scotland/Wales), smoking (current/former/never/unknown), physical activity (low/moderate/high/unknown), alcohol intake (non- drinkers/<1 g/d/1-10 g/d/10-20 g/d/≥20 g/d/unknown), dietary supplement use (yes/no), hormone replacement therapy use (females only)*

(yes/no/unknown), menopausal status (females only) (yes/no/not sure – had a hysterectomy/not sure -other reason/unknown), fruit and vegetables (g/d), fibre (g/d), family history of CVD (yes/no), BMI (underweight/healthy weight/overweight/obesity/unknown), abdominal obesity (yes/no/unknown), baseline hypertension (yes/no), baseline hypercholesterolemia (yes/no) and baseline diabetes (yes/no), trans unsaturated fatty acids (en%), dietary cholesterol (mg/d), protein (en%), carbohydrate (en%), monounsaturated fatty acids (en%), polyunsaturated fatty acids (en%), saturated fatty acids from non-meat and non-dairy sources (en%). Saturated fatty acids from meat and dairy sources were mutually adjusted for in the analyses, as appropriate. Abbreviations. SFA=saturated fatty acids.

Table S22. Hazard ratios (95% confidence intervals) for risk of cardiovascular diseases, coronary heart diseases and cerebrovascular diseases associated with substitution of saturated fatty acids from one type of meat with another among UK Biobank participants (n=120,496): Sensitivity analyses

	Hazard ratio (95% confidence intervals)*		
	**Cardiovascular diseases**	**Coronary heart diseases**	**Stroke**
**Substitution of SFA from processed with unprocessed meat (2.5 en%)**			
Overall (main result)	0.87 (0.76, 0.98)	0.82 (0.71, 0.95)	0.92 (0.74, 1.16)
(1) Age as underlying timescale	0.85 (0.75,0.97)	0.81 (0.70,0.94)	0.90 (0.72,1.13)
(2) Exclusion of first two years of follow-up (n=1,417)	0.89 (0.78,1.02)	0.85 (0.72,1.00)	0.93 (0.74,1.19)
(3) Exclusion of prevalent diabetes and hypercholesterolemia (n=16,165)	0.85 (0.73,0.99)	0.79 (0.66,0.94)	0.96 (0.74,1.25)
(4) Exclusion of ill/fasting participants (n=3,351)	0.86 (0.76,0.98)	0.82 (0.71,0.96)	0.90 (0.72,1.13)
(5) Restriction to ≥three 24hr-dietary assessments (n=74,934)	0.93 (0.79,1.09)	0.91 (0.75,1.10)	0.95 (0.71,1.27)
(6) Exclusion of ethnic minority groups (n=4168)	0.89 (0.78,1.01)	0.85 (0.73,0.98)	0.94 (0.75,1.18)
**Substitution of SFA from processed with unprocessed red meat (2.5 en%)**			
Overall (main result)	0.97 (0.88, 1.07)	0.94 (0.84, 1.05)	1.02 (0.86, 1.21)
(1) Age as underlying timescale	0.97 (0.88,1.08)	0.94 (0.84,1.06)	1.02 (0.85,1.21)
(2) Exclusion of first two years of follow-up (n=1,417)	0.98 (0.89,1.10)	0.94 (0.83,1.06)	1.05 (0.87,1.26)
(3) Exclusion of prevalent diabetes and hypercholesterolemia (n=16,165)	0.96 (0.85,1.08)	0.92 (0.80,1.06)	1.01 (0.82,1.24)
(4) Exclusion of ill/fasting participants (n=3,351)	0.97 (0.88,1.07)	0.94 (0.84,1.06)	0.99 (0.83,1.18)
(5) Restriction to ≥three 24hr-dietary assessments (n=74,934)	0.99 (0.86,1.14)	0.99 (0.84,1.16)	0.98 (0.77,1.25)
(6) Exclusion of ethnic minority groups (n=4168)	0.99 (0.90,1.10)	0.96 (0.86,1.08)	1.04 (0.87,1.24)
**Substitution of SFA from processed with unprocessed poultry (2.5 en%)**			
Overall (main result)	0.61 (0.40, 0.93)	0.61 (0.38, 0.99)	0.68 (0.31, 1.48)
(1) Age as underlying timescale	0.61 (0.40,0.92)	0.61 (0.38,0.98)	0.69 (0.31,1.49)
(2) Exclusion of first two years of follow-up (n=1,417)	0.57 (0.36,0.89)	0.57 (0.34,0.96)	0.73 (0.32,1.69)
(3) Exclusion of prevalent diabetes and hypercholesterolemia (n=16,165)	0.60 (0.36,0.97)	0.63 (0.35,1.11)	0.57 (0.23,1.38)
(4) Exclusion of ill/fasting participants (n=3,351)	0.60 (0.40,0.93)	0.60 (0.37,0.97)	0.68 (0.31,1.50)
(5) Restriction to ≥three 24hr-dietary assessments (n=74,934)	0.62 (0.33,1.19)	0.86 (0.40,1.86)	0.45 (0.14,1.39)
(6) Exclusion of ethnic minority groups (n=4168)	0.59 (0.39,0.92)	0.58 (0.35,0.94)	0.70 (0.31,1.56)
**Substitution of SFA from red meat with poultry (2.5 en%)**			
Overall (main result)	0.97 (0.82, 1.15)	1.03 (0.85, 1.24)	0.86 (0.64, 1.15)
(1) Age as underlying timescale	0.98 (0.83,1.16)	1.04 (0.86,1.26)	0.87 (0.65,1.17)
(2) Exclusion of first two years of follow-up (n=1,417)	0.95 (0.80,1.14)	1.01 (0.82,1.25)	0.81 (0.59,1.11)
(3) Exclusion of prevalent diabetes and hypercholesterolemia (n=16,165)	1.02 (0.84,1.24)	1.07 (0.85,1.35)	0.88 (0.62,1.24)
(4) Exclusion of ill/fasting participants (n=3,351)	1.00 (0.84,1.18)	1.05 (0.86,1.28)	0.85 (0.63,1.15)
(5) Restriction to ≥three 24hr-dietary assessments (n=74,934)	0.87 (0.69,1.11)	0.85 (0.65,1.12)	0.84 (0.56,1.27)
(6) Exclusion of ethnic minority groups (n=4168)	0.99 (0.83,1.17)	1.05 (0.86,1.27)	0.86 (0.64,1.16)

**Hazard ratios (95% confidence intervals) were derived from Cox proportional hazard regression models, adjusted for sex (female/males), age (y), ethnic background (white Europeans/south Asians/African Caribbean/multiple ethnic background or other/unknown), education (college or university degree or vocation/national examination at 17-18 years of age/national examination at 16 years of age/unknown), Townsend deprivation index (quintiles), country of assessment centre (England/Scotland/Wales), smoking (current/former/never/unknown), physical activity*

(low/moderate/high/unknown), alcohol intake (non- drinkers/<1 g/d/1-10 g/d/10-20 g/d/≥20 g/d/unknown), dietary supplement use (yes/no), hormone replacement therapy use (females only) (yes/no/unknown), menopausal status (females only) (yes/no/not sure – had a hysterectomy/not sure -other reason/unknown), fruit and vegetables (g/d), fibre (g/d), family history of CVD (yes/no), BMI (underweight/healthy weight/overweight/obesity/unknown), abdominal obesity (yes/no/unknown), baseline hypertension (yes/no), baseline hypercholesterolemia (yes/no) and baseline diabetes (yes/no), trans unsaturated fatty acids (en%), dietary cholesterol (mg/d), protein (en%), carbohydrate (en%), monounsaturated fatty acids (en%), polyunsaturated fatty acids (en%), saturated fatty acids from non-meat sources (en%). Saturated fatty acids from meat sources were mutually adjusted for in the analyses, as appropriate. Abbreviations. SFA=saturated fatty acids.

Table S23. Hazard ratios (95% confidence intervals) for risk of cardiovascular diseases associated with substitution of saturated fatty acids from total and subtypes of meats with total and subtypes of dairy in the UK Biobank Study (n=120,496): Stratified analyses

Effect modifier	Strata	N cases/total	Hazard ratio (95% confidence intervals) for CVD risk associated with the substitution of SFA (2.5 en%) from*
			**Total meat with:**
			Total dairy
Sex	Females	3,879/68,689	0.91 (0.83,1.00)
	Males	6,011/51,807	0.91 (0.85,0.97)
			P-interaction= 0.08
Age	<57 years	2,455/58,268	0.87 (0.78,0.97)
	≥57 years	7,435/62,228	0.92 (0.86,0.98)
			P-interaction= 0.10
BMI	<25 kg/m^2	2,932/48,578	0.93 (0.84,1.04)
	≥25 kg/m^2	6,924/71,663	0.90 (0.84,0.96)
			P-interaction= 0.71
Abdominal obesity	No	6,234/80,518	0.94 (0.88,1.00)
	Yes	3,639/29,952	0.86 (0.78,0.93)
			P-interaction= 0.60
Baseline hypertension	No	5,723/85,710	0.89 (0.83,0.96)
	Yes	4,167/24,896	0.93 (0.85,1.01)
			P-interaction= 0.35
Baseline hypercholesterolemia	No	7,252/97,661	0.89 (0.84,0.95)
	Yes	2,638/12,945	0.94 (0.85,1.05)
			P-interaction= 0.40
Baseline diabetes	No	9,302/108,434	0.90 (0.85,0.95)
	Yes	588/2,172	1.06 (0.86,1.31)
			P-interaction= 0.44

Effect modifier	Strata	N cases/total	Hazard ratio (95% confidence intervals) for CVD risk associated with the substitution of SFA (2.5 en%) from*			
			Unprocessed meat with:			
			**Total dairy**	**Milk**	**Yogurt**	**Cheese**
Sex	Females	3,879/68,689	1.00 (0.84,1.19)	1.12 (0.91,1.38)	0.84 (0.64,1.11)	1.00 (0.83,1.19)
	Males	6,011/51,807	1.02 (0.89,1.17)	1.03 (0.87,1.21)	0.97 (0.76,1.24)	1.02 (0.89,1.18)
			P-interaction= 0.07	P-interaction= 0.010	P-interaction= 0.54	P-interaction= 0.39
Age	<57 years	2,455/58,268	0.98 (0.79,1.22)	1.09 (0.85,1.41)	0.83 (0.57,1.20)	0.98 (0.79,1.22)
	≥57 years	7,435/62,228	1.02 (0.90,1.16)	1.06 (0.91,1.23)	0.96 (0.78,1.18)	1.03 (0.91,1.17)
			P-interaction= 0.27	P-interaction= 0.07	P-interaction= 0.22	P-interaction= 0.28
BMI	<25 kg/m^2	2,932/48,578	1.08 (0.88,1.33)	1.15 (0.90,1.47)	1.19 (0.87,1.63)	1.07 (0.86,1.32)
	≥25 kg/m^2	6,924/71,663	0.99 (0.87,1.12)	1.04 (0.89,1.20)	0.81 (0.65,1.02)	0.99 (0.87,1.13)
			P-interaction= 0.64	P-interaction= 0.80	P-interaction= 0.76	P-interaction= 0.28
Abdominal obesity	No	6,234/80,518	1.07 (0.93,1.23)	1.12 (0.95,1.33)	1.08 (0.86,1.35)	1.07 (0.93,1.23)
	Yes	3,639/29,952	0.92 (0.77,1.09)	0.98 (0.80,1.20)	0.69 (0.50,0.94)	0.93 (0.78,1.10)
			P-interaction= 0.75	P-interaction= 0.84	P-interaction= 0.43	P-interaction= 0.89
Baseline hypertension	No	5,723/85,710	0.99 (0.86,1.15)	1.01 (0.85,1.20)	0.82 (0.65,1.04)	1.01 (0.87,1.17)
	Yes	4,167/24,896	1.05 (0.89,1.23)	1.17 (0.96,1.42)	1.13 (0.85,1.51)	1.03 (0.87,1.21)
			P-interaction= 0.34	P-interaction= 0.35	P-interaction= 0.043	P-interaction= 0.85
Baseline hypercholesterolemia	No	7,252/97,661	1.00 (0.88,1.13)	1.07 (0.92,1.24)	0.90 (0.73,1.11)	1.00 (0.88,1.14)
	Yes	2,638/12,945	1.05 (0.86,1.29)	1.06 (0.83,1.36)	1.04 (0.72,1.51)	1.05 (0.86,1.30)
			P-interaction= 0.51	P-interaction= 0.78	P-interaction= 0.46	P-interaction= 0.42
Baseline diabetes	No	9,302/108,434	1.01 (0.90,1.13)	1.07 (0.94,1.22)	0.93 (0.78,1.13)	1.01 (0.91,1.14)
	Yes	588/2,172	1.10 (0.71,1.68)	1.21 (0.72,2.01)	0.85 (0.36,2.00)	1.10 (0.72,1.69)
			P-interaction= 0.26	P-interaction= 0.35	P-interaction= 0.63	P-interaction= 0.08

Effect modifier	Strata	N cases/total	Hazard ratio (95% confidence intervals) for CVD risk associated with the substitution of SFA (2.5 en%) from*			
			**Processed meat with:**			
			**Total dairy**	**Milk**	**Yogurt**	**Cheese**
Sex	Females	3,879/68,689	0.86 (0.75,0.98)	0.96 (0.81,1.14)	0.72 (0.56,0.92)	0.85 (0.74,0.98)
	Males	6,011/51,807	0.89 (0.81,0.97)	0.87 (0.77,0.99)	0.83 (0.66,1.03)	0.87 (0.79,0.96)
			P-interaction= 0.09	P-interaction= 0.015	P-interaction= 0.81	P-interaction= 0.52
Age	<57 years	2,455/58,268	0.84 (0.72,0.97)	0.91 (0.75,1.11)	0.69 (0.49,0.96)	0.82 (0.70,0.96)
	≥57 years	7,435/62,228	0.89 (0.81,0.97)	0.91 (0.81,1.02)	0.82 (0.68,0.99)	0.88 (0.80,0.97)
			P-interaction= 0.145	P-interaction= 0.038	P-interaction= 0.123	P-interaction= 0.147
BMI	<25 kg/m^2	2,932/48,578	0.89 (0.76,1.05)	0.91 (0.75,1.11)	0.94 (0.71,1.25)	0.84 (0.71,1.00)
	≥25 kg/m^2	6,924/71,663	0.87 (0.80,0.95)	0.91 (0.81,1.03)	0.72 (0.59,0.88)	0.88 (0.80,0.96)
			P-interaction= 0.69	P-interaction= 0.81	P-interaction= 0.70	P-interaction= 0.30
Abdominal obesity	No	6,234/80,518	0.90 (0.81,0.99)	0.92 (0.81,1.05)	0.89 (0.73,1.08)	0.88 (0.79,0.98)
	Yes	3,639/29,952	0.85 (0.75,0.95)	0.90 (0.76,1.05)	0.63 (0.47,0.84)	0.85 (0.75,0.96)
			P-interaction= 0.59	P-interaction= 0.66	P-interaction= 0.36	P-interaction= 0.70
Baseline hypertension	No	5,723/85,710	0.86 (0.78,0.95)	0.87 (0.76,0.99)	0.71 (0.57,0.87)	0.86 (0.78,0.96)
	Yes	4,167/24,896	0.90 (0.80,1.01)	0.98 (0.84,1.14)	0.95 (0.73,1.23)	0.86 (0.76,0.97)
			P-interaction= 0.34	P-interaction= 0.34	P-interaction= 0.45	P-interaction= 0.83
Baseline hypercholesterolemia	No	7,252/97,661	0.86 (0.78,0.94)	0.90 (0.80,1.01)	0.76 (0.63,0.91)	0.84 (0.76,0.93)
	Yes	2,638/12,945	0.93 (0.81,1.07)	0.93 (0.76,1.13)	0.92 (0.65,1.29)	0.92 (0.79,1.08)
			P-interaction= 0.32	P-interaction= 0.34	P-interaction= 0.29	P-interaction= 0.25
Baseline diabetes	No	9,302/108,434	0.87 (0.80,0.94)	0.90 (0.81,0.99)	0.78 (0.66,0.93)	0.85 (0.78,0.93)
	Yes	588/2,172	0.97 (0.73,1.30)	1.14 (0.77,1.68)	0.80 (0.36,1.78)	1.04 (0.77,1.41)
			P-interaction= 0.42	P-interaction= 0.52	P-interaction= 0.93	P-interaction= 0.14

Effect modifier	Strata	N cases/total	Hazard ratio (95% confidence intervals) for CVD risk associated with the substitution of SFA (2.5 en%) from*			
			**Red meat with:**			
			**Total dairy**	**Milk**	**Yogurt**	**Cheese**
Sex	Females	3,879/68,689	0.93 (0.81,1.08)	1.05 (0.88,1.25)	0.78 (0.61,1.01)	0.93 (0.80,1.08)
	Males	6,011/51,807	0.95 (0.86,1.05)	0.95 (0.83,1.09)	0.90 (0.72,1.13)	0.95 (0.85,1.06)
			P-interaction= 0.10	P-interaction= 0.015	P-interaction= 0.89	P-interaction= 0.59
Age	<57 years	2,455/58,268	0.89 (0.76,1.05)	0.98 (0.80,1.21)	0.74 (0.52,1.05)	0.89 (0.75,1.05)
	≥57 years	7,435/62,228	0.97 (0.88,1.07)	0.99 (0.88,1.13)	0.89 (0.74,1.08)	0.97 (0.87,1.07)
			P-interaction= 0.08	P-interaction= 0.030	P-interaction= 0.06	P-interaction= 0.08
BMI	<25 kg/m^2	2,932/48,578	1.00 (0.84,1.18)	1.05 (0.85,1.29)	1.08 (0.81,1.44)	0.98 (0.82,1.16)
	≥25 kg/m^2	6,924/71,663	0.93 (0.84,1.02)	0.97 (0.86,1.11)	0.76 (0.62,0.94)	0.94 (0.85,1.04)
			P-interaction= 0.70	P-interaction= 0.84	P-interaction= 0.76	P-interaction= 0.31
Abdominal obesity	No	6,234/80,518	1.01 (0.91,1.13)	1.05 (0.92,1.21)	1.01 (0.82,1.24)	1.01 (0.90,1.13)
	Yes	3,639/29,952	0.86 (0.75,0.98)	0.91 (0.77,1.08)	0.64 (0.48,0.86)	0.86 (0.76,0.99)
			P-interaction= 0.65	P-interaction= 0.71	P-interaction= 0.39	P-interaction= 0.76
Baseline hypertension	No	5,723/85,710	0.93 (0.83,1.04)	0.94 (0.81,1.08)	0.77 (0.62,0.95)	0.94 (0.84,1.05)
	Yes	4,167/24,896	0.97 (0.86,1.10)	1.08 (0.91,1.27)	1.04 (0.79,1.35)	0.95 (0.83,1.08)
			P-interaction= 0.36	P-interaction= 0.37	P-interaction= 0.047	P-interaction= 0.89
Baseline hypercholesterolemia	No	7,252/97,661	0.94 (0.85,1.04)	1.00 (0.88,1.13)	0.84 (0.69,1.01)	0.93 (0.84,1.04)
	Yes	2,638/12,945	0.97 (0.83,1.13)	0.97 (0.79,1.19)	0.95 (0.67,1.34)	0.97 (0.82,1.13)
			P-interaction= 0.47	P-interaction= 0.71	P-interaction= 0.44	P-interaction= 0.39
Baseline diabetes	No	9,302/108,434	0.94 (0.86,1.03)	0.98 (0.88,1.10)	0.86 (0.72,1.02)	0.94 (0.86,1.02)
	Yes	588/2,172	1.08 (0.78,1.49)	1.23 (0.80,1.87)	0.85 (0.38,1.90)	1.11 (0.80,1.54)
			P-interaction= 0.37	P-interaction= 0.49	P-interaction= 0.85	P-interaction= 0.12

Effect modifier	Strata	N cases/total	Hazard ratio (95% confidence intervals) for CVD risk associated with the substitution of SFA (2.5 en%) from*			
			Unprocessed red meat with:			
			**Total dairy**	**Milk**	**Yogurt**	**Cheese**
Sex	Females	3,879/68,689	0.93 (0.83,1.05)	1.04 (0.89,1.22)	0.78 (0.61,0.99)	0.93 (0.83,1.05)
	Males	6,011/51,807	0.90 (0.82,0.99)	0.90 (0.79,1.02)	0.86 (0.69,1.07)	0.91 (0.83,0.99)
			P-interaction= 0.08	P-interaction= 0.010	P-interaction= 0.64	P-interaction= 0.45
Age	<57 years	2,455/58,268	0.86 (0.75,0.99)	0.94 (0.78,1.14)	0.72 (0.51,1.00)	0.86 (0.75,0.99)
	≥57 years	7,435/62,228	0.93 (0.86,1.01)	0.96 (0.85,1.07)	0.87 (0.72,1.04)	0.94 (0.86,1.02)
			P-interaction= 0.17	P-interaction= 0.048	P-interaction= 0.14	P-interaction= 0.17
BMI	<25 kg/m^2	2,932/48,578	0.93 (0.81,1.06)	0.98 (0.81,1.17)	1.01 (0.77,1.32)	0.91 (0.79,1.05)
	≥25 kg/m^2	6,924/71,663	0.90 (0.83,0.98)	0.94 (0.84,1.06)	0.74 (0.61,0.91)	0.91 (0.84,0.99)
			P-interaction= 0.54	P-interaction= 0.65	P-interaction= 0.68	P-interaction= 0.24
Abdominal obesity	No	6,234/80,518	0.93 (0.85,1.02)	0.97 (0.86,1.10)	0.93 (0.77,1.14)	0.93 (0.85,1.02)
	Yes	3,639/29,952	0.87 (0.77,0.97)	0.92 (0.78,1.08)	0.65 (0.49,0.87)	0.88 (0.78,0.99)
			P-interaction= 0.60	P-interaction= 0.64	P-interaction= 0.34	P-interaction= 0.71
Baseline hypertension	No	5,723/85,710	0.89 (0.81,0.98)	0.90 (0.79,1.03)	0.74 (0.60,0.91)	0.91 (0.83,1.00)
	Yes	4,167/24,896	0.93 (0.84,1.04)	1.03 (0.89,1.20)	1.00 (0.77,1.30)	0.92 (0.82,1.02)
			P-interaction= 0.34	P-interaction= 0.33	P-interaction= 0.042	P-interaction= 0.85
Baseline hypercholesterolemia	No	7,252/97,661	0.90 (0.82,0.98)	0.95 (0.85,1.07)	0.80 (0.67,0.97)	0.90 (0.83,0.98)
	Yes	2,638/12,945	0.94 (0.82,1.08)	0.95 (0.78,1.15)	0.93 (0.67,1.31)	0.95 (0.82,1.09)
			P-interaction= 0.49	P-interaction= 0.77	P-interaction= 0.43	P-interaction= 0.41
Baseline diabetes	No	9,302/108,434	0.90 (0.84,0.97)	0.94 (0.85,1.04)	0.83 (0.70,0.98)	0.91 (0.84,0.98)
	Yes	588/2,172	1.07 (0.81,1.40)	1.17 (0.80,1.71)	0.82 (0.37,1.83)	1.07 (0.81,1.41)
			P-interaction= 0.35	P-interaction= 0.44	P-interaction= 0.80	P-interaction= 0.11

Effect modifier	Strata	N cases/total	Hazard ratio (95% confidence intervals) for CVD risk associated with the substitution of SFA (2.5 en%) from*			
			Processed red meat with:			
			**Total dairy**	**Milk**	**Yogurt**	**Cheese**
Sex	Females	3,879/68,689	0.87 (0.76,0.99)	0.98 (0.83,1.16)	0.73 (0.57,0.94)	0.87 (0.76,1.00)
	Males	6,011/51,807	0.89 (0.81,0.98)	0.88 (0.77,1.00)	0.84 (0.67,1.05)	0.89 (0.80,0.98)
			P-interaction= 0.08	P-interaction= 0.012	P-interaction= 0.78	P-interaction= 0.49
Age	<57 years	2,455/58,268	0.85 (0.73,0.98)	0.92 (0.76,1.12)	0.70 (0.50,0.98)	0.84 (0.72,0.99)
	≥57 years	7,435/62,228	0.90 (0.82,0.98)	0.91 (0.81,1.03)	0.83 (0.69,1.00)	0.89 (0.81,0.99)
			P-interaction= 0.20	P-interaction= 0.05	P-interaction= 0.17	P-interaction= 0.21
BMI	<25 kg/m^2	2,932/48,578	0.91 (0.78,1.08)	0.94 (0.77,1.15)	0.97 (0.73,1.29)	0.87(0.74,1.04)
	≥25 kg/m^2	6,924/71,663	0.88 (0.80,0.96)	0.92 (0.81,1.03)	0.72 (0.59,0.89)	0.89 (0.81,0.98)
			P-interaction= 0.68	P-interaction= 0.77	P-interaction= 0.67	P-interaction= 0.30
Abdominal obesity	No	6,234/80,518	0.92 (0.83,1.02)	0.95 (0.83,1.08)	0.91 (0.75,1.12)	0.91 (0.81,1.01)
	Yes	3,639/29,952	0.84 (0.75,0.95)	0.89 (0.76,1.05)	0.63 (0.47,0.84)	0.85 (0.75,0.96)
			P-interaction= 0.42	P-interaction= 0.45	P-interaction= 0.27	P-interaction= 0.50
Baseline hypertension	No	5,723/85,710	0.87 (0.78,0.96)	0.87 (0.76,1.00)	0.72 (0.58,0.89)	0.88 (0.79,0.98)
	Yes	4,167/24,896	0.91 (0.81,1.02)	0.99 (0.85,1.16)	0.96 (0.74,1.25)	0.88 (0.78,1.00)
			P-interaction= 0.35	P-interaction= 0.34	P-interaction= 0.046	P-interaction= 0.87
Baseline hypercholesterolemia	No	7,252/97,661	0.86 (0.79,0.95)	0.91 (0.81,1.02)	0.77 (0.64,0.93)	0.86 (0.78,0.95)
	Yes	2,638/12,945	0.94 (0.82,1.09)	0.94 (0.77,1.15)	0.93 (0.66,1.31)	0.94 (0.81,1.10)
			P-interaction= 0.38	P-interaction= 0.57	P-interaction= 0.34	P-interaction= 0.30
Baseline diabetes	No	9,302/108,434	0.88 (0.81,0.95)	0.90 (0.81,1.00)	0.79 (0.67,0.94)	0.87 (0.80,0.95)
	Yes	588/2,172	1.01 (0.75,1.35)	1.18 (0.79,1.74)	0.82 (0.37,1.83)	1.07 (0.79,1.46)
			P-interaction= 0.41	P-interaction= 0.50	P-interaction= 0.90	P-interaction= 0.14

Effect modifier	Strata	N cases/total	Hazard ratio (95% confidence intervals) for CVD risk associated with the substitution of SFA (2.5 en%) from*			
			Poultry with:			
			Total dairy	Milk	Yogurt	Cheese
Sex	Females	3,879/68,689	1.11 (0.86,1.45)	1.24 (0.94,1.65)	0.93 (0.67,1.29)	1.10 (0.85,1.44)
	Males	6,011/51,807	0.89 (0.72,1.10)	0.89 (0.70,1.12)	0.84 (0.63,1.13)	0.89 (0.72,1.11)
			P-interaction= 0.036	P-interaction= 0.005	P-interaction= 0.24	P-interaction= 0.18
Age	<57 years	2,455/58,268	0.92 (0.67,1.25)	1.01 (0.72,1.41)	0.76 (0.50,1.17)	0.91 (0.66,1.25)
	≥57 years	7,435/62,228	1.00 (0.82,1.22)	1.03 (0.83,1.27)	0.92 (0.72,1.19)	1.00 (0.82,1.22)
			P-interaction= 0.54	P-interaction= 0.11	P-interaction= 0.53	P-interaction= 0.65
BMI	<25 kg/m^2	2,932/48,578	1.04 (0.75,1.45)	1.10 (0.77,1.55)	1.13 (0.76,1.68)	1.02 (0.73,1.42)
	≥25 kg/m^2	6,924/71,663	0.96 (0.79,1.17)	1.01 (0.82,1.24)	0.79 (0.61,1.03)	0.97 (0.80,1.18)
			P-interaction= 0.69	P-interaction= 0.80	P-interaction= 0.66	P-interaction= 0.30
Abdominal obesity	No	6,234/80,518	0.99 (0.80,1.23)	1.03 (0.81,1.29)	0.98 (0.75,1.29)	0.98 (0.79,1.22)
	Yes	3,639/29,952	0.97 (0.74,1.26)	1.03 (0.78,1.37)	0.72 (0.50,1.04)	0.98 (0.75,1.27)
			P-interaction= 0.69	P-interaction= 0.76	P-interaction= 0.40	P-interaction= 0.81
Baseline hypertension	No	5,723/85,710	0.88 (0.71,1.10)	0.89 (0.71,1.13)	0.73 (0.55,0.97)	0.89 (0.72,1.12)
	Yes	4,167/24,896	1.12 (0.87,1.44)	1.23 (0.93,1.61)	1.18 (0.84,1.66)	1.08 (0.84,1.40)
			P-interaction= 0.36	P-interaction= 0.37	P-interaction= 0.048	P-interaction= 0.89
Baseline hypercholesterolemia	No	7,252/97,661	0.92 (0.76,1.12)	0.98 (0.79,1.21)	0.82 (0.64,1.06)	0.92 (0.75,1.12)
	Yes	2,638/12,945	1.15 (0.83,1.58)	1.15 (0.81,1.62)	1.12 (0.72,1.75)	1.14 (0.83,1.58)
			P-interaction= 0.56	P-interaction= 0.88	P-interaction= 0.52	P-interaction= 0.47
Baseline diabetes	No	9,302/108,434	0.95 (0.80,1.13)	1.00 (0.83,1.20)	0.87 (0.70,1.09)	0.95 (0.80,1.13)
	Yes	588/2,172	1.34 (0.69,2.60)	1.46 (0.72,2.99)	1.01 (0.37,2.76)	1.33 (0.68,2.58)
			P-interaction= 0.44	P-interaction= 0.54	P-interaction= 0.95	P-interaction= 0.15

Effect modifier	Strata	N cases/total	Hazard ratio (95% confidence intervals) for CVD risk associated with the substitution of SFA (2.5 en%) from*			
			**Unprocessed poultry with:**			
			**Total dairy**	**Milk**	**Yogurt**	**Cheese**
Sex	Females	3,879/68,689	1.19 (0.90,1.57)	1.32 (0.99,1.78)	0.99 (0.70,1.39)	1.17 (0.89,1.56)
	Males	6,011/51,807	0.96 (0.76,1.22)	0.96 (0.75,1.24)	0.91 (0.67,1.24)	0.96 (0.76,1.22)
			P-interaction= 0.045	P-interaction= 0.006	P-interaction= 0.32	P-interaction= 0.23
Age	<57 years	2,455/58,268	1.00 (0.71,1.41)	1.10 (0.77,1.59)	0.83 (0.53,1.30)	1.00 (0.71,1.40)
	≥57 years	7,435/62,228	1.06 (0.86,1.31)	1.09 (0.87,1.37)	0.98 (0.75,1.28)	1.06 (0.86,1.31)
			P-interaction= 0.69	P-interaction= 0.13	P-interaction= 0.75	P-interaction= 0.88
BMI	<25 kg/m^2	2,932/48,578	1.16 (0.82,1.65)	1.23 (0.85,1.77)	1.26 (0.83,1.91)	1.14 (0.80,1.62)
	≥25 kg/m^2	6,924/71,663	1.02 (0.82,1.25)	1.06 (0.85,1.33)	0.83 (0.63,1.10)	1.02 (0.83,1.26)
			P-interaction= 0.65	P-interaction= 0.75	P-interaction= 0.60	P-interaction= 0.28
Abdominal obesity	No	6,234/80,518	1.13 (0.90,1.43)	1.17 (0.91,1.50)	1.12 (0.84,1.49)	1.12 (0.88,1.41)
	Yes	3,639/29,952	0.95 (0.72,1.26)	1.02 (0.75,1.38)	0.71 (0.49,1.04)	0.96 (0.72,1.28)
			P-interaction= 0.35	P-interaction= 0.40	P-interaction= 0.23	P-interaction= 0.42
Baseline hypertension	No	5,723/85,710	0.95 (0.75,1.20)	0.96 (0.75,1.24)	0.79 (0.58,1.06)	0.96 (0.76,1.22)
	Yes	4,167/24,896	1.20 (0.91,1.58)	1.31 (0.98,1.76)	1.26 (0.89,1.80)	1.16 (0.88,1.53)
			P-interaction= 0.35	P-interaction= 0.36	P-interaction= 0.049	P-interaction= 0.89
Baseline hypercholesterolemia	No	7,252/97,661	1.00 (0.81,1.23)	1.06 (0.85,1.32)	0.89 (0.68,1.16)	0.99 (0.80,1.23)
	Yes	2,638/12,945	1.22 (0.86,1.72)	1.22 (0.84,1.76)	1.19 (0.76,1.88)	1.21 (0.86,1.72)
			P-interaction= 0.54	P-interaction= 0.86	P-interaction= 0.51	P-interaction= 0.44
Baseline diabetes	No	9,302/108,434	1.02 (0.85,1.23)	1.07 (0.88,1.30)	0.93 (0.74,1.18)	1.02 (0.84,1.22)
	Yes	588/2,172	1.60 (0.77,3.31)	1.74 (0.81,3.77)	1.18 (0.42,3.31)	1.59 (0.77,3.30)
			P-interaction= 0.41	P-interaction= 0.51	P-interaction= 0.86	P-interaction= 0.13

Effect modifier	Strata	N cases/total	Hazard ratio (95% confidence intervals) for CVD risk associated with the substitution of SFA (2.5 en%) from*			
			Processed poultry with:			
			Total dairy	Milk	Yogurt	Cheese
Sex	Females	3,879/68,689	0.71 (0.36,1.39)	0.80 (0.41,1.58)	0.60 (0.30,1.21)	0.71 (0.36,1.39)
	Males	6,011/51,807	0.60 (0.37,0.96)	0.59 (0.36,0.96)	0.56 (0.33,0.94)	0.59 (0.37,0.96)
			P-interaction= 0.10	P-interaction= 0.015	P-interaction= 0.90	P-interaction= 0.59
Age	<57 years	2,455/58,268	0.62 (0.32,1.17)	0.68 (0.35,1.32)	0.52 (0.25,1.05)	0.62 (0.32,1.18)
	≥57 years	7,435/62,228	0.67 (0.41,1.10)	0.69 (0.42,1.13)	0.62 (0.37,1.04)	0.67 (0.41,1.10)
			P-interaction= 0.45	P-interaction= 0.09	P-interaction= 0.52	P-interaction= 0.60
BMI	<25 kg/m^2	2,932/48,578	0.47 (0.21,1.09)	0.49 (0.21,1.15)	0.51 (0.21,1.20)	0.46 (0.20,1.06)
	≥25 kg/m^2	6,924/71,663	0.72 (0.46,1.12)	0.75 (0.48,1.19)	0.59 (0.36,0.96)	0.73 (0.46,1.13)
			P-interaction= 0.60	P-interaction= 0.71	P-interaction= 0.65	P-interaction= 0.27
Abdominal obesity	No	6,234/80,518	0.44 (0.26,0.73)	0.45 (0.27,0.76)	0.44 (0.25,0.75)	0.43 (0.26,0.73)
	Yes	3,639/29,952	1.05 (0.56,1.94)	1.12 (0.60,2.10)	0.78 (0.40,1.53)	1.06 (0.57,1.97)
			P-interaction= 0.08	P-interaction= 0.08	P-interaction= 0.042	P-interaction= 0.09
Baseline hypertension	No	5,723/85,710	0.58 (0.35,0.97)	0.59 (0.35,0.98)	0.48 (0.28,0.82)	0.59 (0.35, 0.98)
	Yes	4,167/24,896	0.74 (0.40,1.34)	0.82 (0.44,1.50)	0.79 (0.41,1.50)	0.72 (0.39, 1.32)
			P-interaction= 0.29	P-interaction= 0.29	P-interaction= 0.039	P-interaction= 0.71
Baseline hypercholesterolemia	No	7,252/97,661	0.59 (0.37,0.93)	0.63 (0.39,0.99)	0.52 (0.32,0.85)	0.59 (0.37,0.92)
	Yes	2,638/12,945	0.81 (0.38,1.74)	0.81 (0.37,1.77)	0.80 (0.35,1.82)	0.81 (0.38,1.74)
			P-interaction= 0.36	P-interaction= 0.56	P-interaction= 0.33	P-interaction= 0.31
Baseline diabetes	No	9,302/108,434	0.64 (0.43,0.97)	0.67 (0.44,1.01)	0.59 (0.38,0.90)	0.64 (0.43,0.96)
	Yes	588/2,172	0.58 (0.14,2.33)	0.63 (0.15,2.63)	0.43 (0.08,2.16)	0.58 (0.14,2.32)
			P-interaction= 0.40	P-interaction= 0.52	P-interaction= 0.90	P-interaction= 0.12

**Hazard ratios (95% confidence intervals) were derived from Cox proportional hazard regression models, adjusted for sex (female/males), age (y), ethnic background (white Europeans/south Asians/African Caribbean/multiple ethnic background or other/unknown), education (college or university degree or vocation/national examination at 17-18 years of age/national examination at 16 years of age/unknown), Townsend deprivation index (quintiles), country of assessment centre (England/Scotland/Wales), smoking (current/former/never/unknown), physical activity (low/moderate/high/unknown), alcohol intake (non- drinkers/<1 g/d/1-10 g/d/10-20 g/d/≥20 g/d/unknown), dietary supplement use (yes/no), hormone replacement therapy use (females only) (yes/no/unknown), menopausal status (females only) (yes/no/not sure – had a hysterectomy/not sure -other reason/unknown), fruit and vegetables (g/d), fibre (g/d), family history of CVD (yes/no), BMI (underweight/healthy weight/overweight/obesity/unknown), abdominal obesity (yes/no/unknown), baseline hypertension (yes/no), baseline hypercholesterolemia (yes/no) and baseline diabetes (yes/no), trans unsaturated fatty acids (en%), dietary cholesterol (mg/d), protein (en%), carbohydrate (en%), monounsaturated fatty acids (en%), polyunsaturated fatty acids (en%), saturated fatty acids from non-meat and non-dairy sources (en%). Saturated fatty acids from meat and dairy sources were mutually adjusted for in the analyses, as appropriate. Abbreviations. BMI=body mass index. CVD=cardiovascular diseases. SFA=saturated fatty acids.*

Table S24. Hazard ratios (95% confidence intervals) for risk of cardiovascular diseases associated with substitution of saturated fatty acids from one type of meat with another in the UK Biobank Study (n=120,496), stratified by sex, age, body mass index, abdominal obesity, baseline hypertension, baseline hypercholesterolemia, baseline diabetes

Effect modifier	Strata	N cases/total	Hazard ratio (95% confidence intervals) for CVD risk associated with the substitution of SFA (2.5 en%) from*			
			**Processed meat with**	**Processed red meat with**	**Processed poultry with**	**Red meat with**
			**Unprocessed meat**	**Unprocessed red meat**	**Unprocessed poultry**	**Poultry**
Sex	Females	3,879/68,689	0.86 (0.69,1.06)	0.93 (0.79,1.10)	0.60(0.29,1.22)	0.84(0.64,1.09)
	Males	6,011/51,807	0.87 (0.74,1.02)	0.99 (0.88,1.12)	0.62 (0.37,1.04)	1.07(0.86,1.32)
			P-interaction=0.48	P-interaction=0.53	P-interaction=0.30	P-interaction=0.20
Age	<57 years	2,455/58,268	0.85 (0.66,1.09)	0.99 (0.82,1.19)	0.61 (0.30,1.24)	0.97 (0.72,1.33)
	≥57 years	7,435/62,228	0.87 (0.75,1.01)	0.96 (0.86,1.08)	0.63 (0.38,1.07)	0.97 (0.79,1.18)
			P-interaction=0.07	P-interaction=0.06	P-interaction=0.53	P-interaction=0.07
BMI	<25 kg/m^2	2,932/48,578	0.83 (0.64,1.07)	0.99 (0.81,1.21)	0.41 (0.17,0.99)	0.96 (0.69,1.33)
	≥25 kg/m^2	6,924/71,663	0.89 (0.77,1.03)	0.97 (0.87,1.09)	0.71 (0.44,1.14)	0.97 (0.80,1.17)
			P-interaction=0.90	P-interaction=0.73	P-interaction=0.72	P-interaction=0.91
Abdominal obesity	No	6,234/80,518	0.84 (0.71,0.99)	0.99 (0.87,1.12)	0.39 (0.22,0.67)	1.02 (0.82,1.27)
	Yes	3,639/29,952	0.93 (0.76,1.13)	0.97 (0.83,1.13)	1.10 (0.57,2.13)	0.89 (0.69,1.15)
			P-interaction=0.80	P-interaction=0.44	P-interaction=0.05	P-interaction=0.78
Baseline hypertension	No	5,723/85,710	0.87 (0.73,1.03)	0.97 (0.85,1.11)	0.61 (0.35,1.06)	1.05 (0.84,1.31)
	Yes	4,167/24,896	0.86 (0.71,1.04)	0.98 (0.84,1.13)	0.61 (0.32,1.17)	0.87 (0.68,1.12)
			P-interaction=0.88	P-interaction=0.92	P-interaction=0.79	P-interaction=0.99
Baseline	No	7,252/97,661	0.86 (0.74,1.00)	0.96 (0.86,1.08)	0.59 (0.36,0.96)	1.02 (0.84,1.24)
hypercholesterolemia	Yes	2,638/12,945	0.89 (0.70,1.13)	1.00 (0.83,1.20)	0.67 (0.29,1.51)	0.84 (0.61,1.16)
			P-interaction=0.47	P-interaction=0.54	P-interaction=0.62	P-interaction=0.79
Baseline diabetes	No	9,302/108,434	0.86 (0.75,0.98)	0.97 (0.88,1.07)	0.63 (0.41,0.98)	0.99 (0.83,1.17)
	Yes	588/2,172	0.89 (0.54,1.45)	0.94 (0.65,1.36)	0.36 (0.08,1.67)	0.81 (0.42,1.55)
			P-interaction=0.59	P-interaction=0.74	P=interaction=0.77	P-interaction=0.84

**Hazard ratios (95% confidence intervals) were derived from Cox proportional hazard regression models, adjusted for sex (female/males), age (y), ethnic background (white Europeans/south Asians/African Caribbean/multiple ethnic background or other/unknown), education (college or university degree or vocation/national examination at 17-18 years of age/national examination at 16 years of age/unknown), Townsend deprivation index (quintiles), country of assessment centre (England/Scotland/Wales), smoking (current/former/never/unknown), physical activity (low/moderate/high/unknown), alcohol intake (non- drinkers/<1 g/d/1-10 g/d/10-20 g/d/≥20 g/d/unknown), dietary supplement use (yes/no), hormone replacement therapy use (females only) (yes/no/unknown), menopausal status (females only) (yes/no/not sure – had a hysterectomy/not sure -other reason/unknown), fruit and vegetables (g/d), fibre (g/d), family history of CVD (yes/no), BMI (underweight/healthy weight/overweight/obesity/unknown), abdominal obesity (yes/no/unknown), baseline hypertension (yes/no), baseline hypercholesterolemia (yes/no) and baseline diabetes (yes/no), trans unsaturated fatty acids (en%), dietary cholesterol (mg/d), protein (en%), carbohydrate (en%), monounsaturated fatty acids (en%), polyunsaturated fatty acids (en%), saturated fatty acids from non-meat sources (en%), saturated fatty acids from other meats (offal) (en%). Saturated fatty acids from meat sources were mutually adjusted for in the analyses, as appropriate. Abbreviations. BMI=body mass index. CVD=cardiovascular diseases. SFA=saturated fatty acids.*

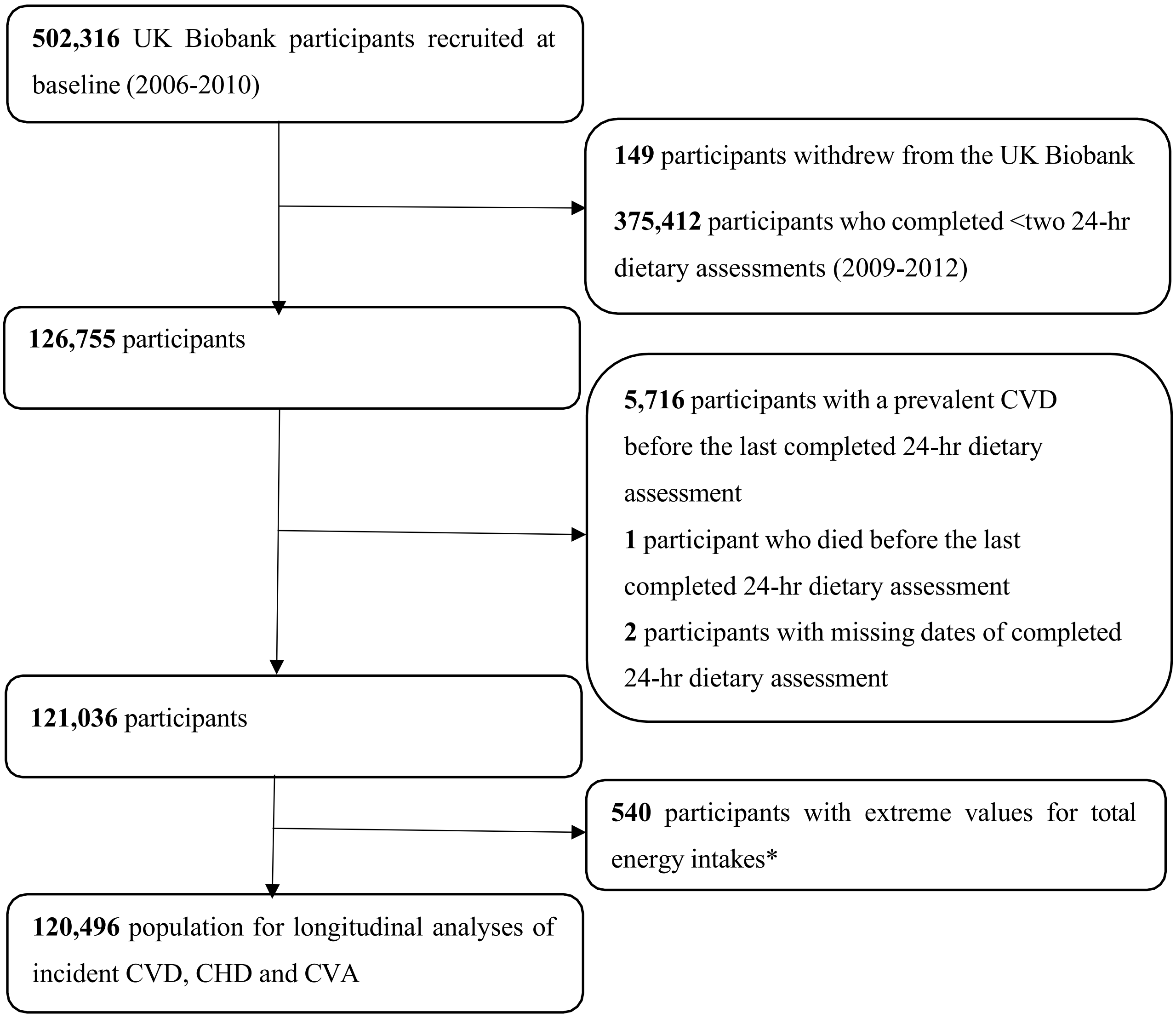

Figure S1. Flowchart for the derivation of the study sample from the UK Biobank Study

*Participants with total energy intakes (based on the mean of all estimates of energy intake from two or more 24-hr dietary assessments) outside the range of 500-3500 kcal/day for women and 800-4200 kcal/day for men were excluded from analyses. Abbreviations. CVD=cardiovascular diseases; CHD=coronary heart diseases; CVA=cerebrovascular diseases.

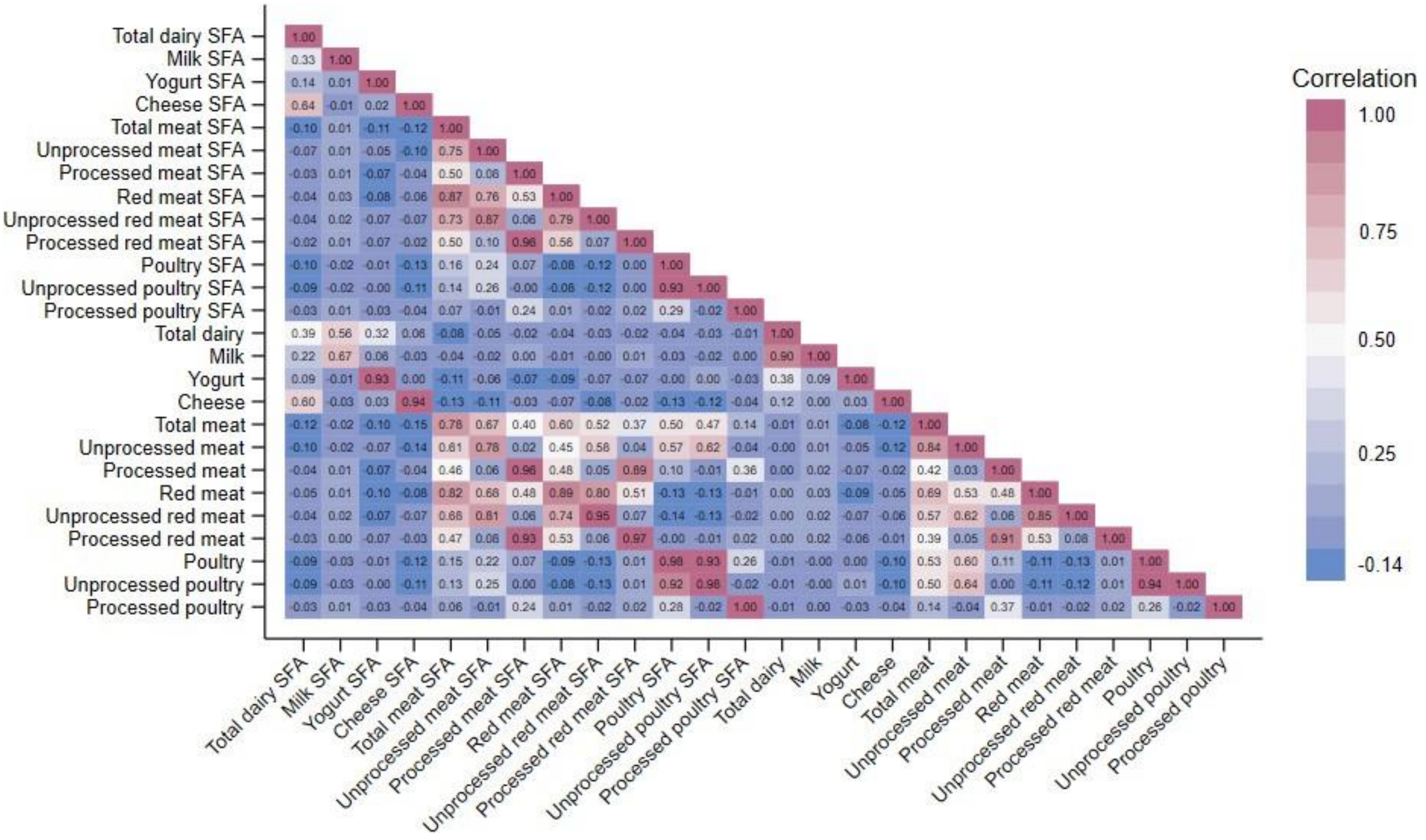

Figure S2. Spearman correlation coefficients (rs) for estimates of meat- and dairy-specific saturated fatty acids (SFA) and estimates of meat and dairy categories among participants in the UK Biobank Study (n=120,496), 2009-2012

References

1. Liu, B., et al., *Development and evaluation of the Oxford WebQ, a low-cost, web-based method for assessment of previous 24 h dietary intakes in large-scale prospective studies.* Public Health Nutr, 2011. **14**(11): p. 1998-2005.
2. Greenwood, D.C., et al., *Validation of the Oxford WebQ Online 24-Hour Dietary Questionnaire Using Biomarkers.* Am J Epidemiol, 2019. **188**(10): p. 1858-1867.
3. Zhang, H., et al., *Meat consumption and risk of incident dementia: cohort study of 493,888 UK Biobank participants.* Am J Clin Nutr, 2021. **114**(1): p. 175-184.
4. Perez-Cornago, A., et al., *Description of the updated nutrition calculation of the Oxford WebQ questionnaire and comparison with the previous version among 207,144 participants in UK Biobank.* Eur J Nutr, 2021. **60**(7): p. 4019-4030.
5. Piernas, C., et al., *Describing a new food group classification system for UK biobank: analysis of food groups and sources of macro- and micronutrients in 208,200 participants.* Eur J Nutr, 2021. **60**(5): p. 2879-2890.
6. Bradbury, K.E., et al., *Association between physical activity and body fat percentage, with adjustment for BMI: a large cross-sectional analysis of UK Biobank.* BMJ Open, 2017. **7**(3): p. e011843.
7. Grundy, S.M., et al., *Diagnosis and management of the metabolic syndrome: an American Heart Association/National Heart, Lung, and Blood Institute Scientific Statement.* Circulation, 2005. **112**(17): p. 2735-52.
